# The genome of *Cleistogenes songorica* provides a blueprint for functional dissection of dimorphic flower differentiation and drought adaptability

**DOI:** 10.1111/pbi.13483

**Published:** 2020-10-28

**Authors:** Jiyu Zhang, Fan Wu, Qi Yan, Ulrik P John, Mingshu Cao, Pan Xu, Zhengshe Zhang, Tiantian Ma, Xifang Zong, Jie Li, Ruijuan Liu, Yufei Zhang, Yufeng Zhao, Gisele Kanzana, Yanyan Lv, Zhibiao Nan, German Spangenberg, Yanrong Wang

**Affiliations:** ^1^ State Key Laboratory of Grassland Agro‐ecosystems Key Laboratory of Grassland Livestock Industry Innovation, Ministry of Agriculture and Rural Affairs Engineering Research Center of Grassland Industry, Ministry of Education College of Pastoral Agriculture Science and Technology Lanzhou University Lanzhou China; ^2^ Agriculture Victoria Research, Department of Jobs, Precincts and Regions AgriBio, Centre for AgriBioscience, La Trobe University Victoria Australia; ^3^ AgResearch Limited, Grasslands Research Centre Palmerston North New Zealand; ^4^ Key Laboratory of Adaptation and Evolution of Plateau Biota Northwest Institute of Plateau Biology Chinese Academy of Sciences Xining China

**Keywords:** *Cleistogenes songorica*, genome assembly, allotetraploid, dimorphic flower, cleistogamy, drought tolerance

## Abstract

*Cleistogenes songorica* (2*n* = 4*x* = 40) is a desert grass with a unique dimorphic flowering mechanism and an ability to survive extreme drought. Little is known about the genetics underlying drought tolerance and its reproductive adaptability. Here, we sequenced and assembled a high‐quality chromosome‐level *C. songorica* genome (contig N50 = 21.28 Mb). Complete assemblies of all telomeres, and of ten chromosomes were derived*. C*. *songorica* underwent a recent tetraploidization (~19 million years ago) and four major chromosomal rearrangements. Expanded genes were significantly enriched in fatty acid elongation, phenylpropanoid biosynthesis, starch and sucrose metabolism, and circadian rhythm pathways. By comparative transcriptomic analysis we found that conserved drought tolerance related genes were expanded. Transcription of *CsMYB* genes was associated with differential development of chasmogamous and cleistogamous flowers, as well as drought tolerance. Furthermore, we found that regulation modules encompassing miRNA, transcription factors and target genes are involved in dimorphic flower development, validated by overexpression of *CsAP2_9* and its targeted miR172 in rice. Our findings enable further understanding of the mechanisms of drought tolerance and flowering in *C. songorica,* and provide new insights into the adaptability of native grass species in evolution, along with potential resources for trait improvement in agronomically important species.

## Introduction

In the face of global environmental variability, food security is critical to the feeding of upwards of 10 billion people by 2050 (Tester and Langridge, 2010). Two‐thirds of the world's food is produced on unirrigated land (Herrero *et al*., 2013). Global climate change is predicted to greatly increase the prevalence and severity of drought (Dai, 2012). Drought is arguably the major and enduring environmental threat to crop and pasture production. Consequently, we need to enhance the tolerance of water limitation in crops, to improve or maintain crop yields. One means we can achieve this is to expand the germplasm base by accessing novel genetic diversity to accelerate breeding. Native grass germplasm potentially contains substantial gene resources to be characterized and then exploited.

*Cleistogenes songorica* is an important perennial forage, and ecologically significant C_4_ grass in temperate saline, semi‐arid and desert areas in central Asia where average annual rainfall is below 110 mm. It is widely distributed in northwest China, Mongolia and eastern Russia (Li *et al*., 2014). With a strong root system, Cs has found application in desert ecosystem and grassland restoration by stabilizing soil structure and reducing soil erosion (Niu and Nan, 2017). It can reduce evaporation and recover from prolonged periods of drought, when only 38.5% leaf relative water content, and 3.3% soil gravimetric water content remain (Yan *et al*., 2019b; Zhang *et al*., 2011).

**Figure 1:**
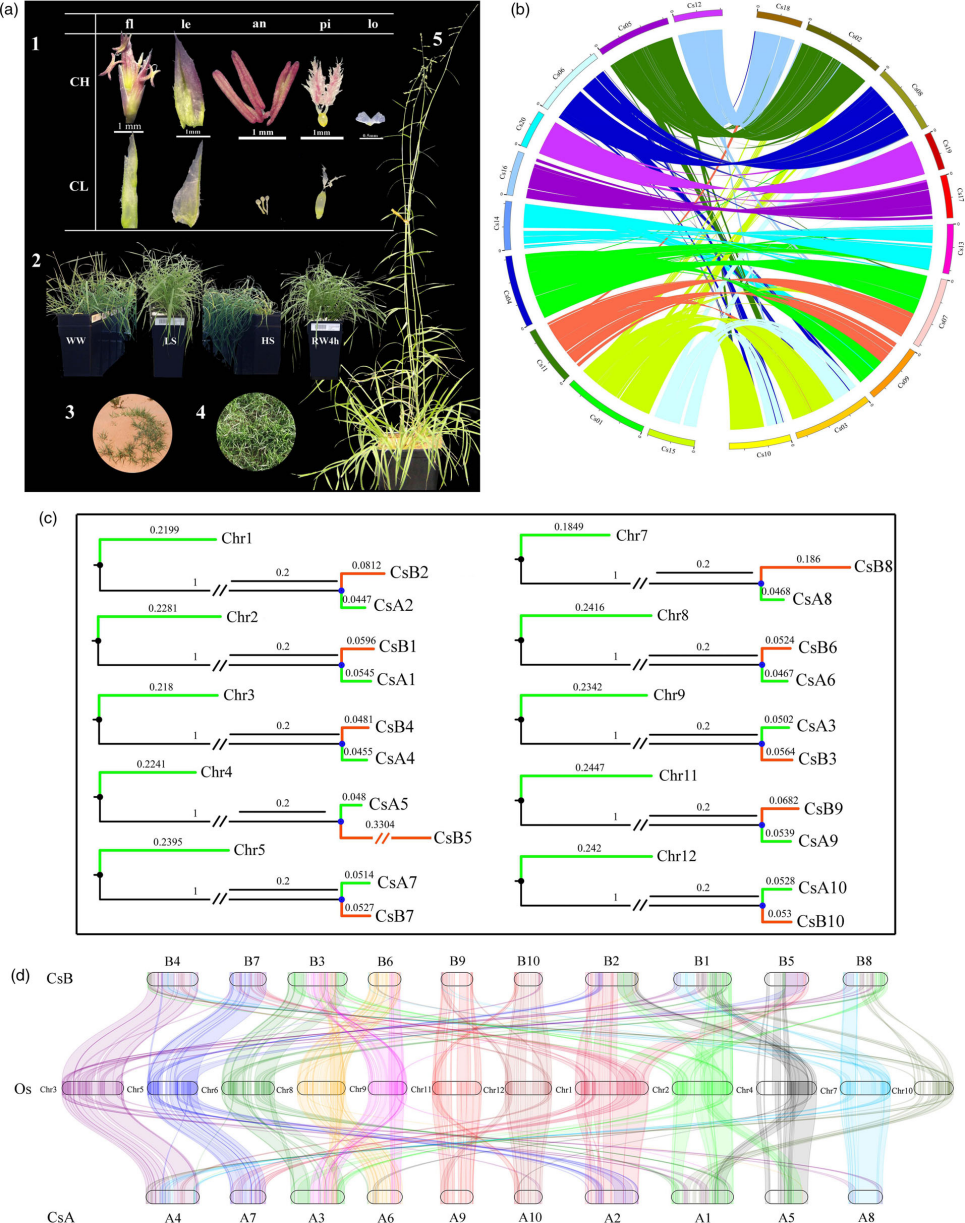
Characterization of the tetraploid *C. songorica* genome. (a) Morphology of Cs. 1: Structure of chasmogamy (CH) and cleistogamy (CL) flowers: flower (fl), lemma (le), anther (an), pistil (pi) and lodicule (lo). 2: Response to drought treatments, well‐watered (WW), low‐level stress (LS), high‐level stress (HS) and 4hrs after re‐watering (RW4h). 3: Wild‐type plants in native habitat. 4: Domesticated lawn cultivar 'Tenggeli'. 5: Flowering plant grown in greenhouse. (b) Inter‐chromosomal synteny. (c) Phylogenetic tree based on single‐copy orthologs shows chromosomal relationships between *C. songorica* (Cs) and *O. sativa* (Os). (d) Synteny of Cs and Os chromosomes. Homologous chromosome pairs, with corresponding pseudochromosome numbering in parenthesis: A1‐B1 (Cs01‐Cs03), A2‐B2 (Cs04‐Cs07), A3‐B3 (Cs05‐Cs02), A4‐B4 (Cs06‐Cs08), A5‐B5 (Cs11‐Cs09), A6‐B6 (Cs12‐Cs18), A7‐B7 (Cs14‐Cs13), A8‐B8 (Cs15‐Cs10), A9‐B9 (Cs16‐Cs17) and A10‐B10 (Cs20‐Cs19).

*C. songorica* serves as a natural forage source in harsh environments largely because of its dimorphic flowering mechanism, which allows it to survive and reproduce under extreme conditions. It develops two types of inflorescences in a single plant, enabling open pollination (chasmogamy, CH) on the top panicle and self‐pollination (cleistogamy, CL) on spike flowers embedded in the leaf sheath at each node (Figure 1a) (Wu *et al*., 2018). Whilst open pollination allows maintenance of genetic variation, self‐pollination ensures reproductive success under drought. In response to drought stress, some plants progress precociously into the reproductive stage (Aronson *et al*., 1992), whilst others repress heading but strive to maintain both vegetative and reproductive growth.

In respect of native grass species, the genomes of *Dactylis glomerata* (Huang *et al*., 2020), *Br* *achypodium distachyon* (Vogel *et al*., 2010) and *Oropetium thomaeum* (Vanburen *et al*., 2015) have been sequenced. Genome sequencing of *C. songorica* will facilitate an understanding of the genetics underlying its drought tolerance and reproductive adaptability. The paucity of genetic information in Cs has hindered the accurate characterization of new germplasm for developing forage cultivars. Genome‐scale information from plants adapted to extreme environments has facilitated the breeding of drought tolerant crops (Cattivelli *et al*., 2008); the understanding of water‐use efficient photosynthesis via crassulacean acid metabolism (Yang *et al*., 2017) and insights into desiccation tolerance in xerophytic plants (Costa *et al*., 2017). We report here a high‐quality whole‐genome sequence and annotation of Cs (2*n* = 4*x* = 40) by means of the integration of data from varied sequencing platforms and strategies, including Illumina short reads, PacBio long reads and Hi‐C. RNA‐seq‐based transcriptomics was utilized to optimize gene structure prediction and annotation, and to identify genes differentially expressed between dimorphic flowers, and upon water stress treatments. This report lays a foundation for studies of the genetic basis of many unique or complex traits in this species. Along with genome resources of other drought tolerant plants (Costa *et al*., 2017; VanBuren *et al*., 2015), the gene‐rich Cs genome provides a genetic blueprint for elucidating the mechanisms by which this plant survives prolonged dehydration, and is able to flourish in a harsh environment.

## Results

### Genome assembly and annotation

*C. songorica* (Cs) has 40 chromosomes (Figure S1) but unknown ploidy. Its genome size was estimated to be 552 Mb by flow cytometry (Figure S2). We applied both Illumina short‐read sequencing and PacBio single‐molecule real‐time (SMRT) sequencing platforms to independently sequence and assemble genomes. Two paired‐end sequencing libraries (with insert sizes of 200 and 450 bp) and two mate‐paired sequencing libraries (with insert sizes of 1 and 2 kb) were constructed and sequenced using the Illumina platform. Based on 17‐mer analysis (Figure S3), the genome size was estimated to be 541.95 Mb, with a heterozygosity of 0.16% (Table S1). A SMRT CLR (continuous long reads) library (40 kb) was constructed, and 172 Gb (~316× coverage) raw data were generated using the PacBio Sequel Ⅱ System. The contig‐level assembly, using PacBio long reads by the Falcon 0.3.0 package, comprised 540.12 Mb of the genome, with a contig N50 of 21.28 Mb (Table S2). To anchor and orient the contigs onto chromosomes, we constructed a Hi‐C library. A total of 43.41 Gb of clean data were obtained and analysed with HiC‐Pro (Servant *et al*., 2015). 528.52 Mb of contigs (35 contigs, 97.85% coverage) were anchored to 20 pseudochromosomes, ten of them having no gaps (Figure S4, Table S3). The telomeres of all 20 chromosomes were assembled, comprising tandem repeat elements of two motifs: 5’‐TTTAGGG‐3’, and 5’‐CCCTAAA‐3’ (Table 1).

**Table 1:** Statistics of *C. songorica* genome assembly

Chromosome	Size (Mb)	Number of contigs	Telomere (Number of tandem duplicates)	
			Start (5' CCCTAAA)	End (3' TTTAGGG)
Chr01	38.88	3	711	708
Chr02	35.15	3	712	690
Chr03	34.42	1	709	714
Chr04	32.95	3	697	692
Chr05	32.29	1	713	510
Chr06	31.53	2	705	712
Chr07	31.02	1	694	702
Chr08	30.48	1	702	708
Chr09	28.48	1	698	664
Chr10	26.39	1	688	707
Chr11	26.02	2	698	709
Chr12	22.85	3	706	708
Chr13	22.30	2	714	693
Chr14	21.28	1	703	713
Chr15	20.37	2	700	663
Chr16	20.35	3	705	707
Chr17	20.14	1	690	691
Chr18	19.46	1	698	709
Chr19	17.27	2	695	698
Chr20	16.87	1	714	670
Unanchored	11.60	83	–	–
Total	540.12	118	–	–

To correct long‐read sequencing errors, Illumina reads (paired‐end libraries, 450 bp) were mapped to the assembly, resulting in 99.84% of short reads mapping to the genome assembly, with a 10x coverage of 97.95% (Table S4). The assembled genome was assessed using BUSCO (Benchmarking Universal Single‐Copy Orthologs) (Simão *et al*., 2015). As a result, 98.25% of the 1375 ubiquitous genes in embryophytes were identified, demonstrating the completeness of the genome assembly and annotation (Table S5). As a validation of the assembly quality, flowering transcripts and drought‐responsive transcripts were mapped to the assembled contigs, achieving coverage rates of 98% and 90%, respectively (Table S6).

The genome has a relatively high GC content (45.02%), near the upper limit of the range (33.6% to 48.9%) in monocots (Šmarda et al., 2014) (Table S1, Figure S5). High GC content has been reported associated with plant adaptation to abiotic stress (Costa *et al*., 2017).

We identified 54 383 protein‐coding genes (89.48% annotated) in Cs (Table S7). On average, protein‐coding genes are 3450 bp long, and with 5 exons per gene. The average exon size is 137 bp (Table S8). The length of mRNA, coding DNA sequence (CDS), introns, exons and the number of exons per gene are similar to that in other grass species (Figure S6). We also identified 287 miRNA, 3397 long non‐coding RNAs (lncRNAs), 1139 tRNAs, 580 rRNAs and 932 snRNAs in Cs (Tables S3 and S9).

Repetitive sequences and transposon elements (TE) were analysed using Repbase and *De novo* (Table S10). DNA transposons account for 10.34% of the genome. Among retrotransposons, LTRs, LINEs and SINEs account for 26.54%, 4.16% and 0.023%, respectively (Figures S7 and S8, Table S11).

We concluded that the Cs under study was an allotetraploid (2*n* = 4*x* = 40) based on: (I) k‐mer analysis as shown in Figure S3, where two peaks were present, with the smaller peak at the doubled multiplicity (peak depth ~80) of the major peak (Yasui *et al*., 2016); (II) the high degree of colinearity among the 20 assembled pseudochromosomes; and (III) the phylogenomic analysis of Cs relative to *Oryza sativa* and *Oropetium thomaeum*, described below.

### Within genome analyses and grass genome evolution

High inter‐chromosomal colinearity among the 20 pseudochromosomes (*n* = 20) (Figure 1b) strongly suggests the existence of sub‐genomes in Cs. To investigate the structure of the Cs sub‐genomes, we conducted a phylogenomics study between *O. sativa* (Os, 2*n* = 2*x* = 24) genomes and Cs pseudochromosomes. We found each Os chromosome corresponded with a pair of Cs pseudochromosomes (Figure 1c and d). Ten pseudochromosomes 1, 4, 5, 6, 11, 12, 14, 15, 16 and 20 with a closer genetic relationship with the ten Os chromosomes were classed into one sub‐genome (A: 268.3 Mb, Figure 1c and Table S12). Thus, we assigned and denoted the assembled 20 chromosomes as A1‐10 and B1‐10. Chromosomal translocations are evident (Figure 1d, Figure S9), with Cs chromosomes B1, B2, B5 and B8 exhibiting higher levels of structural variation. The intra‐genome syntenic analysis showed that B5 had regions syntenic with A2, along with B2. Likewise, B1 and B8 had regions syntenic with A1, indicating chromosomal rearrangements had occurred between B2 and B5, and B1 and B8 (Figure S9).

**Figure 2:**
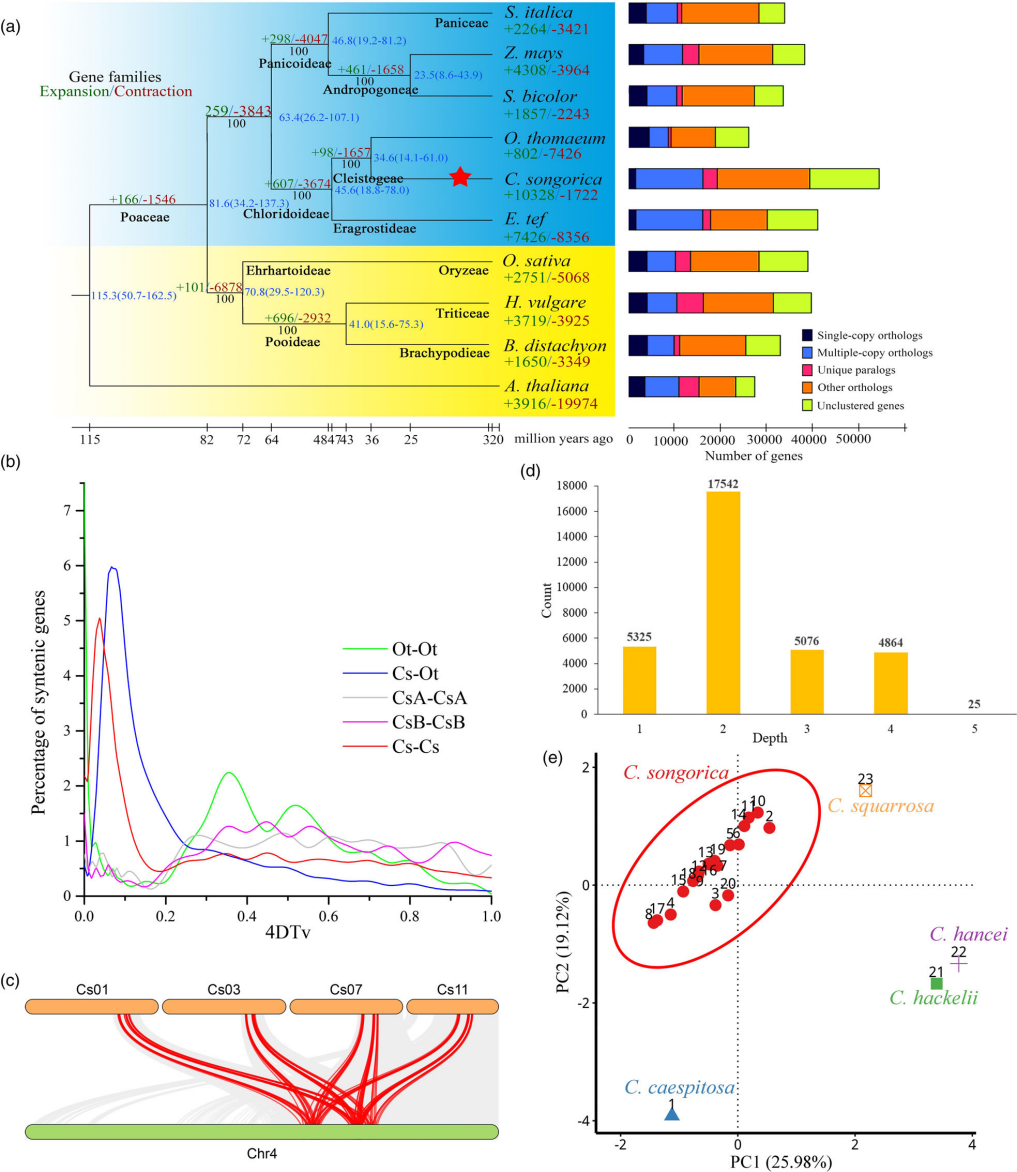
Evolution of the *C. songorica* genome. (a) Phylogenetic relationship of Cs with nine other plant species. Blue background: C_4_ species; yellow: C_3_ species. Divergence times labelled in blue; gene family expansion and contraction enumerated below the species names in green and red; whole‐genome duplication event indicated by red star; Gene categories used from all the species are shown on the right. (b) 4DTv values (yellow line) reveal a recent WGD of Cs. c, A typical micro‐colinearity pattern between genomic regions of Cs and Os, where grey lines represent one‐to‐one gene synteny and red lines represent one Os gene corresponding to four Cs genes. (d) Syntenic depth based on mapping between Cs and Os genes. (e) Principal component analysis of 23 *Cleistogenes* accessions with 1119 genetic markers.

In addition, we selected four larger scaffolds from *O. thomaeum* (Ot) to map onto Cs chromosomes (Figure S10), given the close phylogenetic relationship between Cs and Ot (2*n* = 2*x* = 20) (Figure 2a). This demonstrated that the diploid Ot (*x* = 10) has a closer genetic relationship with the Cs A sub‐genome, further validating the allocation of Cs sub‐genomes inferred from the Os results.

Comparative genomic analyses of Cs with eight Poaceae species were performed, using *Arabidopsis thaliana* (At) as an outgroup (Figure 2a). Based on 882 single‐copy orthologs (Data S1), phylogenomic analysis (PhyML) revealed that Cs, Ot and *Eragrostis tef* (Et) formed a clade (Chloridoideae), with Cs more closely related to Ot than Et. The more distant relationship between Cs and Et was supported by morphology‐based studies, demonstrating Cs is not a member of Eragrostideae (Lin, 2008; Yu, 2017). We estimated Cs and Et diverged about 45.6 million years ago (Mya), and Cs and Ot about 34.0 Mya.

Distributions of synonymous substitutions per site (Ks) of Cs homologous gene pairs showed one prominent peak around Ks = 0.25, reflecting a whole‐genome duplication (WGD) event that occurred around 19.2 Mya (Figure S11). Fourfold transversion rate (4DTv) analysis indicated that the tetraploidization event occurred after the divergence from Ot (Figure 2b). We found a total of 417 syntenic blocks (32 832 pairs of collinear genes) between Cs and Os (Figure S12a), and 461 syntenic blocks (containing 29 766 pairs of collinear genes) between Cs and Ot (Figure S12b). Syntenic depth analysis indicated that typically there are multiple Os genes aligned to one Cs gene (Figure 2c). When comparing the diploid Os and Ot genomes, the syntenic depth is predominantly 2x (Figure 2c and Figure S13a), probably due to the two homeologous genomes in Cs. The presence of regions of 3x and 4x syntenic depth (Figure 2d) indicate the presence of segmental duplications in the two sub‐genomes. The microsyntenic patterns further support the postulated tetraploidization event, as a 1:4 ratio of genes was observed in comparisons of the Ot‐Cs, and Os‐Cs genomes (Figure 2d, Figure S13b‐f, Data S2). KEGG enrichment analysis showed that these genes were significantly enriched in the categories brassinosteroid biosynthesis, limonene and pinene degradation, plant–pathogen interaction, synthesis and degradation of ketone bodies, and circadian rhythm in plants (Table S13).

A high‐quality genome enables the development of accurate genetic markers to characterize and exploit novel germplasm for breeding. We developed genetic markers from long terminal repeat retrotransposons (LTRs), introns (intron‐length polymorphic) and miRNAs, to study the genetic diversity of Cs and its relatives. Based on the 1119 SSR markers (Figure S14), we characterized the genetic relationships of 23 *Cleistogenes* accessions. Four clusters were resolved, revealing that *C. hackelii* and *C. hancei* are genetically close to, and *C. squarrosa* and *C. caespitosa,* are distant from, Cs accessions (Figure 2e and Figure S15).

### Gene family expansion and contraction

Gene family expansion has been linked to plant stress adaptation (Zeng *et al*., 2019). Using OrthoMCL 39 396 Cs genes were clustered into 19 252 gene families (Figure S16 and Data S3), of which 10 328 were expanded and 1722 contracted (Figure 2a). Furthermore, 6174 gene families were shared among the ten species recruited for the comparative genomic study (see the section above), whilst 1195 were unique to Cs (Data S3). Cs unique gene families were enriched in the functional categories circadian rhythm, brassinosteroid biosynthesis and phenylpropanoid biosynthesis (Figure S17a and Table S14). The functional categories significantly enriched in the expanded genes included brassinosteroid biosynthesis, fatty acid elongation, phenylpropanoid biosynthesis, starch and sucrose metabolism, and circadian rhythm (Figure S17b). These unique and expanded genes may contribute in part to the adaptability of Cs.

As a case study, we analysed the evolution and expression of the phosphoenolpyruvate carboxylase (PEPC) gene family in Cs because of its specific role in photosynthetic CO_2_ fixation in C_4_ plants. The PEPC family is expanded in Cs compared to *Zea mays* (Zm) and Os. PEPC peptides characteristically have a conserved alanine residue (A) in C_3_, and a serine residue (S) at the corresponding position in C_4_ (Christin *et al*., 2007). We identified five C_3_ PEPC genes and two C_4_ PEPC genes in Cs. A phylogenetic tree showed that C_4_ PEPCs were clustered together (Figure S18a). Multiple alignments of 21 PEPC peptides from various species showed six conserved motifs (Figure S18b), with the known feature (A/S substitution) of C_3_/C_4_ plants observed at position 35 in motif 2. (Figure S18c). Only two CsPEPCs (CCG0014840.1, CCG321851) and one ZmPEPC (Zm00008a034314_T01) have S at position 35, whilst all others have A (Figure S18d). The two C_4_ Cs*PEPC* genes were highly expressed in leaf and shoot, but low in root. The five C_3_ Cs*PEPC* genes had low‐level expression in all tissues (Figure S19a). In response to stress treatments, C_4_ Cs*PEPC* genes maintained very high‐level expression under all stresses except high temperature (Figure S19b). The low level of C_4_ Cs*PEPC* expression under high temperature may be protective, by modulating photosynthesis under high stress. The two C_4_ *PEPCs* are located on homologous chromosomes A3 and B3 (Figure S20). C_3_ and C_4_ *PEPCs* showed strong colinearity, and all the *PEPC* genes have undergone purification selection indicated by the Ka/Ks values (<1) for all homologous gene pairs (Figure S20, Table S15).

### Genome‐wide expression dominance

To investigate sub‐genome‐specific gene expression, we conducted transcriptional analyses of homologous genes in shoot and root, under stress treatments (Methods S1). In total, 18 820 genes showed expression dominance in all the treatments, with 51.6% from the B genome preferentially expressed (Figures S21 and 22). Similar distributions of dominance genes and neutral genes were also observed for different treatments (Figures S8 and 22). The density of transposons and retrotransposons (LINE) is higher in the B than in the A sub‐genome (2.7% vs 1.8%), *Gypsy* and *Copia* are higher in the A (3.2%) than in the B sub‐genome (1.1%) (Figure S8). A prevailing explanation for the preferential expression of homeologs is that adjacent transposon elements (TEs) may repress gene expression. Gene expression in At is negatively correlated with the density of surrounding methylated TEs (Bottani *et al*., 2018; Hollister and Gaut, 2009). Consistent with previous studies, TEs of Cs are mainly distributed pericentromerically, whilst potential dominance exhibiting genes are located more distally (Figure S22). We postulate that the absence of significant global genome dominance between the A and B genomes in Cs is due in part to the relative absence of differential TE distribution.

### Expanded and conserved gene families associated with stress adaptation

**Figure 3:**
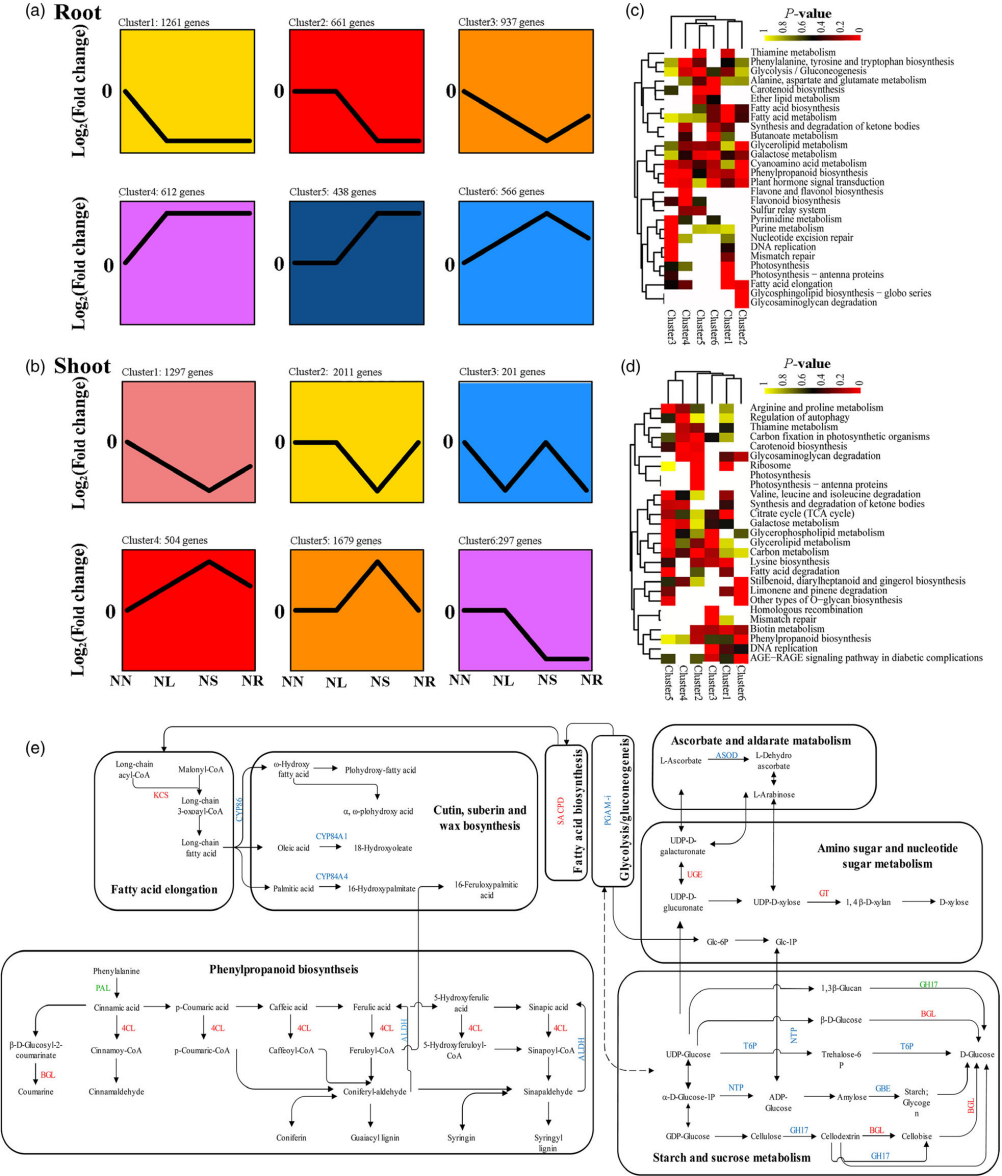
Expression patterns and enrichment analysis of DEGs under water stress and recovery. (a–b) Cluster analysis of DEGs displaying a log2 fold change (with absolute value >2) of transcripts during water stress and recovery, in root and shoot, respectively. The comparisons include control vs control (NN), control vs light water stress (NL), control vs severe water stress (NS) and control vs recovery (NR). (c–d) Heat maps of significantly enriched pathways during water stress and recovery. The yellow and red colours indicate the *Q*‐value for significantly enriched pathways. (e) Enrichment of expanded and conserved genes in metabolic pathways. Green: expanded genes; blue: conserved genes; red: both expanded and conserved. Abbreviations: PGAM‐I, 2,3‐bisphosphoglycerate‐independent phosphoglycerate mutase; SACPD, stearoyl‐acyl carrier protein desaturase 2; CYP, cytochrome P450; 4CL, 4‐coumarate‐‐CoA ligase; BGL, Beta‐glucosidase; ALDH, aldehyde dehydrogenase; ASOD, ascorbate oxidase; GT, glycosyl transferase; UGE, UDP‐glucuronate 4‐epimerase; NTP, nucleotidyl transferase; T6P, trehalose 6‐phosphate synthase/phosphatase; GBE, 1,4‐alpha‐glucan branching enzyme; PAL, phenylalanine ammonia‐lyase; GH17, glycosyl hydrolase 17; KCS, β‐ketoacyl‐CoA synthases.

Significantly expanded gene families may harbour genes adaptive for stress. For example, 1472 genes from 102 expanded families were significantly enriched in metabolic pathways implicated in stress responses, including fatty acid elongation, phenylpropanoid biosynthesis and starch and sucrose metabolism (Figure 3a–d).

To investigate the genetic mechanisms underlying drought tolerance, we performed transcriptomic analysis under four water stress conditions (Figure 1a‐2) in both shoot and root (Figure S23). Co‐expression analysis showed that the 4475 and 5911 differentially expressed genes (DEGs) formed six major clusters in root and shoot, respectively (Figure 3a‐d). In general, down‐regulated genes were related to energy metabolism and photosynthesis (shoot cluster 2), and up‐regulated genes to plant hormone signal transduction, and metabolism of amino acids, terpenoids and polyketides (root cluster 4) (Figure 3c, d). The DEGs of each cluster were equally distributed between sub‐genomes (Figure S23). The DEGs in root from cluster 6 were involved in galactose and butanoate metabolism (Genes from the A and B sub‐genomes were inferred from the KEGG enrichment analysis.) (Figure S24). DEGs in shoot from cluster 5 were enriched in fatty acid degradation, and arginine and proline metabolism (Figure S24). These results provide evidence that the two sub‐genome of Cs play similar roles, or act in concert, in response to drought stress.

We also investigated drought‐responsive genes between each of *Setaria italica* (foxtail), *Sorghum bicolor* (sorghum) and *Oryza sativa* (rice), with Cs. We identified putative orthologs of 617 DEGs shared in all three pair‐wise comparisons (Table S16). A co‐expression network was constructed based on these conserved DEGs, along with water stress‐responsive lncRNAs and miRNAs (Figure S25 and Table S17). The results showed that lncRNAs, miRNAs and conserved DEGs (including TFs) constitute a complex transcriptional regulatory network under water stress and recovery. Evolutionarily conserved genes that were expanded (Table S18) were enriched in the categories fatty acid elongation, starch and sucrose metabolism, and phenylpropanoid biosynthesis (Figure S26). Phenylalanine ammonia‐lyase (PAL) is implicated in drought response (Liu *et al*., 2019). In Cs, we found the *PAL* family, consisting of 8 genes, was significantly expanded (Figure 3e). Among these, four *PAL* genes were differentially expressed in shoot upon drought treatment, and seven in root. 3‐ketoacyl‐CoA synthase (KCS), a key enzyme in fatty acid elongation (Kerstiens, 2006), is another of the conserved and expanded genes in Cs (Figure 3e). Starch and sucrose metabolism, and beta‐glucosidase (BGL) encoding genes, identified as conserved genes activated under water stress in Poaceae species, are also expanded in Cs. Furthermore, *glycoside hydrolase 17* (*GH17*), involved in degradation of sugar moieties from sugar polymers or other glycosylated molecules, was expanded in Cs. We posit that the expansion of conserved genes in key metabolic pathways may confer important functions in drought resistance in Cs.

Other significantly expanded gene families, equally distributed in the A and B sub‐genomes (Figure S27) include those encoding potassium transporters, receptor‐like kinases, and UDP‐glucoronosyl and UDP‐glucosyl transferases, which play defensive roles under both biotic and abiotic stresses (Ye *et al*., 2017), and may play similar roles in Cs (Table S19).

### Genetic determination of dimorphic flowers

**Figure 4:**
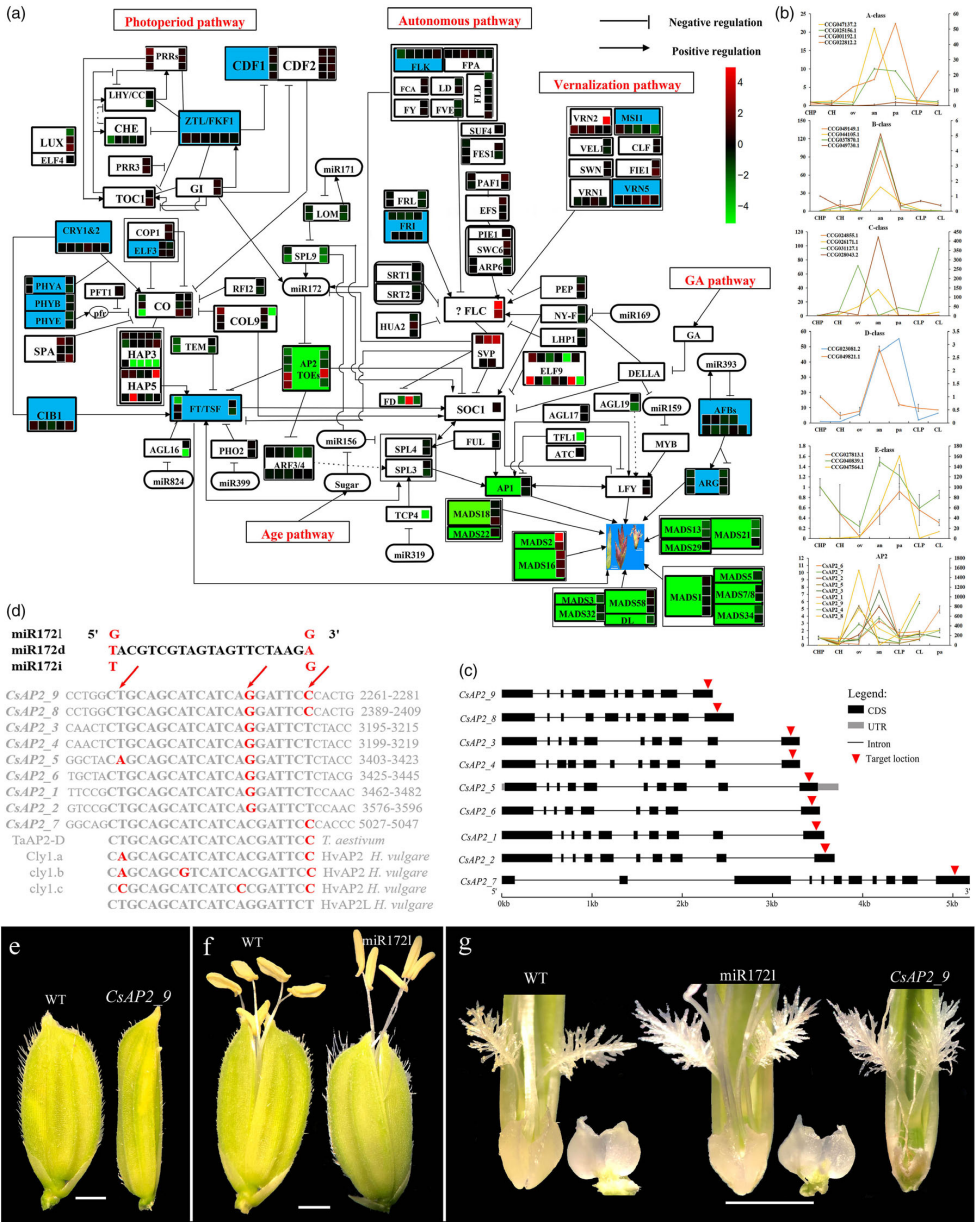
Genome‐wide identification of flowering genes. (a) Flowering gene network. Expanded gene families in blue rectangles, rectangles in green include A‐class (*AP1*, *AP2*, *MADS18* and *MADS22*), B‐class (*MADS2* and *MADS16*), C‐class (*MADS3*, *MADS32*, *MADS58* and *DL – DROOPING LEAF*), D‐class (*MADS13*, *MADS19* and *MADS21*) and E‐class genes (*MADS1*, *MADS5*, *MADS7/8* and *MADS34*). Gene expression levels shown in the colour schema (number of squares represent gene count, gene families in rectangles, miRNA in rounded rectangles, blue rectangles represent expanded gene families and green rectangles genes directly related flower development). (b) qRT‐PCR expression levels of AMGs. Right and left *y*‐axis is the relative expression level. CHP: CH flower primordium; ov: ovary; an: anther; pa: palea; CLP: CL flower primordium. (c) miR172 target locations in *CsAP2* genes. (d) Nucleotide variants in miR172 and its *AP2* TF target site sequences. Nucleotide variants are marked in red in bold. Nucleotide positions 15 and 21 (second and third red arrow) within the target site are variable compared to the *HvAP2* and Ta*AP2* target site. (e) *CsAP2_9* overexpression confers an abnormal palea in a spikelet in the T_0_ generation of transgenic plants. (f) miR172l overexpression confers five anthers with longer filaments in the T_0_ generation of transgenic plants, compared to six anthers in wild type. (g) Spikelets with dissected stigma and lodicules, showing degenerated lodicules in flowers in the T_0_ generation of *CsAP2_9* overexpressed transgenic plants. Scale bars represent 1 mm.

To gain insights into how dimorphic flowers evolved, we compared Cs flowering‐related genes with those in At and Os. Similar to the well‐developed At flowering gene network (Haider, 2014; Teotia and Tang, 2015), we grouped Cs flowering genes into five major pathways: vernalization, photoperiod, autonomous, gibberellin acid (GA) and age. Using 302 flowering genes, we built a gene network comprising the five pathways (Figure 4a, Table S20, Methods S2). Among the 83 flowering‐related gene families, 16 were expanded (>2x) compared to Os (Table S21), of which 10 were in the photoperiod pathway, including *CONSTANS*/*Flowering Locus T* (*CO*/*FT*) (Methods S2).

Compared to Os and At, most of the ABCDE model genes (AMGs) were expanded in Cs (Figure S28, Table S21). Based on RNA‐seq data, four A‐class Cs*MADs* showed higher expression in CL than in CH. Four B‐class, and seven C‐class genes exhibited flower‐specific expression (no expression in other tissues) (Table S22, Figure S29). Notably, two C‐class genes were expressed in different patterns in CH and CL flowers as shown in Figure S29. Among six D‐class genes, four exhibited flower‐specific expression, and two were expressed in all tissues. Among the E‐class genes, eight showed flower‐specific expression, with seven more abundant in CL. qRT‐PCR of AMGs showed that A‐class genes were highly expressed in the palea and anther, four B‐class genes were highly expressed in the anther (Figure 4b), C‐class genes were highly expressed in ovules or anthers, and D‐class and E‐class genes were highly expressed in the anther and palea (Figure 4b).

Gene expression is regulated by coordinated actions of transcription factors (TFs) and miRNAs (Hobert, 2008). From the transcripts specific to flowering, we identified 12 flowering‐related TF families (Table S23). From the 12 TF families, 168 genes had their expression correlated with AMGs by WGCNA (weighted gene co‐expression analysis; Figures S29 and S30). Only for members of the *MYB*, *SPL* and *NAC* families were their respective binding sites found in the promoter regions of 19 AMGs (Table S24). Among 35 Cs*SPLs,* 17 were located in the A sub‐genome and 18 in the B (Table S25). Phylogenetic analysis showed that Cs*SPLs* were grouped in eight clusters, with each cluster containing both Os*SPLs* and At*SPLs* (Figure S31). Of 16 Cs*SPLs* that are targeted by miR156s, all are expressed at higher levels in CH flowers (with the exception of Cs*SPL31*), which may contribute to the formation of larger CH flowers. Four Cs*SPL*s predicted to have binding affinity to 15 AMG promoter regions (Table S24, Figure S32) were targeted by miR156s. Only miR156p (with similar expression levels in CH and CL flowers) targeted Cs*SPL17* with better matches (Figure S33). However, the expression of miR156ab was inversely correlated with that of Cs*SPL17* in both CH and CL flowers, with miR156ab expressed more abundantly in CH (Table S23). Therefore, the interactions of miR156ab and Cs*SPL17* may be involved in dimorphic flower differentiation.

MYB transcription factors regulate multiple aspects of plant growth and development. We identified 266 *MYB* genes in the Cs genome, including 92 R1‐type, 165 R2R3, eight 3R and one 4R‐type (Table S26, Figure S34a). 45 *MYB* genes expressed in flowers were also differentially expressed (fold change >2) in shoots under drought stress (Table S26). Seven Cs*MYBs* are co‐expressed with *AMGs* based on WGCNA analysis (Figure S30). At*MYB21* is important for filament elongation under drought (Su *et al*., 2013). Phylogenetic analysis showed that eight Cs*MYBs* clustered together with At*MYB21*, At*MYB24* and At*MYB108* (Figure S34a). Of these, only Cs*MYB219*, an AMG TF, was highly expressed in CH flowers. Furthermore, Cs*MYB219* was significantly up‐regulated under water stress, and its expression level declined upon recovery. The amino acid sequence identity of Cs*MYB219* and At*MYB21* is 76.5%, with the both having conserved R2 and R3 domains (Figure S34b). We propose that Cs*MYB219* may be the functional homologue of At*MYB21* and that it performs a similar role in regulating Cs flower development under drought stress.

For another interactive pair, the miR159‐targeted Cs*MYB123*, the transcript level of miR159 and Cs*MYB123* was high and low, respectively in CL, but with the inverse pattern in CH (Table S23). Cs*MYB123* has a binding site in the promoter regions of four B‐class genes (Figure S33). B‐class genes also exhibited higher expression in CH than in CL (Table S22). Furthermore, qRT‐PCR results showed that transcript levels of the four B‐class genes were significantly higher in CH anthers than that in other tissues (Figure 4b). These results are consistent with a negative interaction between miR159 and Cs*MYB123* that leads to positive regulation of B‐class genes. Thus, the miR159 – Cs*MYB123* – B‐class gene module may be an important regulator of divergence in floral structure. Other interactive pairs acting in the same manner include miR164a/b with Cs*NAC34* (Figure S33).

The interaction of miR172s and *AP2* TF genes is known to promote floral opening in barley, as mutations in *cleistogamy 1* (*cly1*) cause failure in lodicule development (Nair *et al*., 2010; Ning *et al*., 2013). 32 Cs *AP2* genes (Cs*AP2*), with similarity to barley *Cly1* (Genbank, KF261344.1), were identified. Nine Cs*AP2* are putatively targeted by miR172s (Figure 4c). The target sites of five Cs*AP2*s are the same as those in Hv*AP2L* (*Cly1*), whilst Cs*AP2_7* is the same as Ta*AP2‐D*. Like their barley counterparts, Cs*AP2* genes are predicted to play a role in chasmogamous flower development (Figure 4d). miR172l is specifically targeted to two paralogs Cs*AP2_8* and Cs*AP2_9* (Figure 4d). At the gene expression level, transcripts of Cs*AP2*_*9* were more abundant in CL than in CH, with the Cs*AP2_8* showing inverse patterns. qRT‐PCR analysis validated the expression pattern of the nine Cs*AP2* genes (Figure 4b). These results support the hypothesis that Cs*AP2*_8 and Cs*AP2*_9, by interacting with miR172l, contribute to the regulation of cleistogamy in Cs.

For verification, we performed overexpression of Cs*AP2_9* and miR172l in transgenic rice, driven by the constitutive CaMV *35S* promoter. The effect of Cs*AP2*_9 and miR172l overexpression was examined in the spikelets of transgenic plants, relative to wild type, by PCR. Compared with wild type, transgenic plants also exhibited floral defects, with Cs*AP2_9* transgenic lines showing abnormal palea, and smaller and thinner lodicules; and miR172l lines showing longer filaments and reduced anther numbers (Figure 4e‐g). These results validate the functions of Cs*AP2_9* and miR172l in regulating lodicule, and filament and anther development, respectively.

## Discussion

A high‐quality chromosome‐level genome assembly and annotation has been achieved for *C. songorica* (Cs), using comprehensive sequencing technologies. Cs is an ecologically significant grass growing in saline and arid areas. This genome resource has important implications for improving our understanding of how plants adapt to, and thrive in water‐depleted environments. Being both agronomically, and genetically distant from rice, the Cs genome provides unique resources to study genotypic and phenotypic diversity in indigenous plants. Our initial efforts to select and cultivate this species have been successful (a lawn cultivar, *C. songorica* Roshev. cv 'Tenggeli' was released in 2016), and efforts to evaluate its adaptability to a broader range of environments are underway. For developing new forage cultivars, the availability of a high‐quality genome facilitates accurate characterization of new germplasm (as we have demonstrated), and an understanding of the genetics underlying important traits.

Polyploidization is very common in angiosperms (Gaeta *et al*., 2007) and has occurred multiple times over the course of the evolution of most flowering plants. Following polyploidization, deletions and rearrangements of duplicated genes occur before the genomes return to a diploid state (Bertioli *et al*., 2019). The sub‐genomes of allopolyploids are derived from different species, meaning that the two distinct genomes have undergone a series of genic, genomic and physiological accommodations to form the allopolyploid (Chen and Ni, 2006; Yang *et al*., 2019)*. C. songorica* (Cs) is a tetraploid, but its progenitor genomes remain unknown. The genome analysis here suggests a recent (within ~19.3 Mya) hybridization event occurred leading to tetraploidization. Recently formed allotetraploids typically exhibit gene retention with little genome reduction, such as that in white clover (Griffiths *et al*., 2019) and peanut (Bertioli *et al*., 2016; Bertioli *et al*., 2019). This may be also the case in Cs, as homeologous gene expression exhibits little or no bias between the sub‐genomes in different tissues and drought treatments. Polyploids are known to exhibit increased drought tolerance in several plant species (Chao *et al*., 2013; Zou *et al*., 2019). Cs is a xerophytic plant (Muvunyi *et al*., 2018), although a direct link between genome duplication and drought tolerance remain to be confirmed in this species.

The Cs genome provides new insights into the plant’s drought adaptability, and the differential development of dimorphic flowering. Cs can maintain high water content in leaf, and a largely normal rate of photosynthesis and stomatal conductance in dry soil (with 2% water content) (Yan *et al*., 2019b). It can survive and recover from prolonged periods of drought (Zhang *et al*., 2011), whilst nine weeks of *B. distachyon* seedlings showed above‐ground plant water content less than 35% after drought stress treatment with 25% soil water content (Martínez *et al*., 2018). At the molecular level, complex transcriptional regulatory networks have been revealed, with lncRNAs, miRNAs and conserved DEGs (including TFs) all involved. In the tetraploid Cs, no significant global expression dominance between A and B genomes was found. Some stress‐related gene families are conserved and expanded in Cs compared to other grass species. Drought tolerance is a systemic trait, with comprehensive metabolic re‐programming occurring upon stress challenges. The phenylpropanoid pathway is known to be involved in plant adaptability in harsh terrestrial environments (Ferrer *et al*., 2008). Phenylpropanoid‐based polymers contribute to the stability and robustness of plants towards drought or wounding (Vogt, 2010). PAL is the first step in the phenylpropanoid pathway. Compared with foxtail, sorghum and rice, the *PAL* family was significantly expanded in Cs. Functionally redundant PALs may provide robust regulation of the phenylpropanoid pathway under drought. Fatty acid elongation is the precursor pathway of the biosynthesis of cutin, suberin and wax. Cutin and suberin serve mainly as a waterproof barrier and are important for plant survival during extreme drought (Asaph *et al*., 2004; Kerstiens, 2006). The *KCS* gene family, involved in the biosynthesis of cutin, suberin and wax (Kerstiens, 2006), are conserved in Cs and expanded relative to foxtail, sorghum and rice.

Cleistogamy (CL) is more common in grasses than in other angiosperms and has been reported in over 320 species (Cheplick, 2007). CL flowering assures plant reproduction under variable environmental conditions, and its development is known to be affected by drought, chilling, salinity and light (Morinaga et al., 2008). The most common form of CL, as in Cs, is sheath fertilization, in which axillary inflorescences are enclosed within leaf sheaths, and the axillary spikelets grow along the stem axis (Cheplock, 1994). CL is subject to the complex control inputs of quantitative genetic loci, ontogeny and environment (Lloyd, 1984). A natural mutant of CL in barley has been characterized (Nair *et al*., 2010), providing an opportunity to understand the molecular basis of dimorphic flowering. CH and CL flowers in *Viola philippica* are influenced by photoperiod, and the expression level of several *MADS* genes upon floral induction (Li *et al*., 2016). In Cs, we showed that a complex of regulators including miRNA, *SPL* and *MADS* genes may function as a module in the differential development of CH and CL flowers. miR156 and *SPL* are known to interact in regulating ovary development (Xing *et al*., 2010; Silva *et al*., 2014) and that expression of MADS box genes is repressed in developing ovaries upon miR156 overexpression (Schwab *et al*., 2005; Wu *et al*., 2009). In Cs, the expression levels of miR156 and Cs*SPL* are complementary in CL flowers. MYB family members are also involved in stamen development (Cheng *et al*., 2009). We observed that the contrasting expression of miR159 and Cs*MYB123* leads to increased expression of B‐class genes. Thus, the miR159 – Cs*MYB123* – B‐class gene module may be an important regulator of floral stamen divergence.

In Arabidopsis, overexpression of At*MYB21* was able to restore stamen filament elongation (Cheng *et al*., 2009), and At*MYB21* is required for filament elongation under drought (Su *et al*., 2013). Cs*MYB219*, a TF of the AMG and the putative ortholog of At*MYB21*, has divergent expression levels in CH and CL flowers. The expression of Cs*MYB219* is significantly up‐regulated under water stress and down‐regulated upon recovery following watering. The stategy of dimorphic flowering may have evolved as a long‐term adaptation to water depleted environments. In barley, a mutation in the *Cly1* gene leads to abnormal development of the lodicules and closed pollination (Nair *et al*., 2010). In Cs, the organs of CH and CL flowers are structurally distinct, and the lodicules of CL flowers are atrophied. From experiments in transgenic rice, abnormal lodicules were observed when Cs*AP2_9* was overexpressed, and longer filaments when miR172l was overexpressed, strongly implicating these genes in the regulation of dimorphic flowering.

Screening for variants of Cs genes in germplasm collections may identify new sources of drought tolerance for crop improvement. For example, overexpression of Cs*ALDH* and Cs*LEA* in alfalfa conferred enhanced tolerance to drought stress (Duan *et al*., 2015; Zhang *et al*., 2016), thereby improving its ability to maintain yields under limiting water supply. Similarly, the genome of Cs provides opportunities to identify and characterize key genes controlling the differentiation of chasmogamy and cleistogamy. Genome‐wide gene characterizations in Cs will enable us to conduct systems genetic modelling of drought tolerance and ultimately to delineate the interplay and co‐evolution of dimorphic flower formation and drought tolerance in this species.

## Methods

### Plant materials and DNA sequencing

Genomic DNA was isolated from leaves of a *Cleistogenes songorica* Roshev. cv 'Tenggeli' plant using a modified SDS method (Möller *et al*., 1992). A KAPA library preparation kit was used to prepare Illumina sequencing libraries. Four libraries with insert size of 200 bp (paired‐end), 450 bp (paired‐end), 1000 bp (mate‐paired) and 2000 bp (mate‐paired) were sequenced on Illumina HiSeq 2000. Library with insert size of 40 kb was constructed and sequenced using PacBio Sequel Ⅱ system. One SMRT CLR cell yields ~172 Gb data (mean read length ≥18 kb, max read length >251 kb). Raw reads were filtered with sequencing quality >Q30, with adaptors and duplicated reads removed.

### Genome size estimation

Standard flow cytometer and K‐mer counting method were used to estimate genome size. *C. songorica* seeds were grown on filter paper under a 16‐h light/8‐h dark at 25 °C, in an incubator. Root tips were excised and treated using routine methods (Yang *et al*., 2017) for chromosome counting. Flow cytometry (Dolezel and Bartos, 2005; Tao *et al*., 2018) was used to estimate genome size. The clean reads (61.98 Gb) from the Illumina library were used to estimate the genome size using k‐mer analysis by Jellyfish (Marçais and Kingsford, 2011). Formula of G = k‐mer_number/k‐mer_depth was used to estimate genome size. A total of 21 690 035 273 17‐mers were generated and the depth of 17‐mer peak was 40, the genome size of *C. songorica* was estimated to be ~541.95 Mb (Table S1).

### Genome assembly and quality assessment

The assembly of *C. songorica* genome was performed using PacBio reads and Illumina reads. De novo assembly of the PacBio long reads was conducted using Falcon v 0.3.0. (https://github.com/PacificBiosciences/FALCON/) (Chin *et al*., 2016). FALCON pipeline was used to correct error and pre‐assembly. Parameters of FALCON were compared and optimized during the pre‐assembly. Based on the contig N50 of pre‐assembly, we used the following parameters: length_cutoff = 35 000 and length_cutoff_pr = 34 000 to construct initial contigs. The contigs were polished using Arrow (https://github.com/PacificBiosciences/GenomicConsensus). The PacBio long reads were mapped to the assembled contigs with the blasr pipeline (Chaisson and Tesler, 2012). After the Arrow correction, all the filtered Illumina reads were mapped to the corrected contigs with BWA‐mem (Li and Durbin, 2009) and further corrected using Pilon (Walker *et al*., 2014) by running 3 times.

We assessed the completeness, coverage and accuracy of the final assembly using conserved genes and RNA‐seq data. The completeness of the genome assembly was assessed by BUSCO (http://busco.ezlab.org/) (Simão *et al*., 2015) using 1375 single‐copy orthologous genes. RNA‐seq data collected from inflorescences of both CH and CL flowers, and leaf and root tissues under drought treatments (Zhang *et al*., 2011) were deployed and mapped to the assembled contigs. A total of 69 331 unigenes were also mapped to the assembled contigs.

### Pseudochromosome construction and validation using three‐dimensional proximity information (Hi‐C)

Fresh leaf tissues were fixed in formaldehyde to maintain nuclear DNA location. DNA was extracted using the SDS method. Cross‐linked DNA was digested with *Hin*d III, the sticky ends were biotinylated, diluted and ligated to each other randomly. The interaction fragments were cyclized to link the interaction locations during the sequencing and analysis. The cross‐linked DNA was recovered and purified into 300–700 bp segments. The interaction DNA fragments were captured, and a sequencing library was constructed. Qubit 2.0 and Agilent 2100 were used to check the concentration and insert fragment size. QPCR was used to confirm the concentration to ensure the library quality. The library was made to paired‐end sequencing on a HiSeq X Ten platform, with read length of 150 bp.

Hi‐C (Burton *et al*., 2013; Flot *et al*., 2015; Noam and Job, 2013) was used to evaluate and validate genome assemblies, enabling construction of pseudochromosomes. After the removal of adaptors, reads were aligned against the assembled contigs by BWA‐mem (Li, 2013) in a 2‐step protocol to avoid chimeric reads. Only the valid interaction pairs (43 154 161 read pairs) were used for the interaction map construction. The assembled contigs were divided into equally sized bins (250 kb) to group pseudochromosome clusters using LACHESIS (Burton *et al*., 2013), with the parameters: CLUSTER_MIN_RE_SITES = 300, CLUSTER_MAX_LINK_DENSITY = 3, CLUSTER_NONINFORMATIVE_RATIO = 2.4, ORDER_MIN_N_RES_IN_TRUNK = 100, and ORDER_MIN_N_RES_IN_SHREDS = 100. HiCPlotter software (Akdemir and Chin, 2015) was used to plot the contact maps.

Genome duplication analysis was based on homologous proteins using BLAST with *e*‐value < 1e‐5. Collinear blocks were analysed by using MCScanX (Wang *et al*., 2012) with default parameters, with each block required to have at least five collinear gene pairs. The rate of Ks was calculated for each gene pair identified in *C. songorica* using the PAML yn00 NG model (http://abacus.gene.ucl.ac.uk/software/paml.html). The date of the WGD event was calculated as Ks/2λ, where λ is the mutation rate (6.5e‐9) which was estimated for *O. sativa* (Han and Zhu, 2011).

### Repetitive sequence analysis

Both *de novo* and homology‐based strategies were used to annotate the genome repetitive sequences. *De novo* prediction software included LTR‐FINDER (Zhao and Hao, 2007) and RepeatModeler (http://repeatmasker.org/RepeatModeler.html). The repeat libraries were combined and merged in RepBase to generate a repetitive sequence database. All identified repeats were classified into repeat families by the PASTEClassifier (https://urgi.versailles.inra.fr/Tools/PASTEClassifier) with REPET. Homology‐based repeat search was conducted through RepeatMasker (http://www.repeatmasker.org). In addition, we used RepeatProteinMask implemented in RepeatMasker, together with the WU‐BLASTX to identify any repeat‐related proteins missed in the previous steps.

### Gene and non‐coding RNA prediction

Non‐redundant and high‐confidence gene sets were obtained using Glean software (Elsik *et al*., 2007), which integrates information from homologues using geMoMa (Keilwagen *et al*., 2016), *De novo* (Augustus (Mario *et al*., 2006) was used to predict unigenes, with transcriptome data obtained from leaf and root tissues), and transcripts. Gene functional annotations were based on homologous alignment with BLAST (*e*‐value < 1 × 10^−5^) against well‐curated databases including Nt, Nr, KEGG, SwissProt and TrEMBL (Birney *et al*., 2004; Kent, 2002; Majoros *et al*., 2004; Stanke *et al*., 2006; Trapnell *et al*., 2010). InterProScan (http://www.ebi.ac.uk/Tools/pfa/iprscan/) was used to predict protein motifs and domains, and to assign GO‐terms to the annotated genes.

tRNAs were identified using tRNAscan‐SEM (Lowe and Eddy, 1997). For rRNA identification, *O. thomaeum*’s rRNA sequences (https://phytozome.jgi.doe.gov/pz/portal.html) were used as reference sequences for blastn search. miRNA and snRNA were predicted using INFERNAL (http://eddylab.org/infernal/) based on the Rfam database (Griffithsjones *et al*., 2005).

### Comparative gene family analysis

The assembled and annotated genome was compared with that of nine plant genomes of *A. thaliana, B. distachyon, O. thomaeum, O. sativa, Sorghum. bicolor, Setaria. italica,* (https://phytozome.jgi.doe.gov/pz/portal.html), *E. tef* (https://genomevolution.org/coge/); *H. vulgare* (http://webblast.ipk-gatersleben.de/barley_ibsc/downloads/) and *Z. mays* (https://www.ncbi.nlm.nih.gov/genome). Based on the 54 383 predicted genes of *C. songorica* and the protein sets of the nine species, gene family clustering was conducted using OrthoMCL (Li *et al*., 2003). Gene family expansion and contraction in the sequenced genomes were estimated using CAFE3 (Han *et al*., 2013). Phylogenetic trees were built based on single‐copy genes from *C. songorica* and the nine species (with *A. thaliana* as outgroup). Single‐copy genes were aligned using MUSCLE (Edgar, 2004), and phylogenetic trees were built using PhyML3.0 (http://www.atgc-montpellier.fr/phyml/), with maximum likelihood method, Jones‐Taylor‐Thornton model and 1000 bootstraps. Divergence time of each species was estimated using MCMCtree in the PAML package based on relaxed normal molecular clocks, with calibration set to 148–173 Mya between monocotyledon and true dicotyledon (Kumar *et al*., 2017). Fourfold degenerate synonymous sites of each single‐copy gene family were used to estimate molecular clock (replacement rate) and divergence time among species. Evolutionary rate of a neutral gene was measured by variable sites number of each year of each site. The PAML yn00 model (http://abacus.gene.ucl.ac.uk/software/paml.html) was used to calculate the Ks value of *C. songorica* paralogs.

### Comparative gene analysis and sub‐genome identification

Homologous genes between *C. songorica* (Cs) and *Z. mays* (Zm), *S. bicolor* (Sb), *O. thomaeum* (Ot) and *O. sativa* (Os) were aligned using BLAST (*E*‐value < 1e‐10). Cs genes were also aligned to each other to identify paralogs within the genome. MCScanX (Wang *et al*., 2012), with default parameters, was used to construct genomic synteny between Cs – Zm, Cs *–* Sb, Cs *‐* Cs, Cs ‐ Os and Cs – Ot. Genome synteny was visualized using Circos (ver 0.69) (Krzywinski and Schein, 2009). Sub‐genomes of Cs were identified partly based on the synteny relationships between Os and Cs chromosomes. Cs chromosomal rearrangements were detected based on the synteny of the constructed chromosomes of Cs.

### *Cleistogenes* accessions and molecular marker development

Twenty‐three accessions of *Cleistogenes spp*. were collected from various regions in China (Table S27). Primers of genome‐wide LTR retrotransposon, miRNA and intron‐length polymorphic (ILP) were designed using DNAMAN software. The amplified fragments were scored independently as 1 and 0 for presence and absence in samples and then used for statistical analysis. Data analyses were completed using NTSYS‐pc version 2.10 software and R packages. Principal components analysis (PCA) was used to investigate the overall population structures among accessions (Oksanen and Minchin, 1997), which was performed with the ‘vegan’ package, and then plotted with ggplot2 package in R 3.5.2.

### Plant growth and sampling for transcript analysis

Seeds were harvested from Cs plants sown in Minqin County, Gansu Province, China. Bleach‐sterilized seeds of Cs were germinated and grown in a glasshouse under controlled conditions, with growth mixture of sand/ vermiculite (1:1, v/v), at temperatures of 28/ 24°C (day/night), irradiance of 150 μmol quanta m^–2^ s^–1^, 16‐h light and 8‐h dark cycles, and 65% relative humidity. Four‐week‐old plants were transplanted into individual pots with the same growth medium. Each pot (0.45 kg) was irrigated with 100 mL Hoagland nutrient solution every three days.

Total RNA was isolated using the TRIzol reagent (Invitrogen) following the manufacturer's instructions. For samples with salt, cold, heat and ABA treatments, messenger RNA (mRNA) were separated from the total RNA by Oligo (dT) and cleaved into short random fragments. For samples under drought stress, ribosomal RNA was removed by Epicentre Ribo‐zero™ rRNA Removal Kit (Epicentre, USA), and rRNA‐free residues were cleaned up by ethanol precipitation. Sequencing libraries were generated using the rRNA depleted RNA by NEBNext® Ultra™ Directional RNA Library Prep Kit from Illumina® (NEB, USA) following manufacturer’s recommendations. Quality cDNA libraries were constructed by PCR enrichment and sequenced in paired‐end on a HiSeq2500 with read length of 125 bp (Yan *et al*., 2019a; Yan *et al*., 2019b). For flower samples, cDNA library construction and sequencing were based on the Illumina HiSeq 2000 platform (San Diego, CA, USA). Small RNAs of flowers were sequenced on the Illumina HiSeq 2000 platform (Wu *et al*., 2018).

Clean reads were obtained by removing adapters, reads containing poly‐N and lower quality reads (<Q30). Clean RNA‐seq reads were mapped to the Cs genome using HISAT2 (Daehwan *et al*., 2015). StringTie (1.3.1) was used to calculate FPKMs of both lncRNAs and mRNA in each sample (Mihaela *et al*., 2015). FPKM of genes were computed by summing the FPKMs of transcripts in each gene group. Differential expression analysis was performed using the DESeq R package (v1.10.1, negative binomial distribution). FDR (false discovery rate) were adjusted using the PPDE (posterior probability of being DE), FDR < 0.01 and |log2 (FoldChange)| ≥2 set as the threshold for significantly differential expression.

KOBAS software was used for testing the statistical enrichment of lncRNAs targeted genes with reference to KEGG pathways (Mao *et al*., 2005). K‐cluster analysis of DEGs was performed using the OmicShare tools (http://www.omicshare.com/tools). For water stress‐related DEGs, a common set among Cs, foxtail millet, sorghum and rice were identified using OrthoMCL with default settings (Dugas *et al*., 2011; Li *et al*., 2003; Qi *et al*., 2013; Zhang *et al*., 2012). Target genes of miRNA and lncRNA were collected based on our previous study (Yan *et al*., 2019b).

### Homologous gene pair expression and KEGG enrichment analysis

Homologous gene pairs between A and B sub‐genomes were identified using MCScan (Wang *et al*., 2012) with default parameters. Gene expression levels were quantified using log_10_ (FPKM). The syntenic gene pairs between the A and B sub‐genomes (CsA and CsB) were used for homologous expression dominance analysis. Syntenic gene pairs with |CsA/ CsB| ≥2 or ≤0.5 were defined as dominance gene pairs, and the dominant and subordinate genes assigned. Syntenic gene pairs with non‐dominance were classified as neutral genes. To test whether the occurrences of dominant gene pairs from A and B are equal, we performed double‐side binomial tests (Schnable *et al*., 2011).

For all 26 treatments (Methods S1), all dominant genes from A or B were subjected to KEGG gene enrichment analysis. If a gene was CsA dominant under one stress condition and CsB dominant gene under another stress condition, it was discarded. All dominant genes with higher expression (greater than two‐fold change), or specific to stress conditions compared with the control, were subjected to KEGG enrichment analysis, as were all neutral genes with higher expression (greater than two‐fold change), or specific to stress conditions compared with the control conditions.

### Co‐expression network analysis of flowering genes

*C. songorica* flowering genes were grouped into five major pathways, that is vernalization, photoperiod, autonomous, gibberellin acid (GA) and the age pathway, based on the *A. thaliana* flowering gene network (Haider, 2014; Teotia and Tang, 2015). The detailed description of member genes from each pathway is provided in Table S23. Orthologs in *C. songorica* were identified using blastn (*e*‐value < 1e‐5) and blastp (*e*‐value < 1e‐10). Phylogenetic trees were constructed using MEGA7.0. with Neighbour‐Joining method with default substitution models and a bootstrap value of 1000 replicates.

The co‐expression network was constructed based on KEGG pathways, with network modules representing flowering pathways (Ogata *et al*., 2009), and such a network was used to identify co‐expressed modules (Soichi *et al*., 2010) and transcription factors (Childs *et al*., 2011). All transcripts from flower, shoot, leaf and root were used for WGCNA analysis. Candidate flowering‐related transcription factors involved in flower organ development were predicted based on PlantTFDB (http://planttfdb.cbi.pku.edu.cn/index.php) and WGCNA. PlantPAN2.0 (http://plantpan2.itps.ncku.edu.tw/index.html) was applied to predict binding sites of transcription factors, with matched binding sites assigned if similarity >90%. miRNAs were identified through Blast against the miRBase 19.0 (http://www.mirbase.org/). Target sites of miRNA were predicted using PsRobot (http://omicslab.genetics.ac.cn/psRobot/). Constructed co‐expression networks were visualized using cytoscape3.5.1.

### *C. songorica* ABCDE model genes

The ABCDE model‐related DNA and protein sequences of Os and At were downloaded from RiceData (http://www.ricedata.cn/gene/) and TAIR (https://www.arabidopsis.org/index.jsp), respectively. These sequences were compared to Cs nucleotide and protein datasets (*e*‐value < 1 × e^−100^) to find orthologs in Cs. Cs gene symbols were kept the same as Arabidopsis. We performed phylogenetic analyses including ABCDE model proteins from Cs, At and Os. Gene structure of the identified ABCDE model genes was plotted using online Gene Structure Display Server (http://gsds.cbi.pku.edu.cn/).

### Gene family identification in *C. songorica* genome

All At and Os gene family member sequences were downloaded from Phytozome v12.1 (https://phytozome.jgi.doe.gov/pz/portal.html#). Blast‐2.6.0 + was performed to identify the corresponding gene family members in Cs with a cut‐off *e*‐value < 10^−5^. All identified sequences, with redundant sequences removed, were submitted to Pfam (http://pfam.xfam.org/search/keyword?query=&submit=Submit#tabview=tab1) for annotations. Valid sequences from At, Os and Cs were submitted to the online tool MEME (http://meme-suite.org/tools/meme) to identify conserved motifs.

### Real‐time quantitative RT‐PCR

*C. songorica* fresh lemma, palea, anther, pistil and flower primordium of CH flower and CL flower primordium were separation under dissecting microscope (SZ2‐ILST, Olympus Corporation, Tokyo Japan) and stored in RNA‐Be‐Locker A reagent (Sangon Biotech, Shanghai, China) which permeates tissues, stabilizes and protects RNA expression pattern and prevents RNA degrading. CH and CL flowers were collected and stored in RNA‐Be‐Locker A reagent. CL floral organ is too small to get undegraded RNA tissues under a dissecting microscope, so CL flower organ samples were not obtained. mRNA was extracted from the above tissues for qRT‐PCR using RNAiso regent (TaKaRa, Dalian, China). Reverse transcription was performed according to the manufacturer’s instructions of PrimeScript® RT reagent Kit (TaKaRa). The expression of some AMGs were quantified on Applied Biosystems 7500 Real‐Time PCR System using 2xSG Fast qPCR Master Mix (Low Rox) kit using mRNA‐specific primers (designed using Perlprimer software) (Table S21). Normalization was performed relatively to *CsGAPDH*, and the data were collected from three technical replicates per sample.

### Gene transformation in rice

Mature seeds of the japonica rice cultivar ‘Nipponbare’ were used in this study. For generating *CsAP2_9* and miR172l overexpression lines, the coding sequence of *CsAP2_9* and precursor sequence of miR172l were amplified by PCR and then cloned into the expression vector pART‐CAM using XhoI/ XbaI sites. The construct was transformed into rice by *Agrobacterium tumefaciens*‐mediated transformation method (Hiei and Komari). Flowers in the T_0_ generation of transgenic plants were used for analysis.

## Authors’ contributions

J.Z., Y.W., G.S. and Z.N. designed the project; J.Z., Y.W., F.W., Y.Z. and Q.Y. collected the experimental materials, prepared and purified the DNA and RNA samples; F.W., Q.Y., U.J., P.X., Z.Z. and J.L. performed transcriptome and genetic analyses and identified candidate genes of flowering; F.W., Q.Y., T.M., R.L., Y.Z., G.K., Y.L. and R.L. worked on flower phenotype and identified stress‐related genes. F.W., Q.Y., J.Z. and M.C. wrote the manuscript, and J.Z., M.C., U.J., G.S. and Y.W. revised the manuscript.

## Conflicts of interest

The authors declare no competing interests.

## Supporting information


**Material S1** Transcriptome analysis.
**Figure S1** Chromosome counts (2n = 40) in a *C. songorica* cell.
**Figure S2** Cytograms of forward scatter (logarithmic scale, FS log) vs. side scatter (logarithmic scale, SS log).
**Figure S3** K‐mer frequency distribution.
**Figure S4** Inter‐chromosomal contact matrix.
**Figure S5** GC distribution of the genome assembly with 2nd (Illumina) and 3nd (PacBio) combined.
**Figure S6** Comparisons of gene structural parameters (the number and length of exons, intron length) among the seven grass species.
**Figure S7** TEs (DNA transposons, retrotransposons LINE, LTR and SINE) sequence divergence.
**Figure S8** Genomic landscape of the 20 assembled *C. songorica* chromosomes.
**Figure S9** Matrix of dot plots of paralogues on the chromosomes of *C. songorica* showing reciprocal intra‐genomic chromosomal rearrangements.
**Figure S10** Phylogenic tree of *C. songorica* pseudochromosomes and *O. thomaeum* scaffolds based on single‐copy orthologs.
**Figure S11** Distribution of Ks values.
**Figure S12** Syntenic relationships of Cs ‐ Os and Cs ‐ Ot.
**Figure S13** Typical micro‐ synteny patterns between genomic regions from *C. songorica* (Cs) and *O. thomaeum* (Ot), *C. songorica* and *O. sativa* (Os).
**Figure S14** Locations of LTR, ILP and miRNA makers on *C. songorica* chromosomes.
**Figure S15** Dendrogram of 23 *Cleistogenes* accessions based on UPGMA Cluster analysis using 1119 polymorphic SSR makers.
**Figure S16** Venn plot of gene families in the five species.
**Figure S17** KEGG enrichment of *C. songorica* genes (a) KEGG enrichment of *C. songorica* unique genes.
**Figure S18** *C. songorica* PEPC gene analysis.
**Figure S19** PEPC genes expression.
**Figure S20** PEPC genes collinearity and location on the chromosomes.
**Figure S21** Homologous expression dominance in *C. songorica*.
**Figure S22** Density of dominant genes, neutral genes and TEs in A and B sub‐genome.
**Figure S23** Differentially expressed genes (DEGs) analysis.
**Figure S24** KEGG analysis of DEGs in *C. songorica* sub‐genomes.
**Figure S25** Representatives of predicted interaction networks among lncRNAs, Poaceae conserved genes and miRNAs.
**Figure S26** Comparisons of drought‐responsive genes across *C. songorica*, rice, foxtail, and sorghum.
**Figure S27** Distribution of significantly expanded genes in A and B sub‐genomes.
**Figure S28** Phylogenetic analysis and genes structure of ABCDE model gene.
**Figure S29** WGCNA analysis of tissue‐specific genes.
**Figure S30** ABCDE model genes, TFs and miRNA co‐expression network.
**Figure S31** Phylogeny and expression of SPL gene family members in *C. songorica*.
**Figure S32** SBP, MYB and NAC transcription factor binding sites (TFBSs) in AMGs’ promoter regions.
**Figure S33** miRNA target sites in TFs gene in *C. songorica*.
**Figure S34** MYB gene family of Arabidopsis, rice, and *C. songorica*.

**Material S2** Identification of flower development‐related genes.
**Table S1** Results of 17‐mer statistics.
**Table S2** Statistics of PacBio long reads assembly.
**Table S3** Assembly statistics of the *C. songorica* genome.
**Table S4** Coverage rate and mapping rate of Illumina reads aligned to assembly genome.
**Table S5** BUSCO analysis of *C. songorica* genome assembly in embryophyta.
**Table S6a** Flower gene sets coverage rate in the *C. songorica* genome.
**Table S6b** Leaves under drought tolerance gene sets coverage rate.
**Table S7** Coverage of gene function annotations from different sources.
**Table S8** Statistics of the predicted genes.
**Table S9** Statistics of the annotated RNAs.
**Table S10** Statistics of the repetitive elements.
**Table S11** Transposon elements prediction and statistics.
**Table S12** Correspondences of chromosome ID of the genome assembly and assigned sub‐genomes.
**Table S15** Nonsynonymous substitutions (Ka) and synonymous substitutions (Ks) values of PEPC homologous genes.
**Table S27** *Cleistogenes* accessions used for molecular marker analysis.

**Table S13** KEGG enrichment of homologues genes, with the ration of 4:1 or 3:1 (*C. songorica*: *O. sativa*) and (*C. songorica*: *O. thomaeum*).

**Table S14** KEGG enrichment of the *C. songorica* unique genes.

**Table S16** List of conserved drought‐responsive genes shared across 4 Poaceae species and their expression patterns in *C. songorica*.

**Table S17** Representatives of the predicted interaction networks among lncRNAs, Poaceae conserved genes and miRNAs.

**Table S18** Gene list of conserved genes and expanded genes.

**Table S19** Significantly expanded genes.

**Table S20** Flowering‐related genes information.

**Table S21** Homologous genes between *C. songorica* and *O. sativa*.

**Table S22** ABCDE‐MADS genes expression.

**Table S23** Transcription factors of ABCDE‐MADS and miRNA expression.

**Table S24** TFs binding site in AMGs promoter region.

**Table S25** Expression levels of CsSPLs in different tissues.

**Table S26** Expression of MYB family members.

**Data S1** Single‐copy genes from *C. songorica* and other nine species.

**Data S2** Gene IDs of the copy number ratio between *O. sativa* and *C. songorica* = 1:3, 1:4 and 1:5.

**Data S3** Gene families identified using OrthoMCL in ten species.

## Data Availability

The *C. songorica* genome sequencing data (including Illumina, PacBio and Hi‐C raw data) have been deposited in NCBI, under accession number PRJNA634005. Seeds, leaf, salt treatment, ABA treatment of *C. songorica* RNA‐seq raw data have been deposited under SRA accession numbers of PRJNA634405 and PRJNA634406. The assembled genome sequences and genome annotations have been deposited in the National Genomics Data Center (https://bigd.big.ac.cn/?lang=en), under accession PRJCA002752.
