# Supplementary material for: The genome of Cleistogenes songorica provides a blueprint for functional dissection of dimorphic flower differentiation and drought adaptability
Source: Plant Biotechnol J. 2020 Oct 28;19(3):532–47. doi: 10.1111/pbi.13483 (PMC7955882; doi:10.1111/pbi.13483)
Supplement: Supplementary file 1 — Material S1 Transcriptome analysis. Figure S1 Chromosome counts (2n = 40) in a C. songorica cell. Figure S2 Cytograms of forward scatter (logarithmic scale, FS log) vs. side scatter (logarithmic scale, SS log). Figure S3 K‐mer frequency distribution. Figure S4 Inter‐chromosomal contact matrix. Figure S5 GC distribution of the genome assembly with 2nd (Illumina) and 3nd (PacBio) combined. Figure S6 Comparisons of gene structural parameters (the number and length of exons, intron length) among the seven grass species. Figure S7 TEs (DNA transposons, retrotransposons LINE, LTR and SINE) sequence divergence. Figure S8 Genomic landscape of the 20 assembled C. songorica chromosomes. Figure S9 Matrix of dot plots of paralogues on the chromosomes of C. songorica showing reciprocal intra‐genomic chromosomal rearrangements. Figure S10 Phylogenic tree of C. songorica pseudochromosomes and O. thomaeum scaffolds based on single‐copy orthologs. Figure S11 Distribution of Ks values. Figure S12 Syntenic relationships of Cs ‐ Os and Cs ‐ Ot. Figure S13 Typical micro‐ synteny patterns between genomic regions from C. songorica (Cs) and O. thomaeum (Ot), C. songorica and O. sativa (Os). Figure S14 Locations of LTR, ILP and miRNA makers on C. songorica chromosomes. Figure S15 Dendrogram of 23 Cleistogenes accessions based on UPGMA Cluster analysis using 1119 polymorphic SSR makers. Figure S16 Venn plot of gene families in the five species. Figure S17 KEGG enrichment of C. songorica genes (a) KEGG enrichment of C. songorica unique genes. Figure S18 C. songorica PEPC gene analysis. Figure S19 PEPC genes expression. Figure S20 PEPC genes collinearity and location on the chromosomes. Figure S21 Homologous expression dominance in C. songorica. Figure S22 Density of dominant genes, neutral genes and TEs in A and B sub‐genome. Figure S23 Differentially expressed genes (DEGs) analysis. Figure S24 KEGG analysis of DEGs in C. songorica sub‐genomes. Figure S25 Representatives of predicted interact [file PBI-19-532-s014.docx]

**The genome of *Cleistogenes songorica* provides a blueprint for functional dissection of dimorphic flower differentiation and drought adaptability**

Supporting information

**Supporting** figures


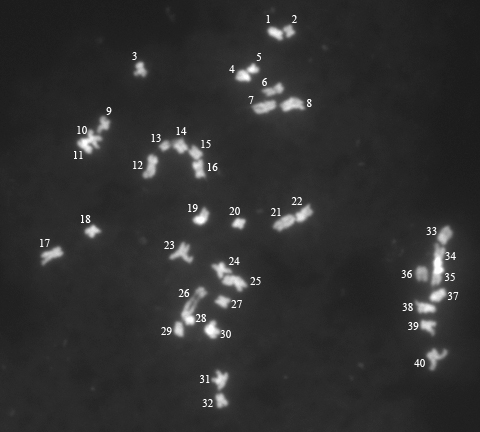


Figure S1 Chromosome counts (2n = 40) in a *C. songorica* cell.


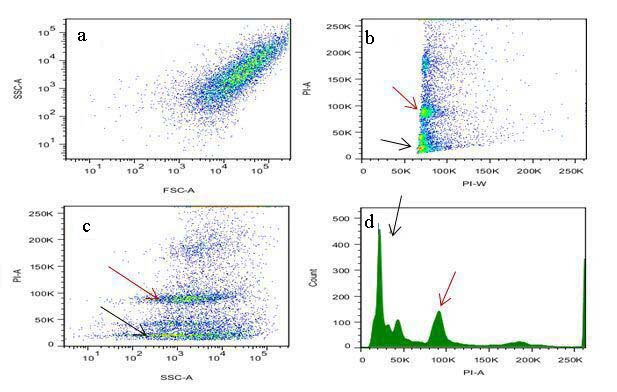


Figure S2 Cytograms of forward scatter (logarithmic scale, FS log) vs. side scatter (logarithmic scale, SS log). (a) PI fluorescence width vs PI fluorescence height. (b) Cytograms of SS log vs. PI fluorescence of nuclear suspensions. (c) PI fluorescence intensity (PI fluorescence, channel numbers). (d) Estimates *C. songorica* genome size. Black arrow shows the target species (*C. songorica*) while red arrow shows the internal standard (Maize).


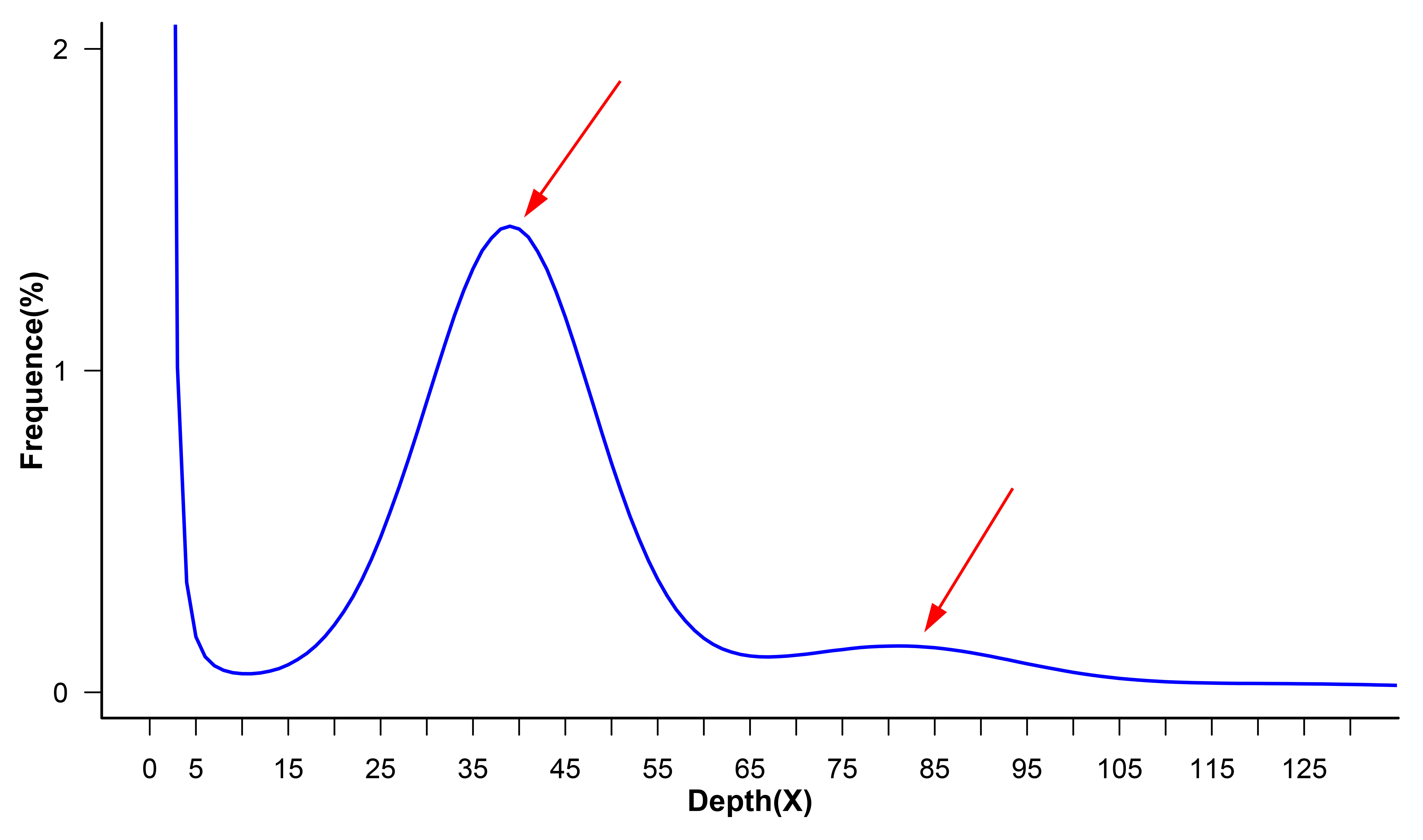


Figure S**3** K-mer frequency distribution. The sharp peak depth was 40 in the distribution. There is one sharp peak (around 40) and a very smooth peak (around 80) in k-mer frequency distribution curve (Kajitani et al., 2014). The sharp peak in k-mer distribution is expected from a homozygous region of the genome(Kajitani et al., 2014; Yasui et al., 2016). The smooth peak around the double position of sharp peak suggest the allotetraploidy of *C. songorica* (Jarvis et al., 2017; Yasui et al., 2016).


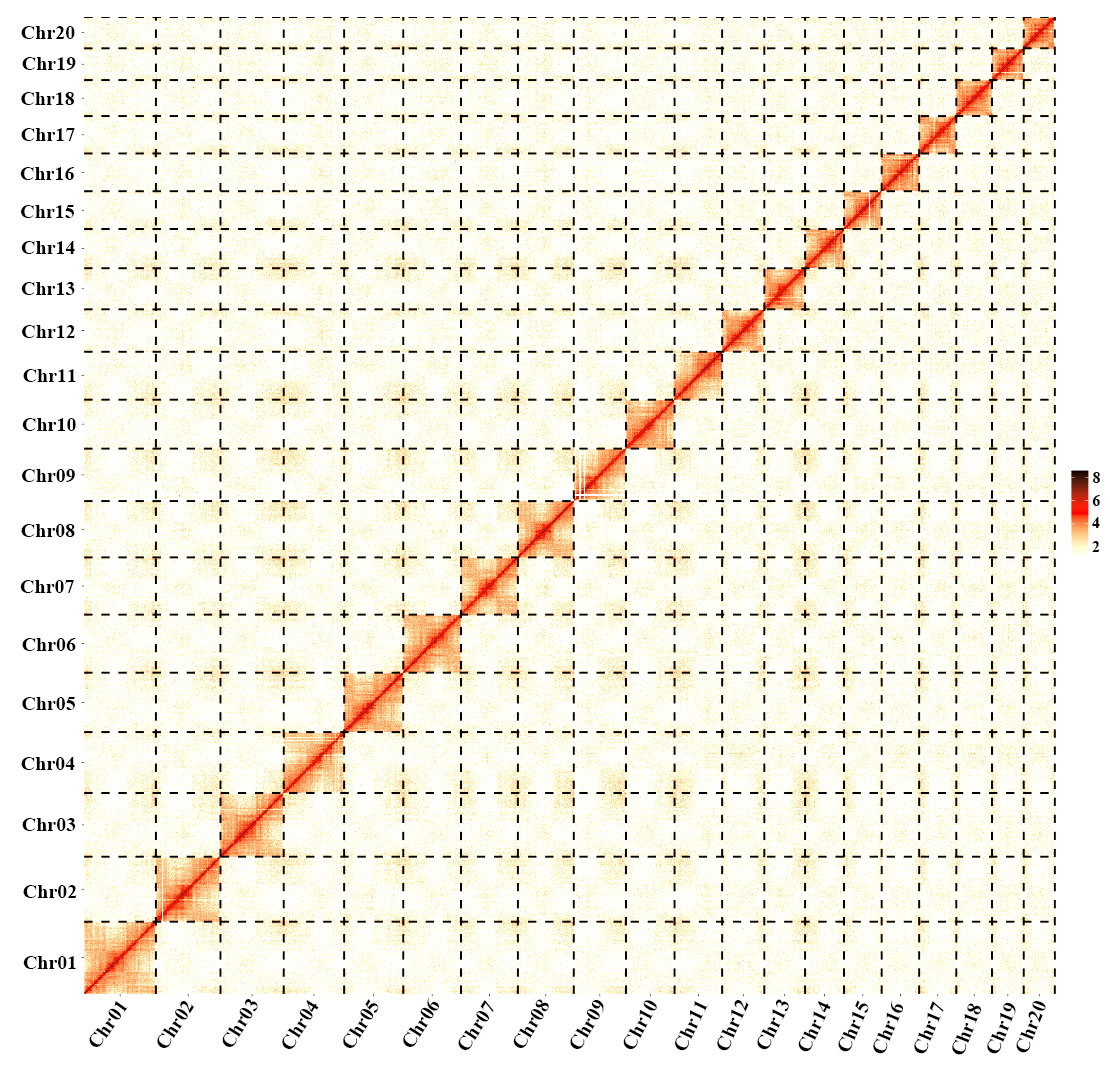


Figure S4 Inter-chromosomal contact matrix. The intensity of pixels represents the normalized count of Hi-C links between 250 kb windows on 20 chromosomes on a logarithmic scale.


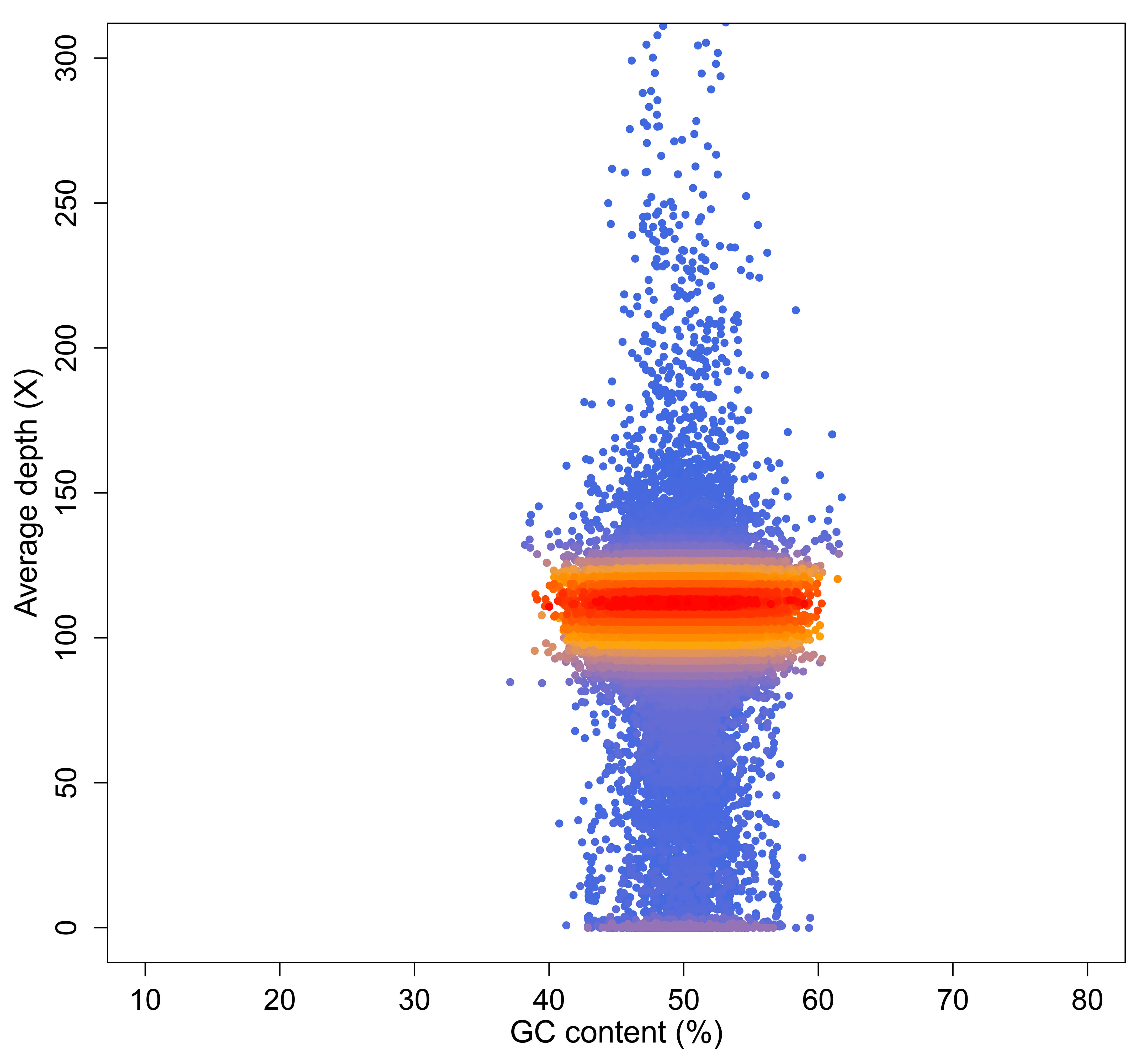


Figure S5 GC distribution of the genome assembly.


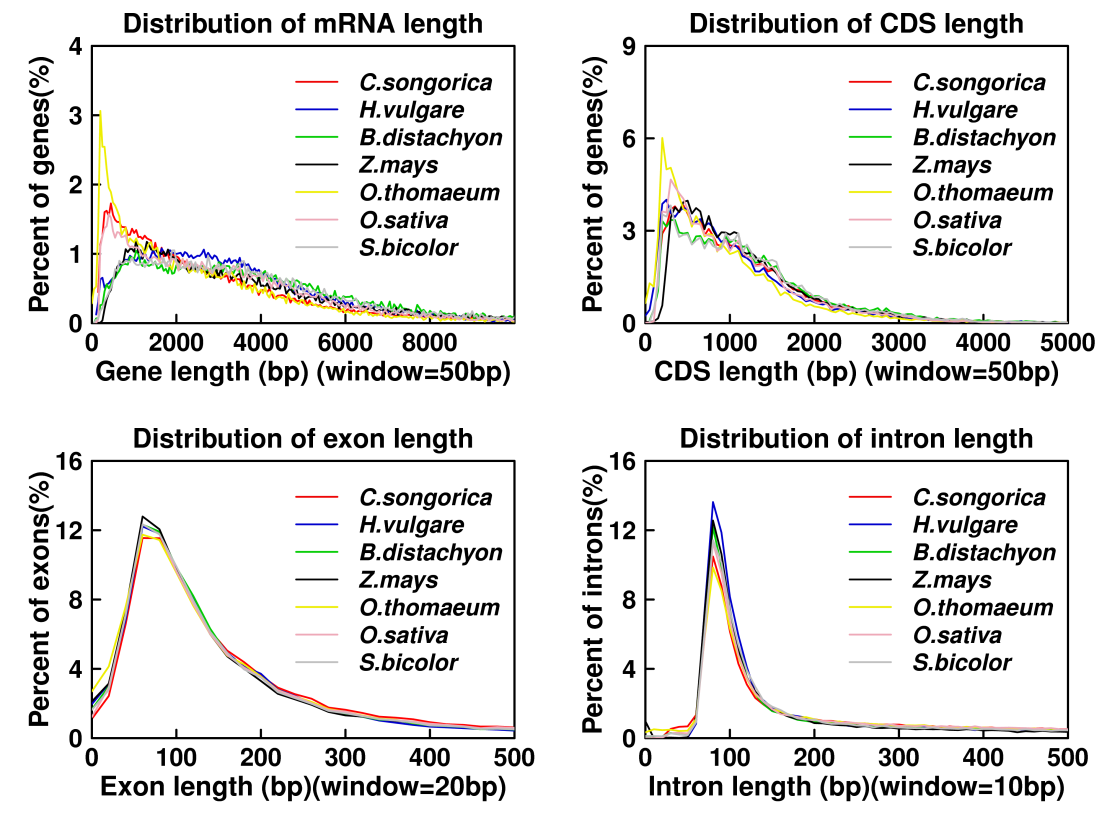


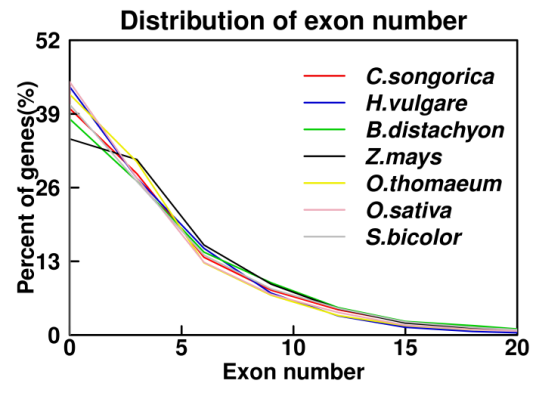


Figure S**6** Comparisons of gene structural parameters (the number and length of exons, intron length) among the seven grass species.


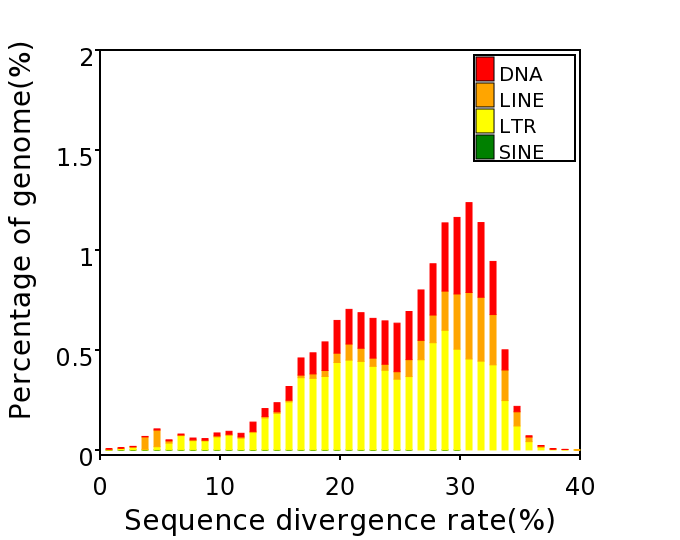

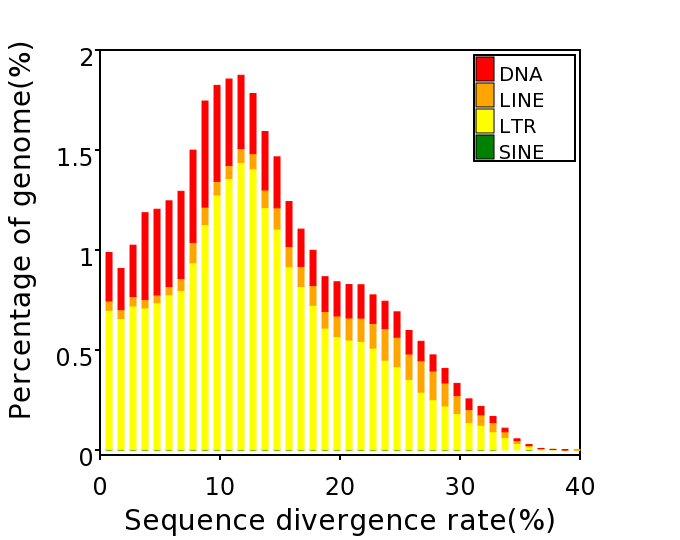


Figure S7 TEs (DNA transposons, retrotransposons LINE, LTR and SINE) sequence divergence.


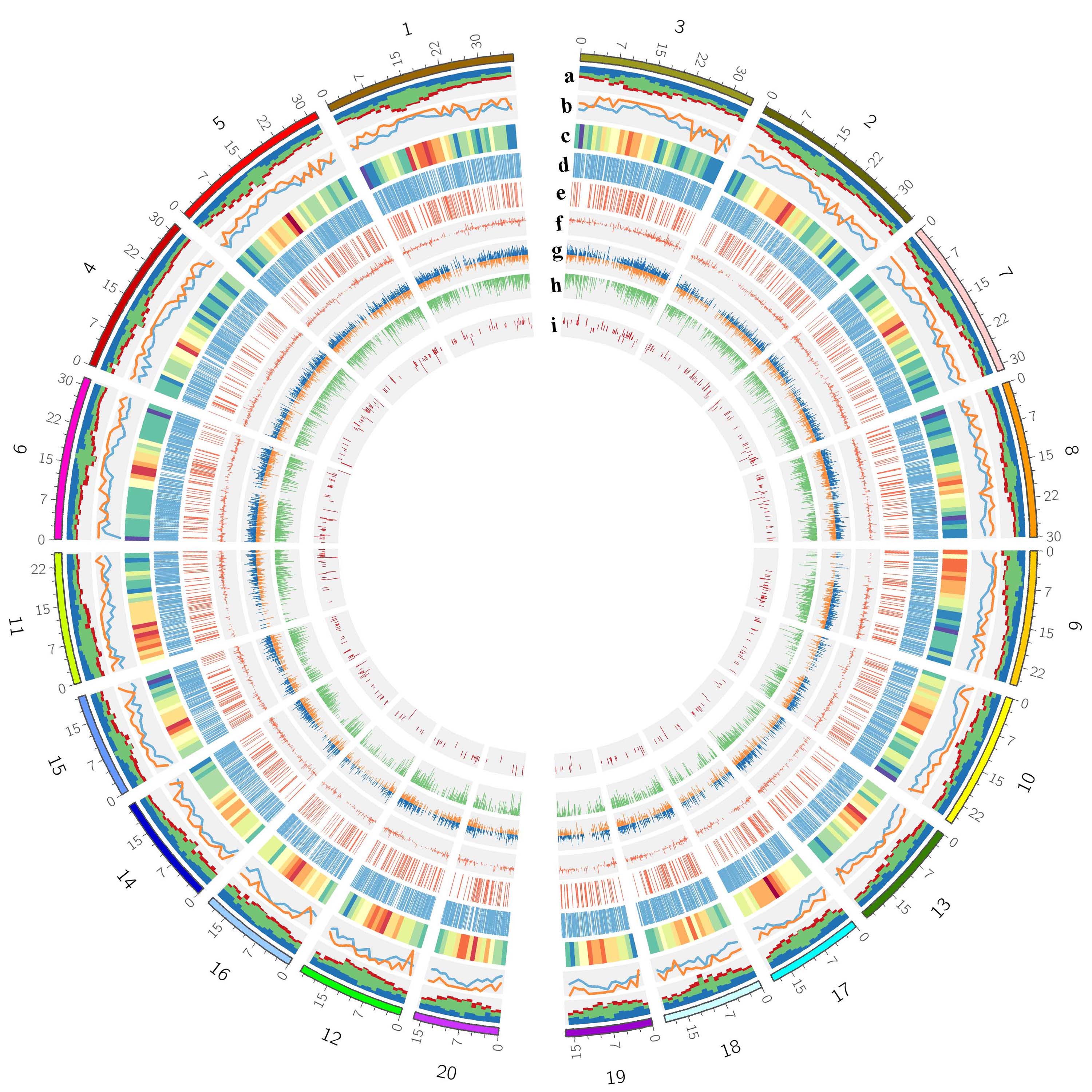


Figure S8 Genomic landscape of the 20 assembled *C. songorica* chromosomes. (a) DNA transposon, Gypsy and Copia density. (b) LINE (blue line) and SINE (orange line) density. (c) Gene density. (d) Single-copy gene locations in chromosomes. (e) Expanded gene locations in chromosomes. (f) Dominance genes density between CH and CL. (g) Dominance genes density upon low temperature (blue line) and high temperature (orange line). (h) Dominance genes density under salt treatment. (i) Differentially expressed genes density under drought stress treatments.

Ks values of CsA - CsB have a peak at 0.25, CsA - CsA and CsB - CsB don’t have a peak at this point, suggesting the WGD event was due to tetraploidization.

**
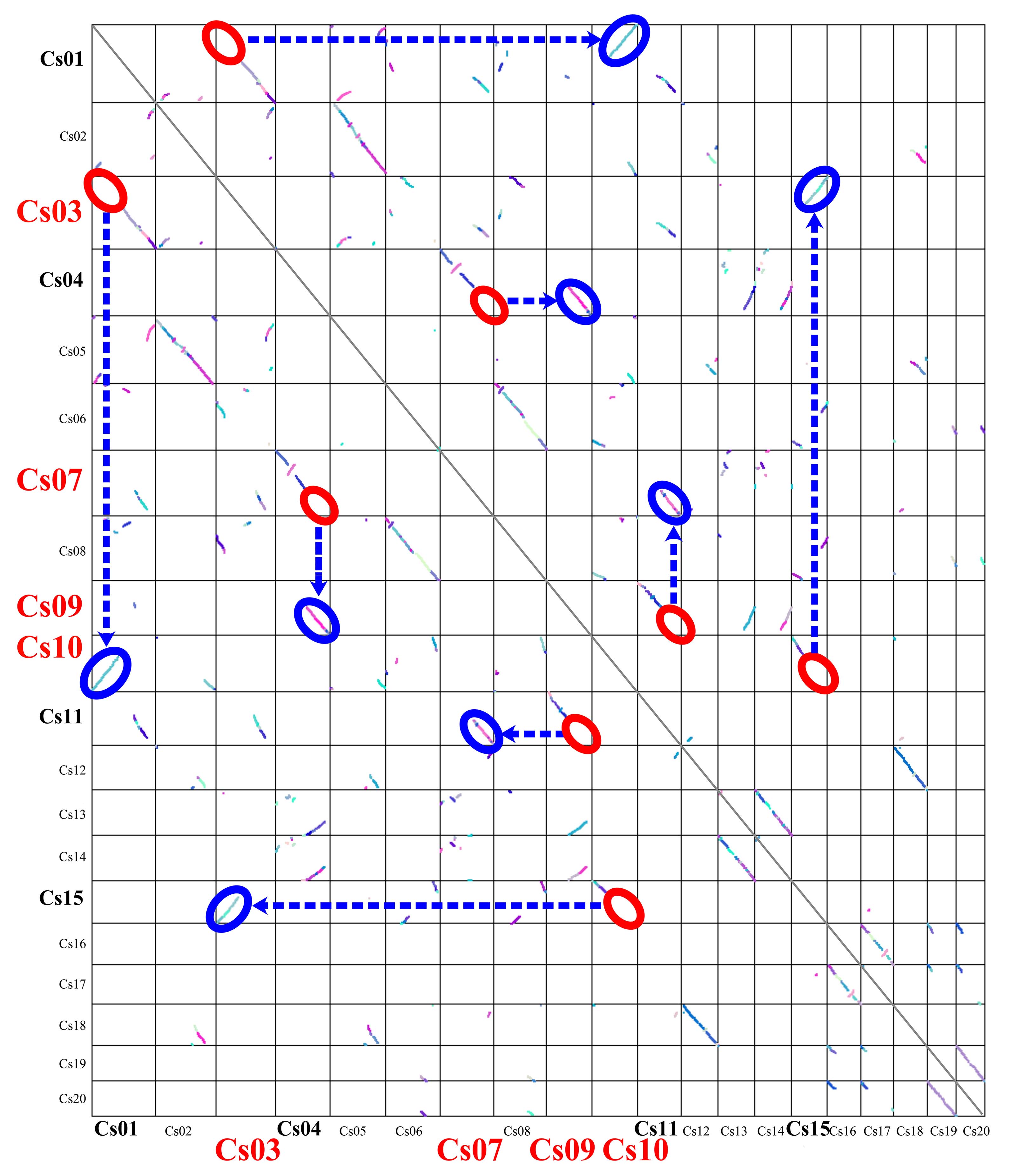
**

Figure S9 Matrix of dot plots of paralogues on the chromosomes of *C. songorica* showing reciprocal intra-genomic chromosomal rearrangements. Cs03 (B1) and Cs10 (B8), Cs07 (B2) and Cs09 (B5) chromosomal rearrangements are indicated in dotted lines. The intra-genome syntenic analysis also show that both Cs07 and Cs09 share syntenic regions with Cs04. Likewise, Cs03 and Cs10 have syntenic regions with Cs01.


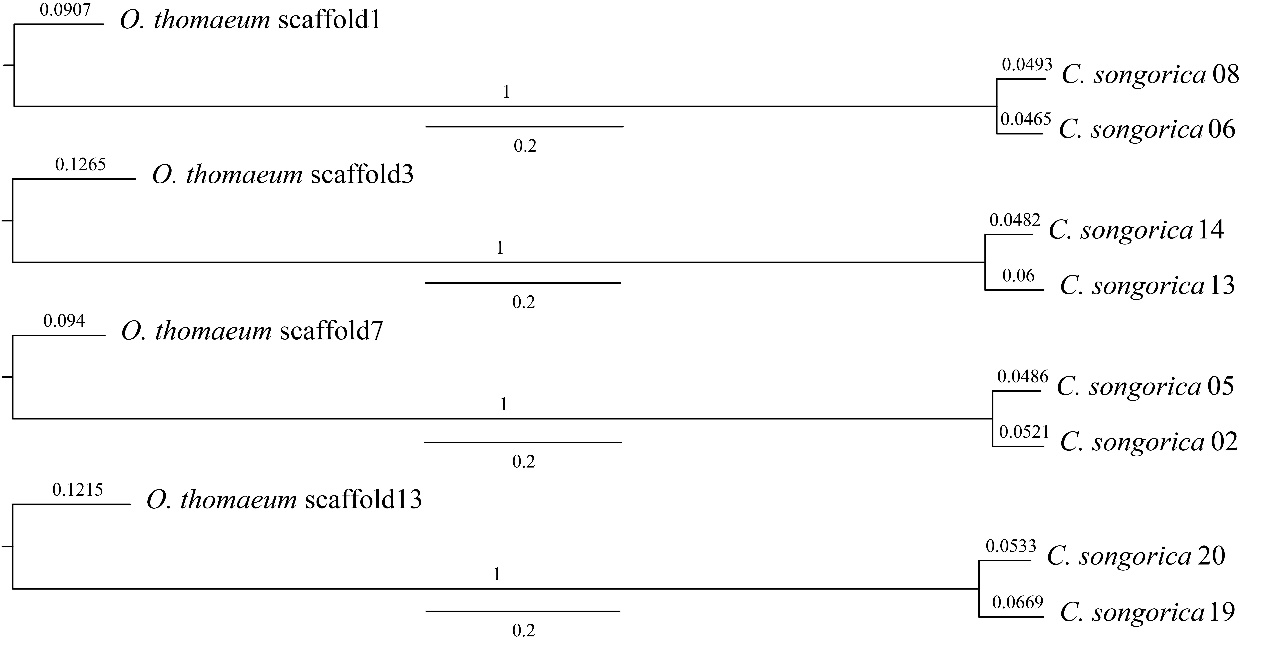


Figure S10 Phylogenic tree of *C. songorica* pseudochromosomes and *O. thomaeum* scaffolds based on single-copy orthologs. Based on single-copy orthologs, a phylogenic tree represents the genomic relationship between *C. songorica* pseudochromosomes and four *O. thomaeum* large scaffolds, suggesting the homologous chromosomes in *C. songorica*.


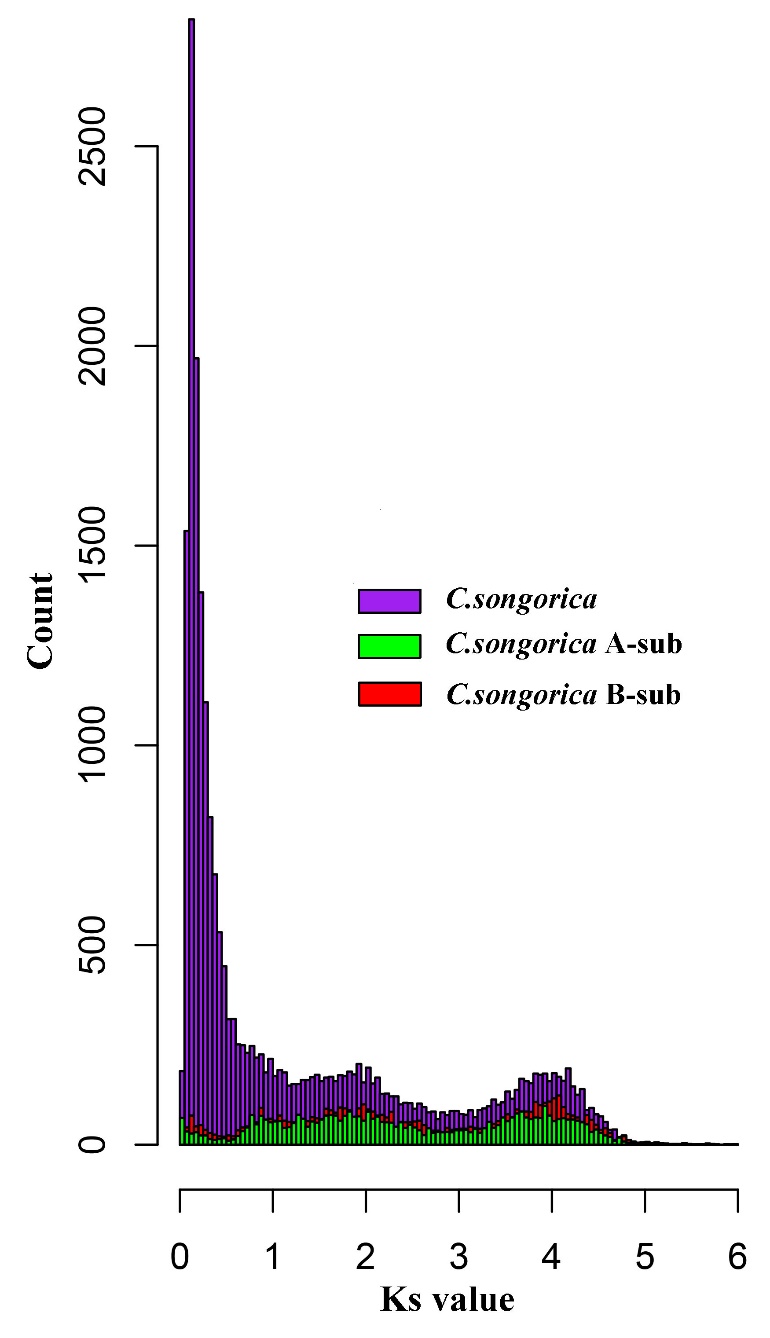


Figure S11 Distribution of Ks values.


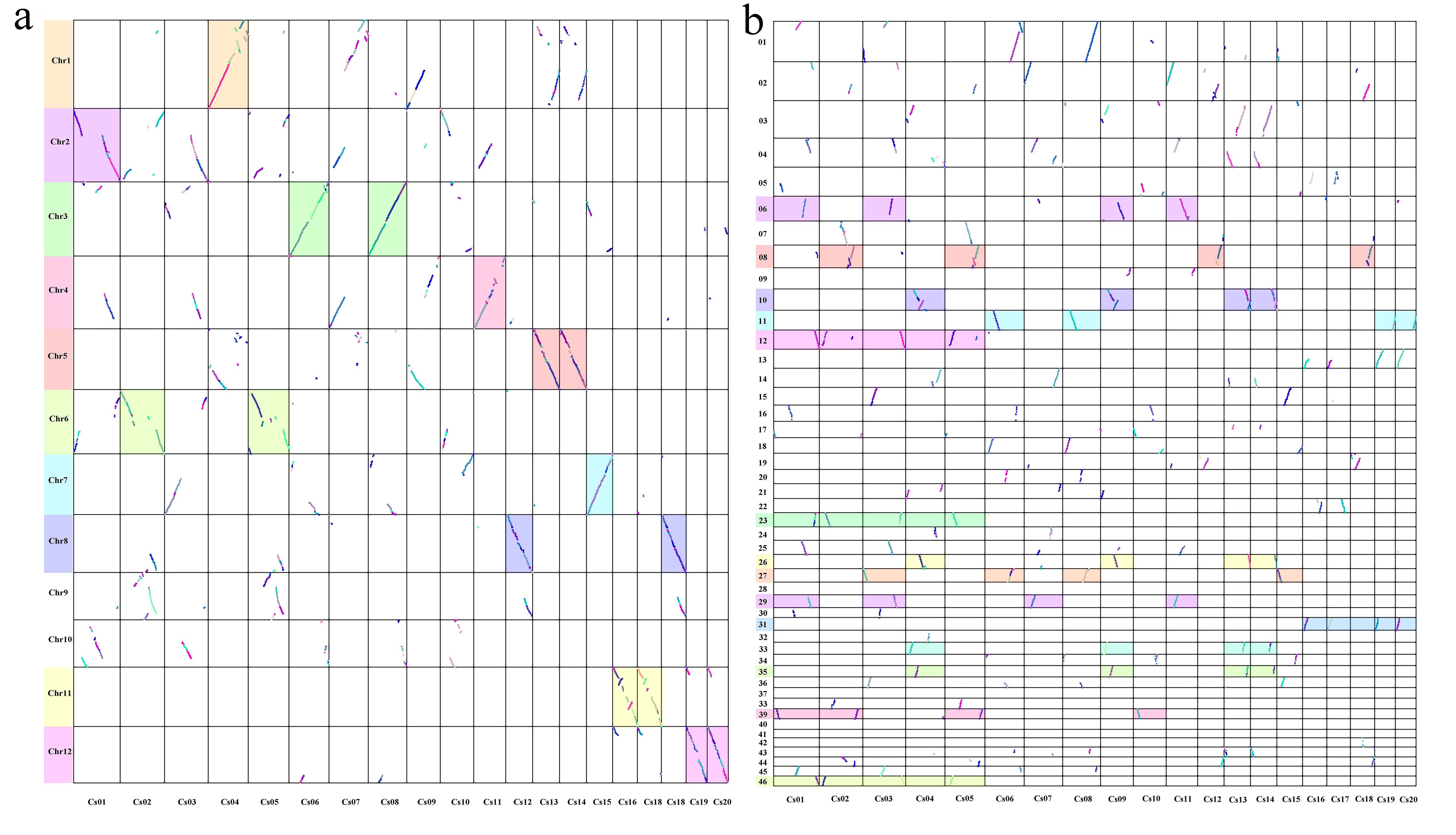


Figure S12 The syntenic of Cs - Os and Cs - Ot. (a) Syntenic of *C. songorica* and *O. sativa*. (b) Syntenic of *C. songorica* and *O. thomaeum.*

**
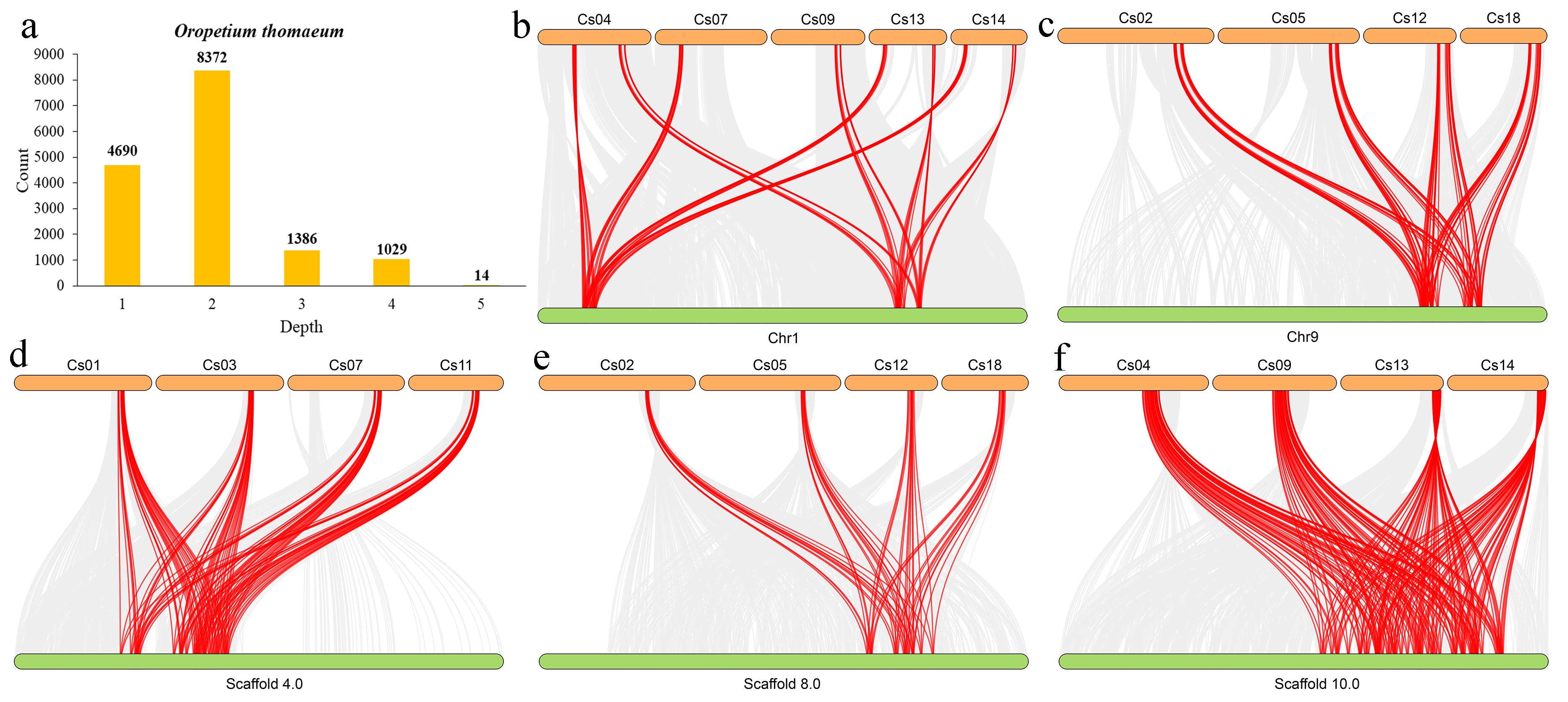
**

Figure S13 Typical micro- synteny patterns between genomic regions from *C. songorica* (Cs) and *O. thomaeum* (Ot), *C. songorica* and *O. sativa* (Os). (a) Syntenic depth of the Cs genome for each Ot. (b) Micro- synteny between Ot scaffold 4 and Cs chromosome 1, 3, 7 and 11. (c) Micro- synteny between Ot scaffold 8 and Cs chromosome 2, 5, 12 and 18. (d) Micro-collinearity between Ot scaffold 10 and Cs chromosome 4, 8, 13 and 14. (e) Micro-collinearity between Os chromosome 1 and Cs chromosome 4, 7, 9, 13 and 14. (f) Micro- synteny between Os chromosome 9 and Cs chromosome 2, 5, 12 and 18. Grey lines represent synteny gene and red lines represent that one Os gene corresponds to four Cs genes.


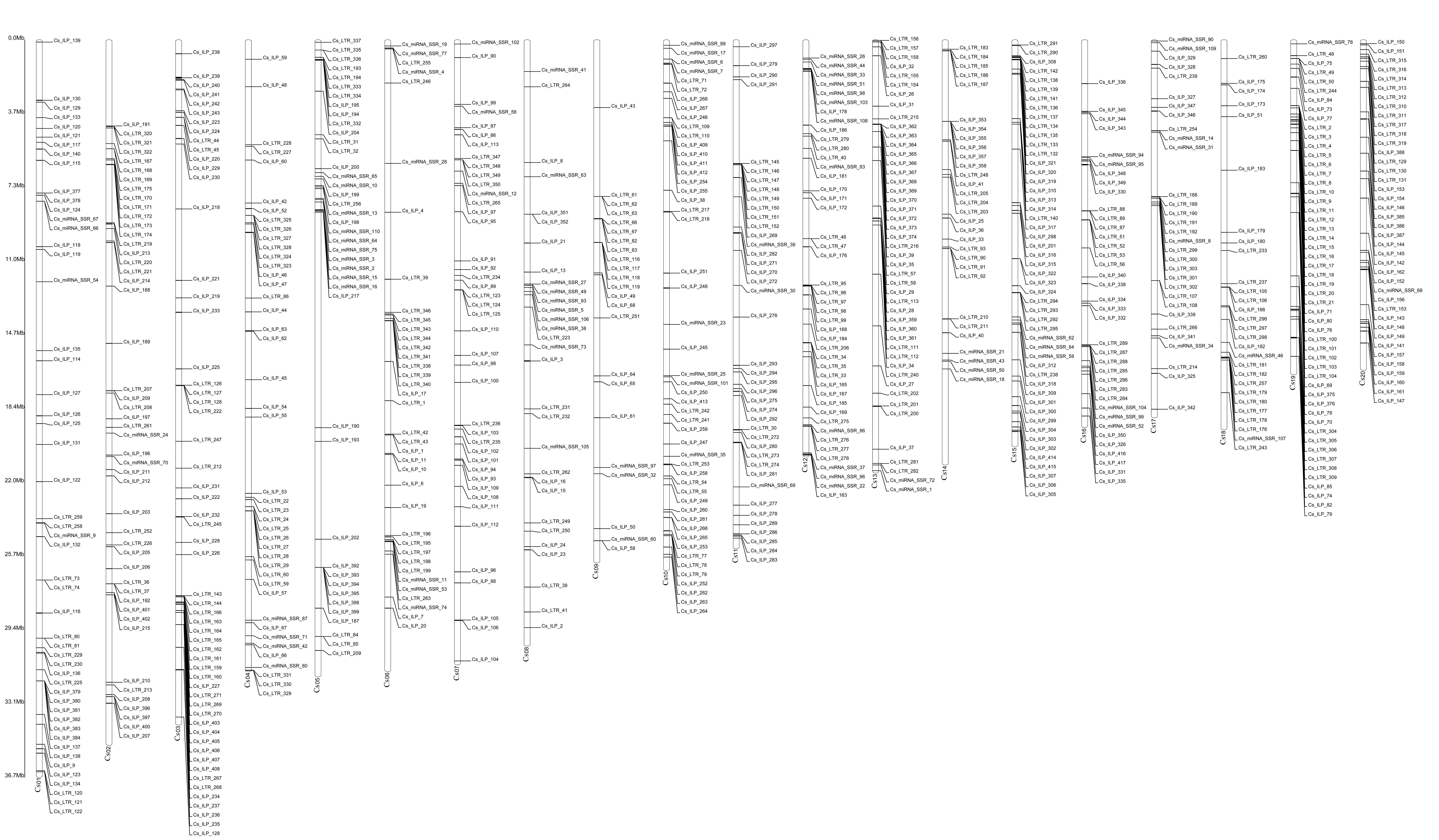


Figure S14 Locations of LTR, ILP and miRNA makers on *C. songorica* chromosomes.

Figure S15 The dendrogram of 23 Cleistogenes accessions based on UPGMA Cluster analysis using 1,119 polymorphic SSR makers.

**
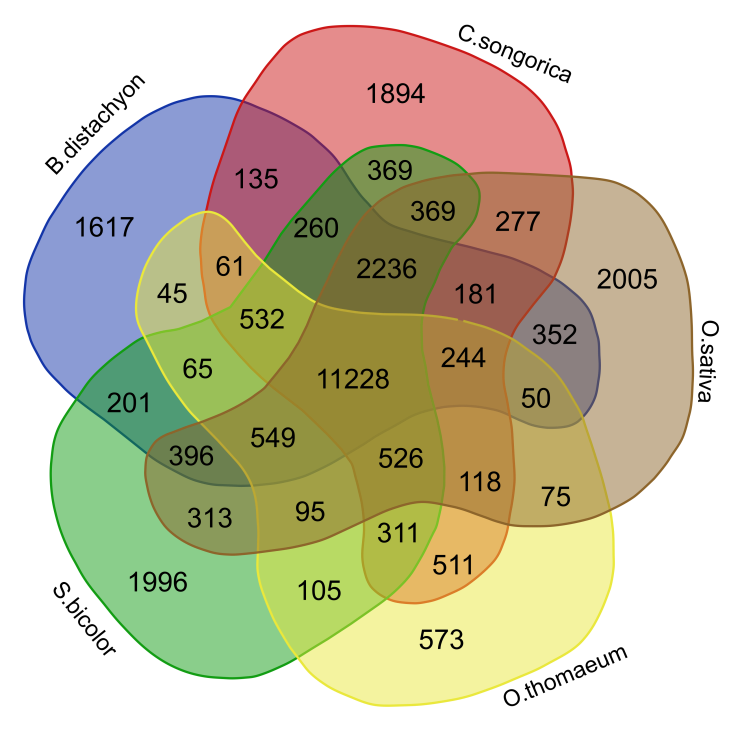
**

Figure S16 Venn plot of gene families in the five species.


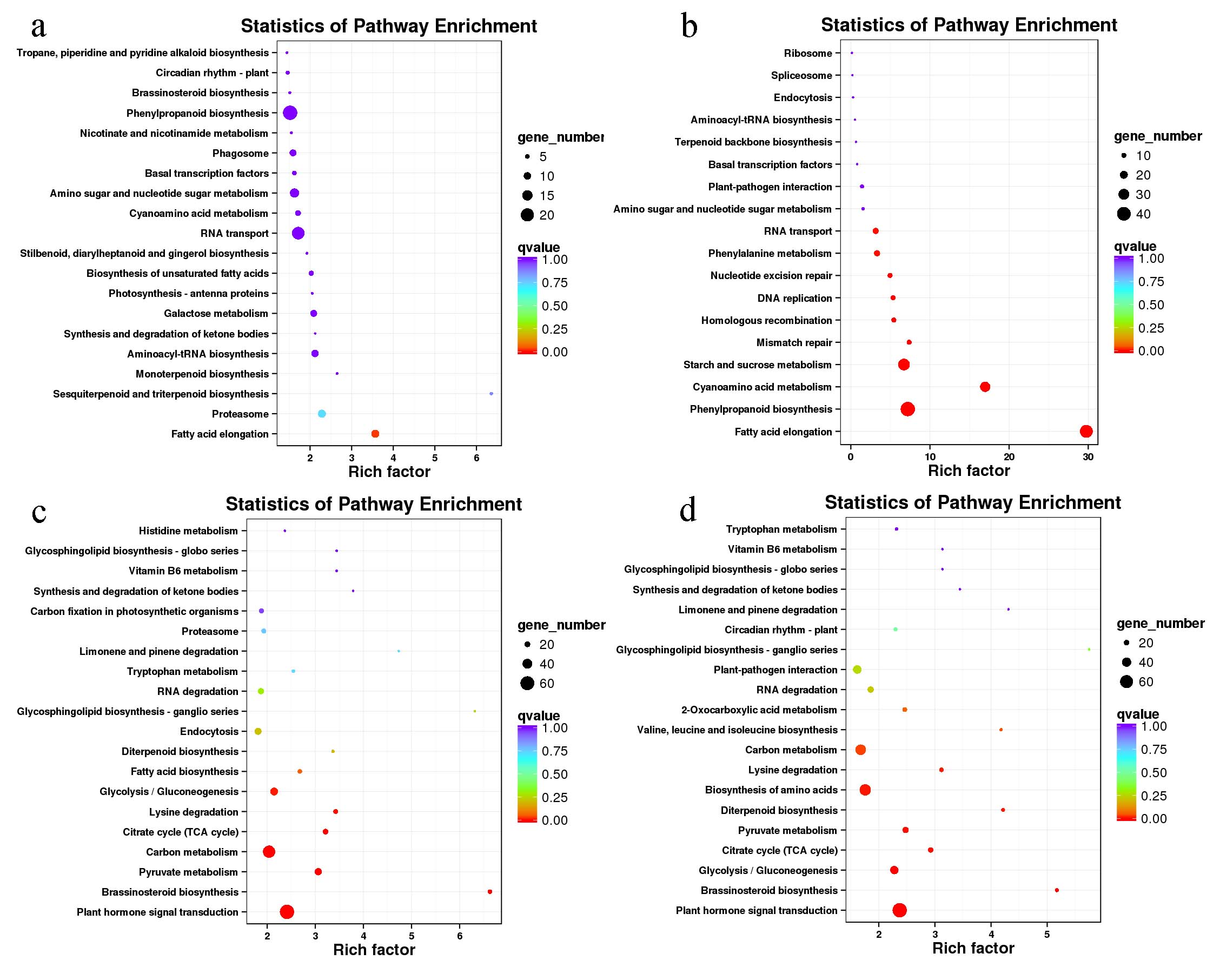


Figure S17 KEGG enrichment of *C. songorica* genes (a) KEGG enrichment of *C. songorica* unique genes. (b) KEGG enrichment of *C. songorica* expanded genes compared with other nine species. (c) KEGG enrichment analysis of *C. songorica* genes with four copies corresponding to one *O. thomaeum* homologous gene. (d) KEGG enrichment analysis of *C. songorica* genes with four copies corresponding to one *O. sativa* homologous gene.

**
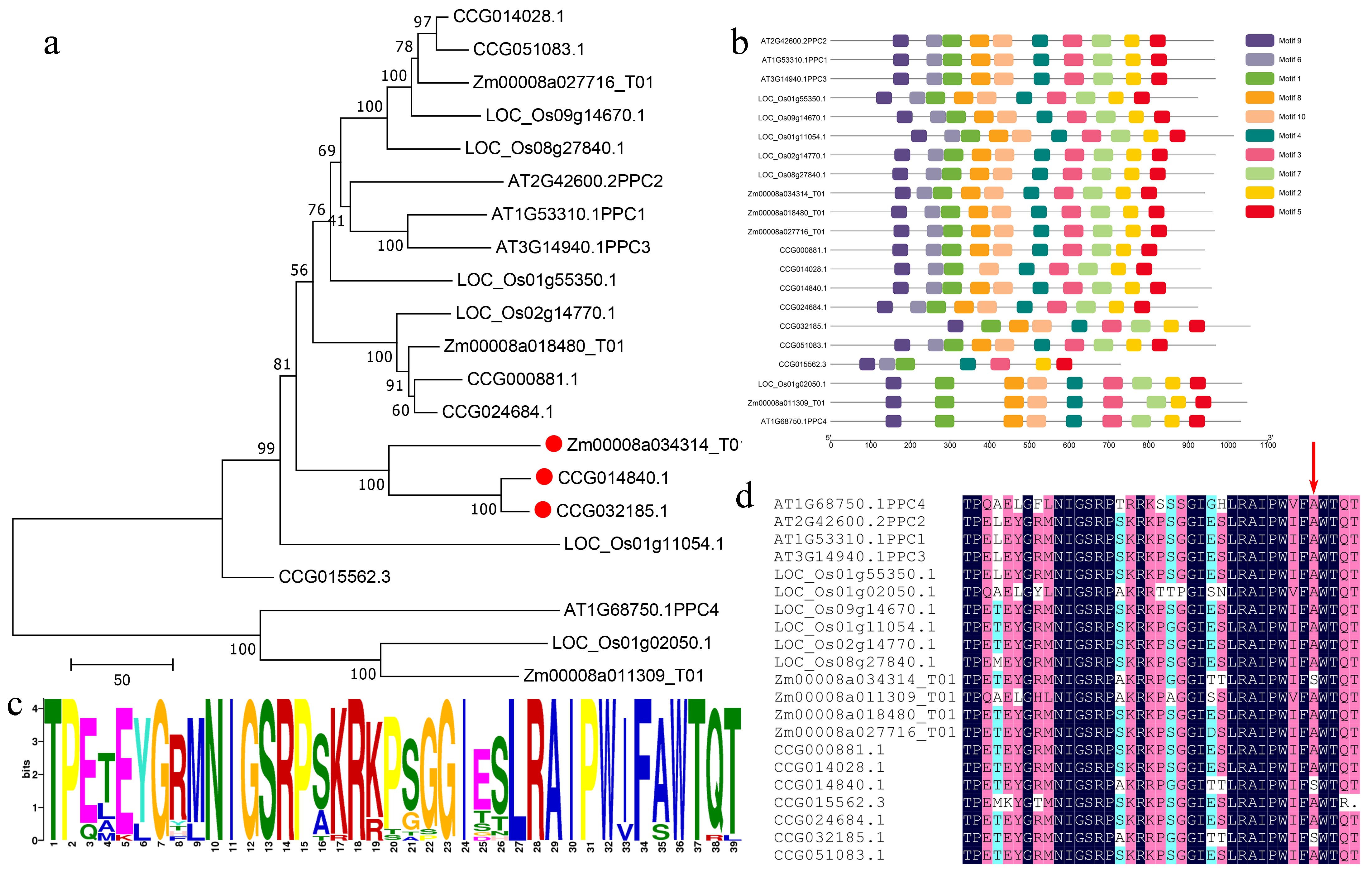
**

Figure S18 *C. songorica* PEPC gene analysis. (a) Phylogeny and distribution of PEPC peptides from *C. songorica, Z. may* and *O. sativa*. (b) Schematic representation of the ten conserved motifs in PEPC proteins. (c) Conserved motif 2 of *C. songorica*. (d) The motif 2 sequence in *C. songorica, Z. may* and *O. sativa*. Red arrow means “A” in C3 and “S” in C4 plants.


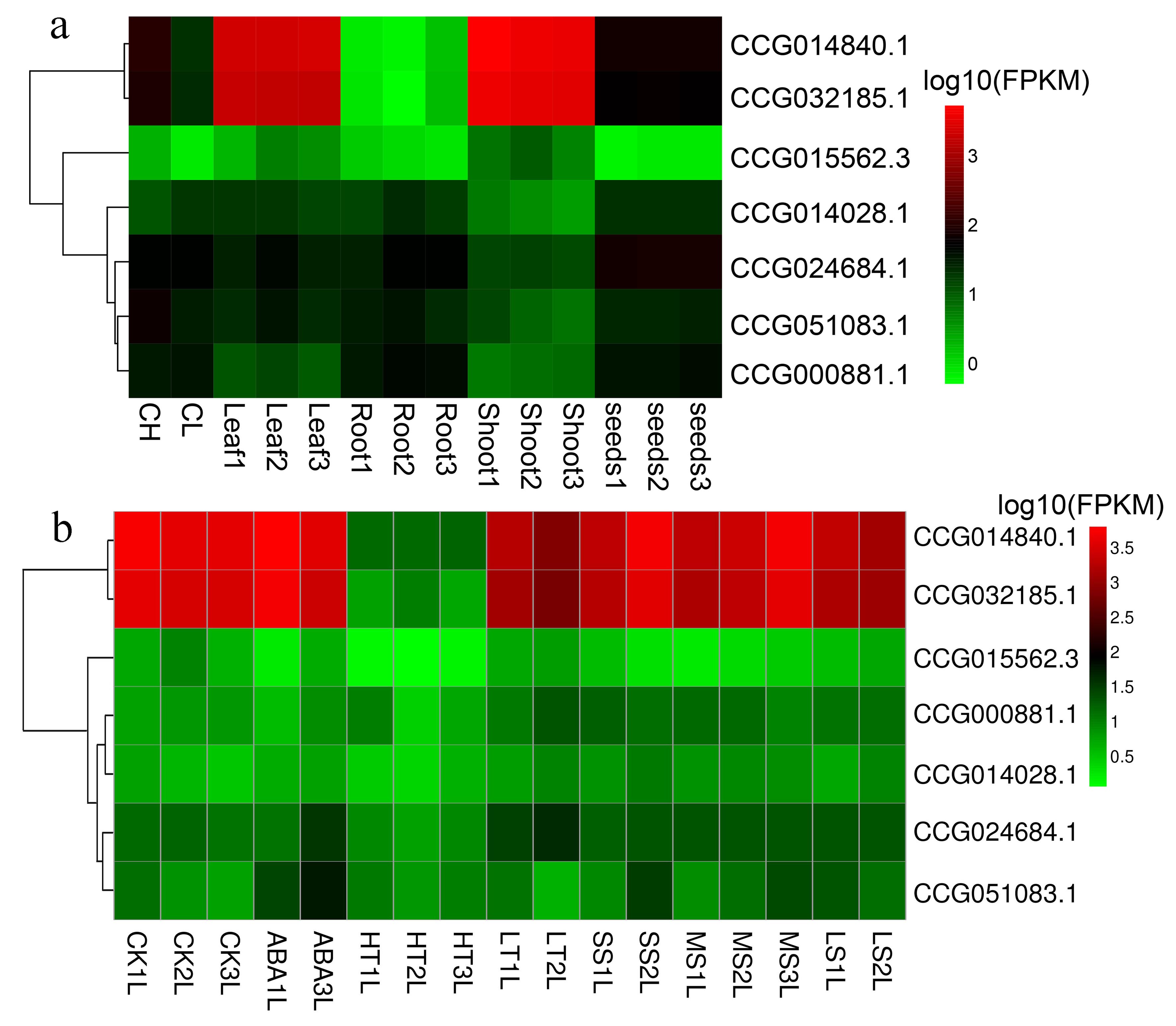


Figure S19 PEPC genes expression. (a) The expression level of PEPC genes in different tissues. (b) The expression level of PEPC genes under different treatments including control leaf (CKL), low salt stress leaf (LSL), moderate salt stress leaf (MSL), severe salt stress leaf (SSL), low temperature leaf (LTL), high temperature leaf (HTL).


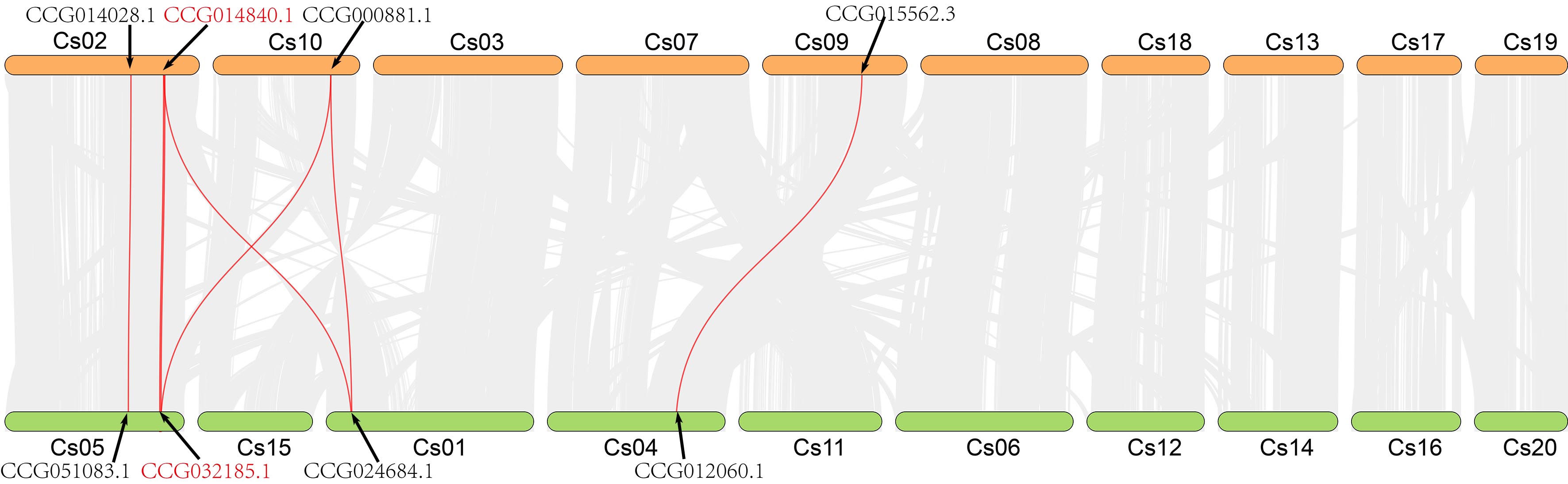


Figure S20 The PEPC genes collinearity and location on the chromosomes. Red lines mark the location of C4 PEPC genes.


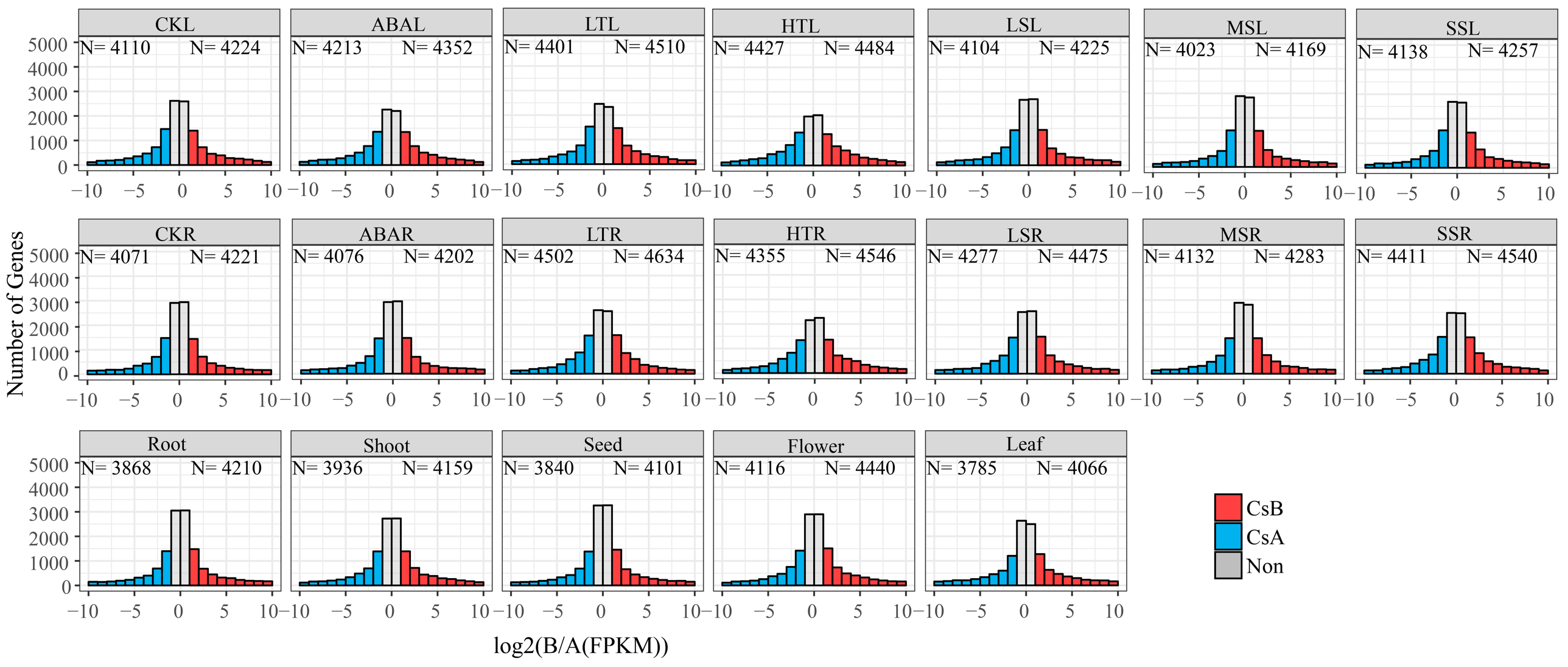


Figure S21 Homologous expression dominance in *C. songorica*. Histograms of genome-wide expression of syntenic homoeologous genes in various tissues and stress treatments. control leaf/root (CKL/R), low salt stress leaf/root (LSL/R), moderate salt stress leaf/root (MSL/R), severe salt stress leaf/root (SSL/R), low temperature leaf/root (LTL/R), high temperature leaf/root (HTL/R). N values indicate the number of dominant genes in A-sub geneome and B-sub genome, respectively.

**
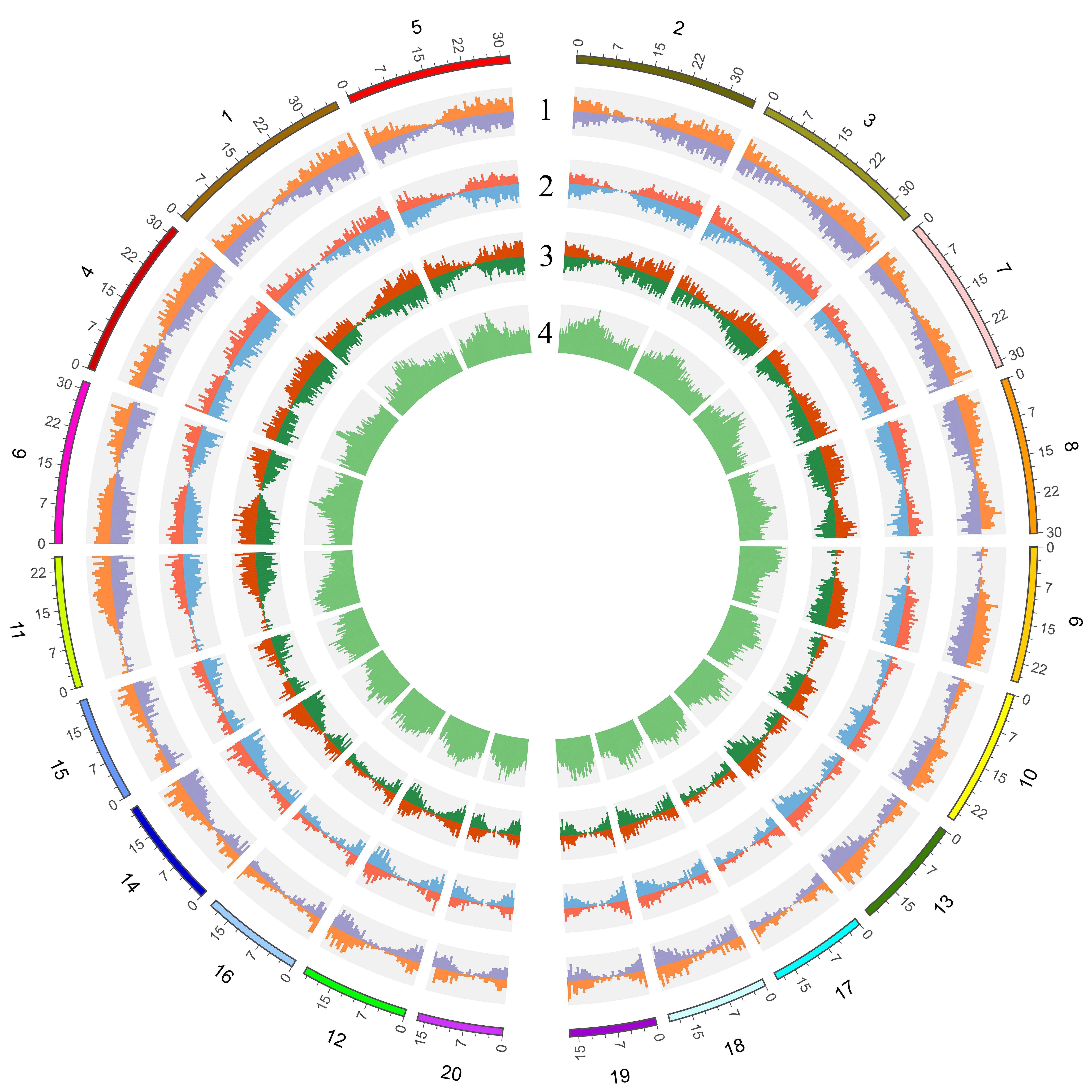
**

Figure S22 The density of dominant genes, neutral genes and TEs in A and B sub-genome. 1, Dominant and neutral genes density in all tissues; 2, Dominant genes density upon all treatments (see Figure S25); 3, neutral genes density upon all treatments (see Figure S25); 4, the density of TEs density.


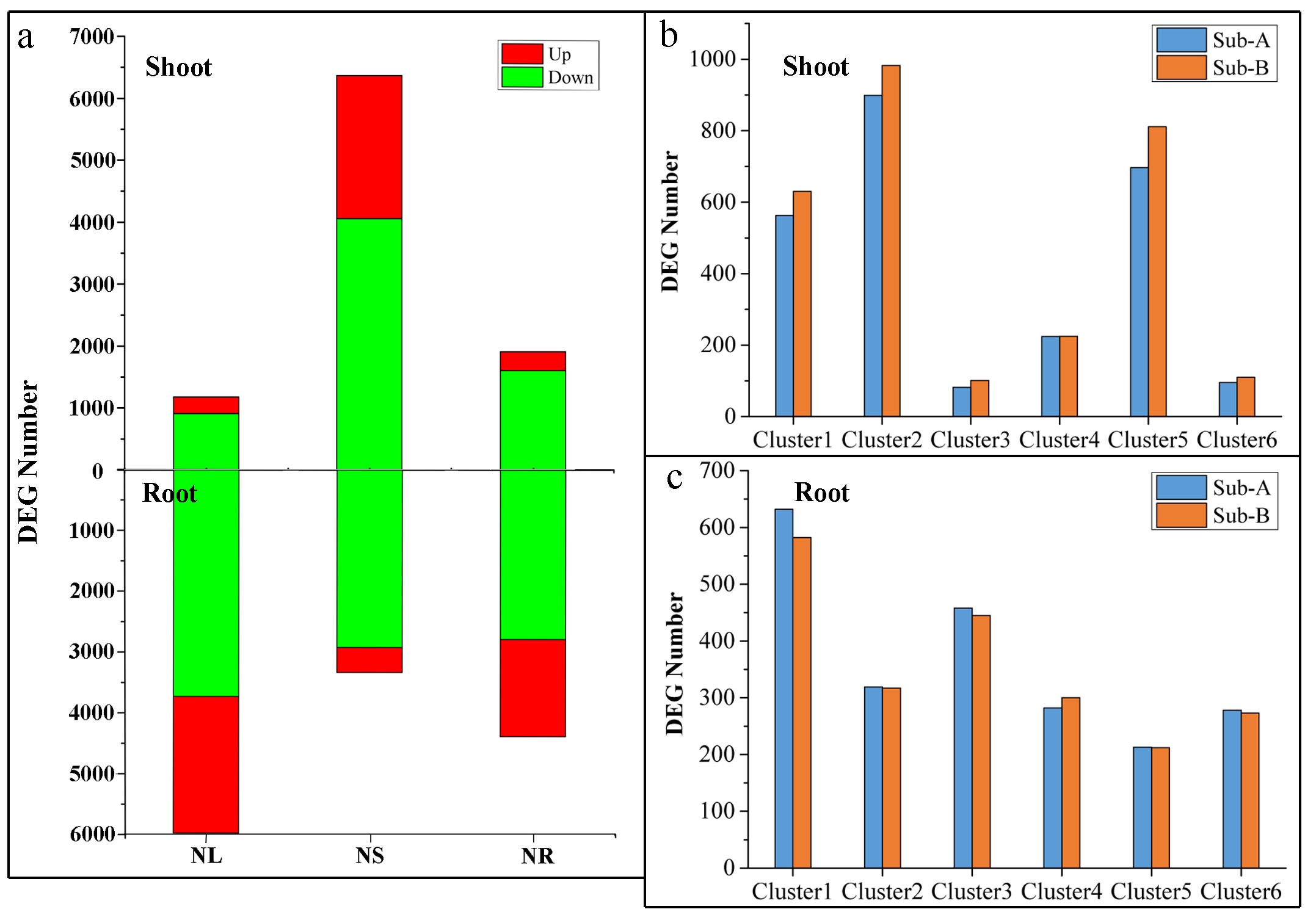


Figure S23 Differentially expressed genes (DEGs) analysis. (a) The number of DEGs under water stress and recovery in shoot and root. NL: control vs light water stress, NS: control vs severe water stress, NR: control vs recovery. (b) The distribution of shoot DEGs from *C. songorica* subgenomes. (c) The distribution of root DEGs from *C. songorica* subgenomes.

Adult plants exhibit stomata closure and reduction in carbon fixation when leaf water content drops below 55% relative water content (RWC). Higher number of DEGs were found at 48% RWC (severe water stress) compared to other treatments. Of the 6,877 DEGs in shoot, 17.11%, 92.56% and 27.74% responded to light water stress, severe water stress and recovery, respectively (Figure S29). Compared to the controls, 4,383 (65.33%), 3,332 (49.66%) and 5,972 (89.01%) genes were differentially expressed under light water stress, severe water stress and recovery in root, respectively (Figure S29). Co-expression analysis showed that the 4,475 and 5,911 DEGs formed six major clusters in root and shoot, respectively (Figure 3a, b, c, d). In root, it was evident that three major changes in gene expression occurred: between control and light water stress (NL; Cluster1 and 4), between control and severe water stress (NS; Cluster2 and 5) and between water stress and recovery (NR; Cluster3 and 6). Genes related to phenylalanine and flavone biosynthesis, plant hormone signal transduction were enriched exclusively in Cluster4, whereas genes related to thiamine and galactose metabolism, carotenoid biosynthesis are enriched exclusively in Cluster5. Genes related to butanoate metabolism and amino acid metabolism are enriched exclusively in Cluster6. Furthermore, Cluster2 are involved in lipid metabolism, glycan biosynthesis and metabolism, whereas gene function of energy metabolism and lipid metabolism are found in Cluster1. In Cluster3, genes related to nucleotide metabolism and nucleotide excision repair are decreased in water stress, then increased during recovery. In shoot, two major changes in gene expression were found: between control and severe water stress (NS; Cluster1 and 4) and between water stress and recovery (NR; Cluster2 and 5). Compared to the controls, DEGs in Cluster 2 were only decreased under severe water stress, which related to energy metabolism. Genes related to biotin and lysine biosynthesis and TCA cycle are enriched exclusively in Cluster1. DEGs were up-regulated in Cluster4 and 5, which involved in autophagy, carbon fixation in photosynthetic organisms, proline metabolism, glycan biosynthesis and metabolism. To summarize, the response mechanism under water stress were different in shoot and root. Low expression genes are related to energy metabolism and photosynthesis and high expression genes related to amino acid metabolism, plant hormone signal transduction and metabolism of terpenoids and polyketides metabolism.

**
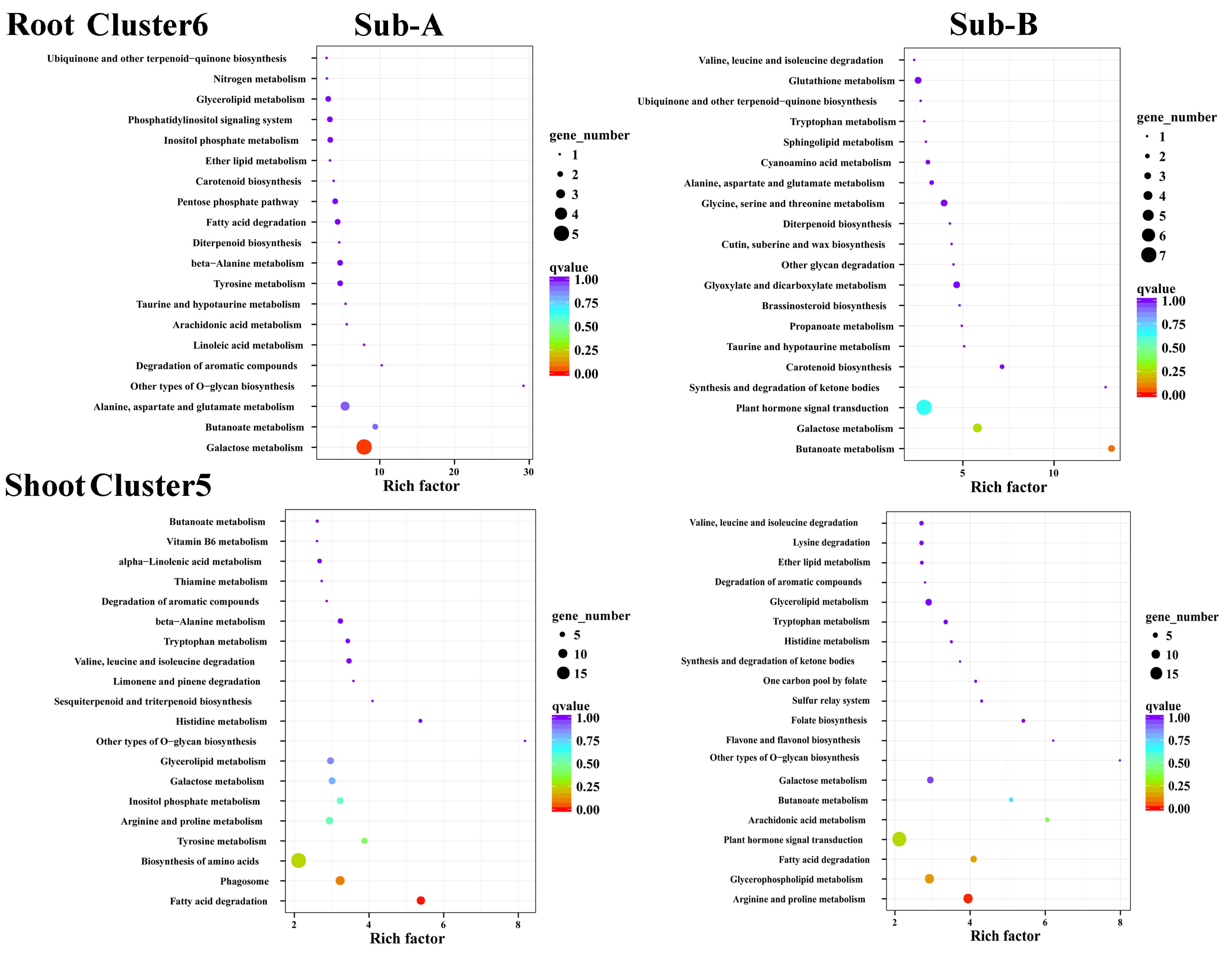
**

Figure S24 The KEGG analysis of DEGs in *C. songorica* subgenomes.


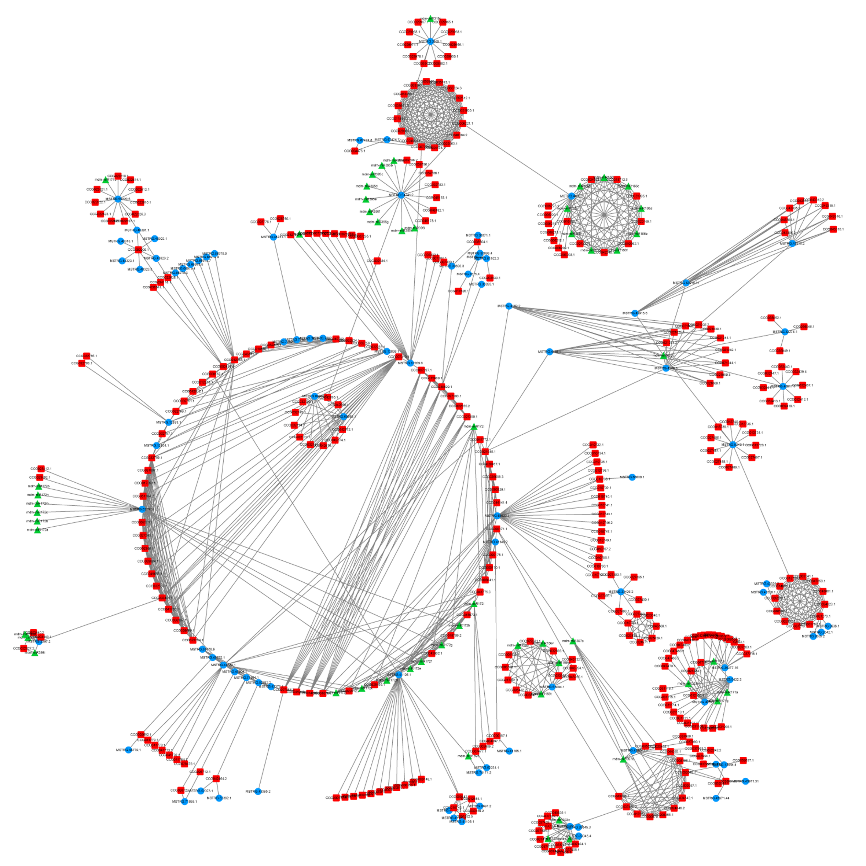


Figure S25 Representatives of predicted interaction networks among lncRNAs, Poaceae conserved genes and miRNAs.

**
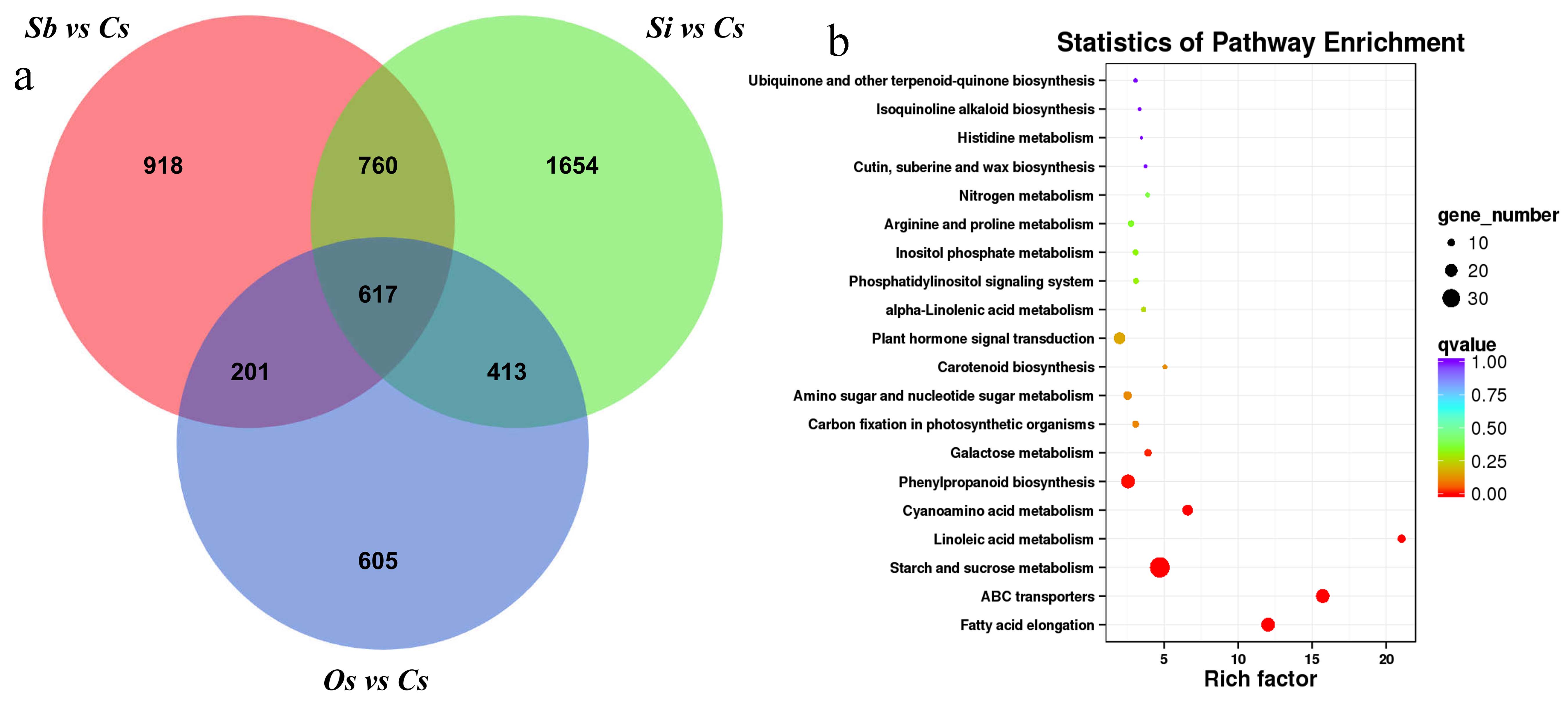
**

Figure S26 Comparisons of drought responsive genes across *C. songorica*, rice, foxtail, and sorghum. (a) The Venn diagram of putative orthologues of *C. songorica* (Cs) drought-responsive genes identified by OrthoMCL with pairwised comparisons to rice (Os), foxtail (Si), and sorghum (Sb). (b) KEGG enrichment of conserved genes in Cs, Os, Si, and Sb. The triangular, square, round nodes represent miRNAs, mRNAs and lncRNAs, respectively.

Figure S27 Distribution of significantly expanded genes in A and B subgenome.

**
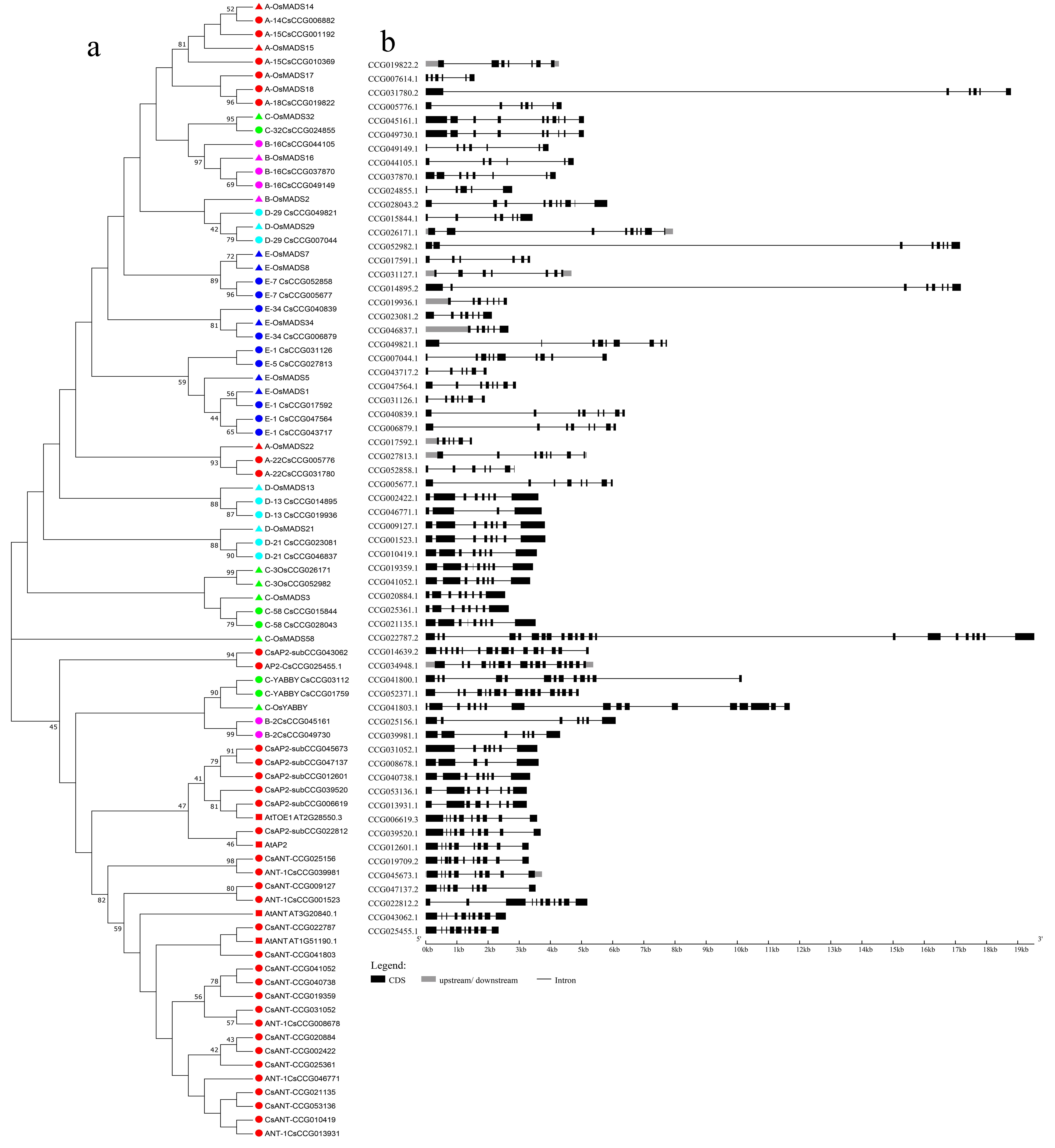
**

Figure S28 Phylogenetic analysis and genes structure of ABCDE model gene. (a) [phylogenetic](javascript:;) [tree](javascript:;) of ABCDE model gene based on amino acid sequences. (b) Structures of ABCDE model genes in *C. songorica*.


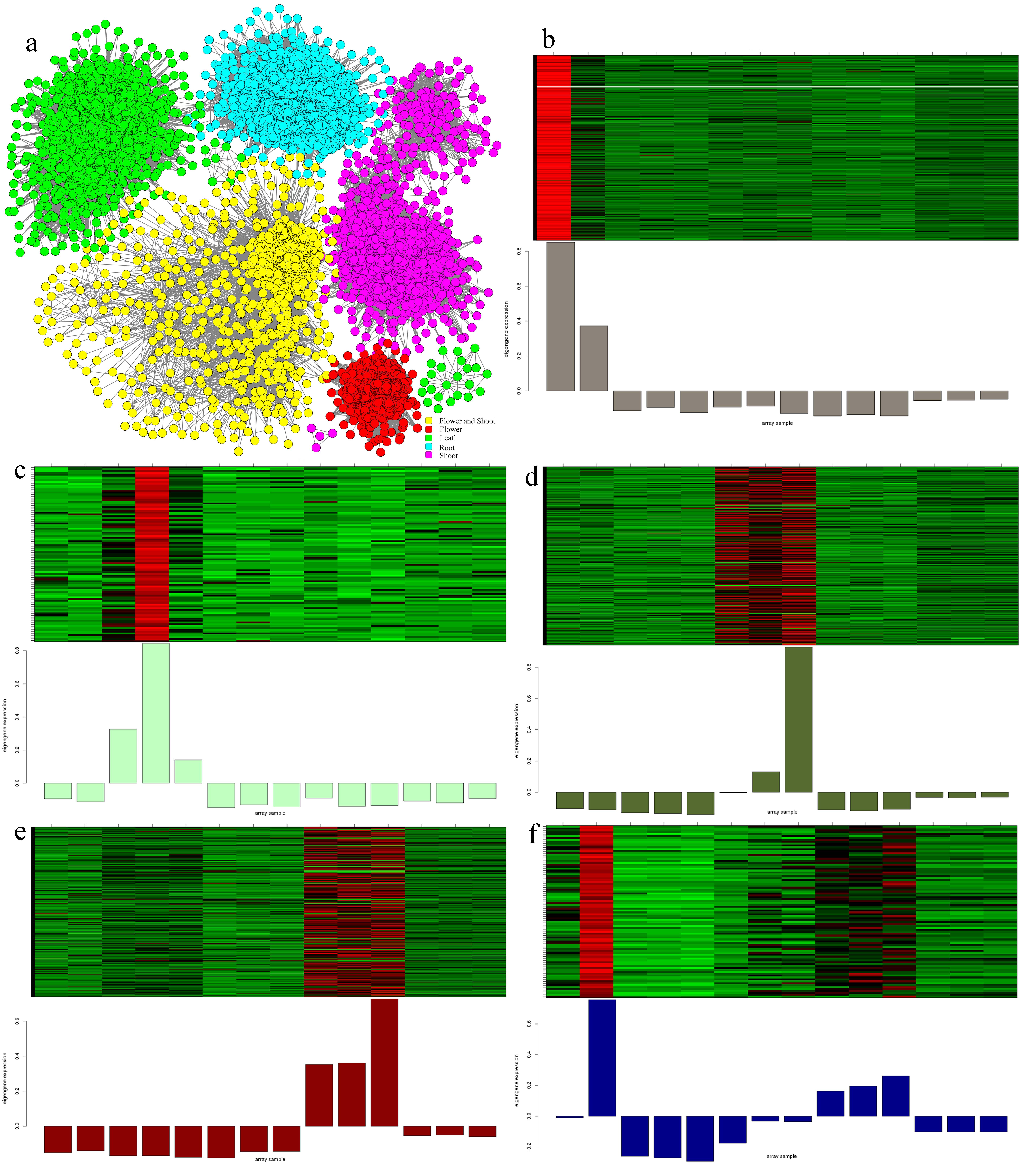


Figure S29 WGCNA analysis of tissue-specific genes. (a) Co-expression network shows strong tissue-specific effects in *C. songorica* based on WGCNA analysis. (b) Flower specific gene expression. (c) Shoot specific gene expression. (d) Leaf specific gene expression. (e) Root specific gene expression. (f) Flower and shoot specific gene expression.

The WGCNA analysis of flower, leaf, root and shoot gene expression indicated that some of genes were expressed only in one tissue. For these tissue-specific genes, the total gene number on A subgenome was similar with B subgenome. However, the tissues-specific gene pairs shared by A-sub and B-sub genome were small. For instance, among the total number of 760 flower tissues specific genes, 111 were distributed only in A subgenome, and 178 only distributed in B subgenome (Table S27). Only 91 gene pairs (containing 182 flower tissues specific genes) were found in both A and B subgenome. In allopolyploid or interspecific hybrid plant, it has been known that gene expression dominance is due to one parental ribosomal RNA genes getting silenced(Bottani et al., 2018). And genomes in a polyploid show nonequivalence in gene number distribution in its subgenomes(Schnable et al., 2011). Our results also indicate gene loss or gene silence may occur in the subgenomes of *C. songorica*.


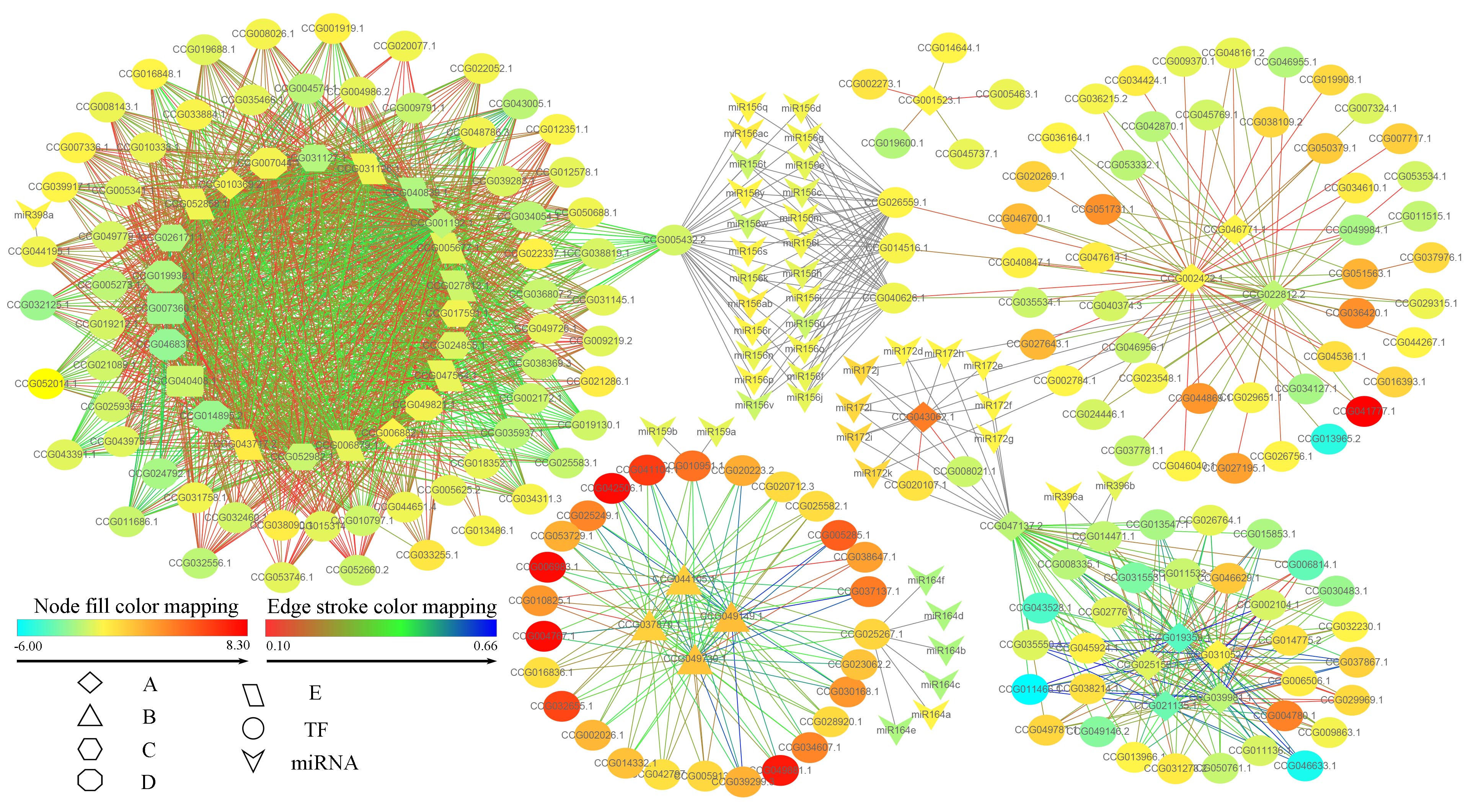


Figure S30 ABCDE model genes, TFs and miRNA co-expression network.

**
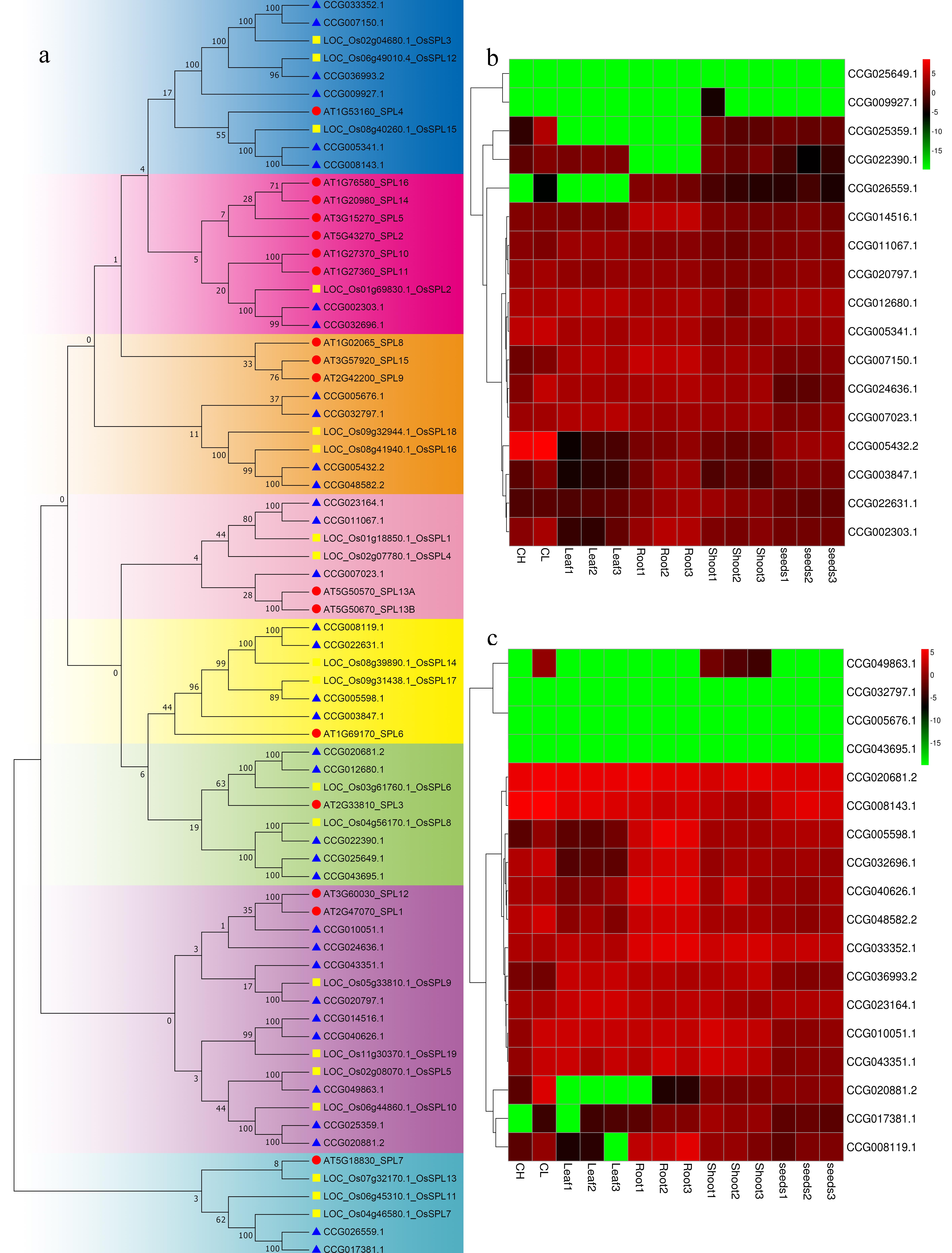
**

Figure S31 Phylogenetic and expression of SPL gene family members in *C. songorica*. (a) Phylogenetic tree of SPL proteins from Arabidopsis, rice, and *C. songorica*. The tree was generated with MAGE 7.0 software using the Neighbour-joining method. Red circles represent Arabidopsis genes, blue triangles represent *C. songorica* genes, yellow squares represent *O. sativa* genes. (b, c) The expression heatmap of A and B subgenome SPL genes.

**
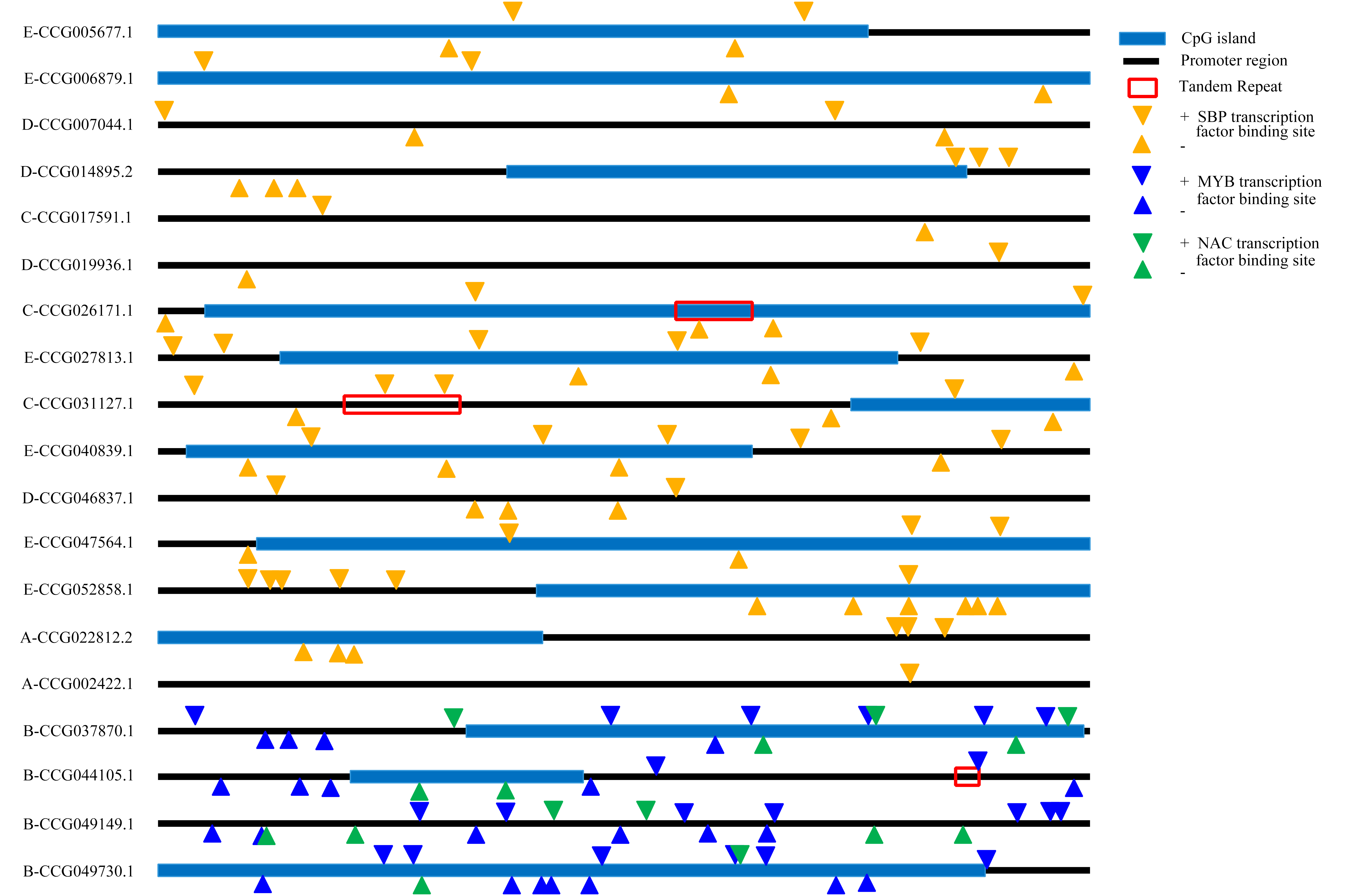
**

Figure S32 SBP, MYB and NAC transcription factor binding sites (TFBSs) in AMGs promoter region. The length of promoter region is 2000 bp. TFBSs were predicted using PlantPAN 3.0 (http://plantpan.itps.ncku.edu.tw/).

a


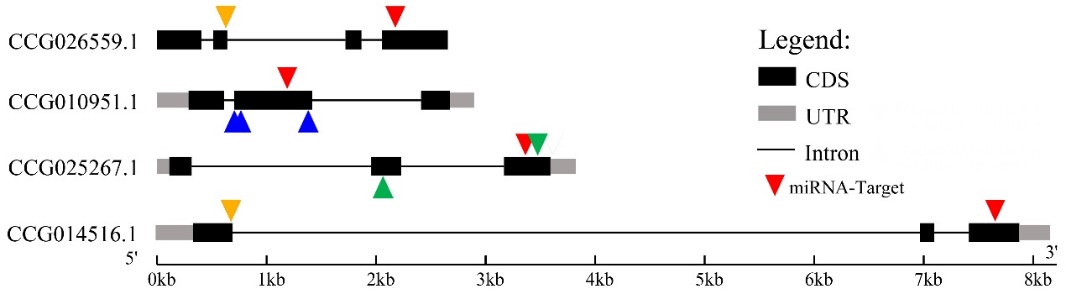

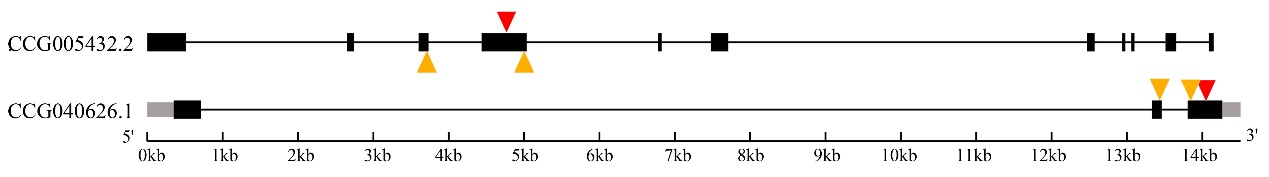


b


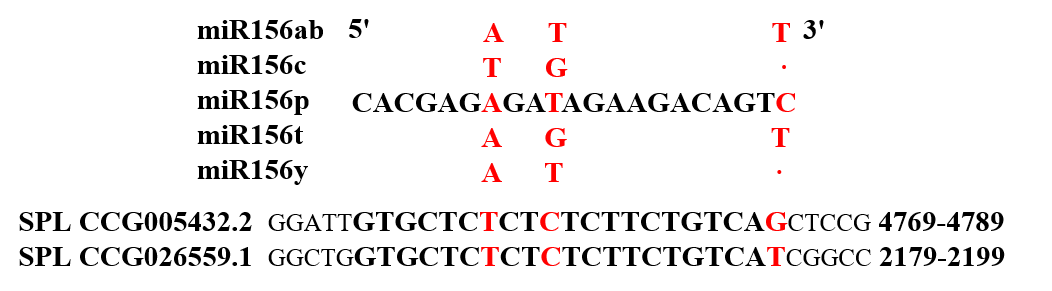

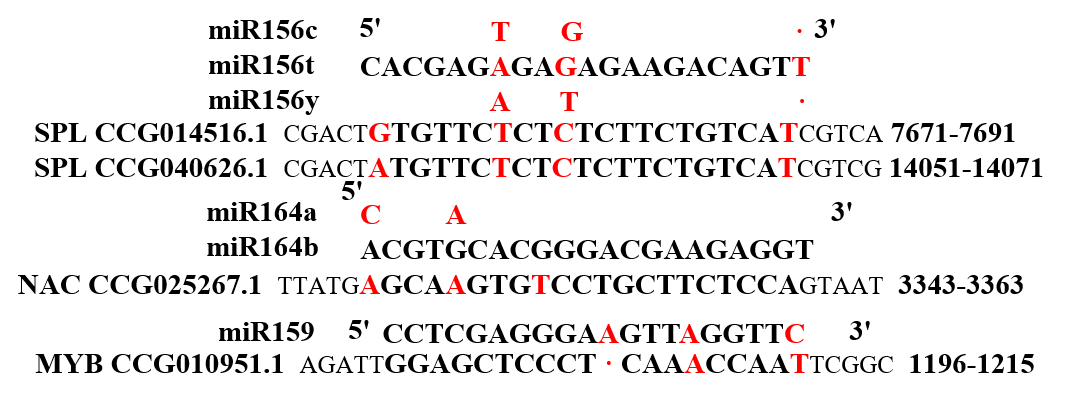


Figure S33 miRNA target sites in TFs gene in *C. songorica*. (a) Gene structure of TFs. Yellow, blue and green triangles represented SPL transcription factor binding site, MYB transcription factor binding site and NAC transcription factor binding site, respectively. (b) Sequences of miRNA-TFs target sites. Sequence variants identified in red bold.

**
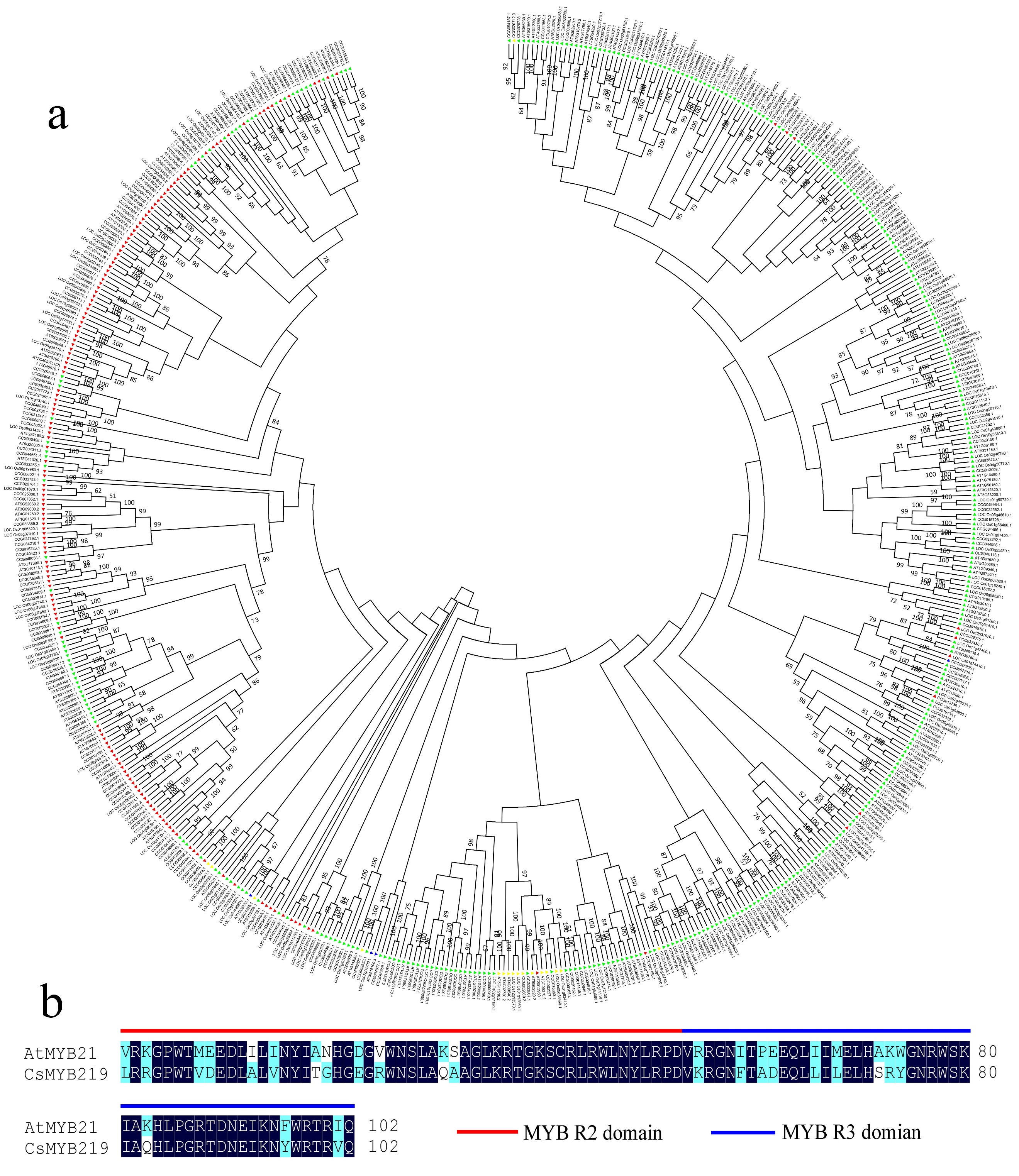
**

Figure S34 MYB gene family of Arabidopsis, rice, and *C. songorica*. (a) Phylogenetic tree of MYB proteins from Arabidopsis, rice, and *C. songorica*. Red triangle represents R1 domain type genes, green triangle represents R2R3 domain type genes, yellow triangle represents R1R2R3 domain type genes, blue triangle represents R1R2R3R4 domain type genes. (b) Amino acid alignment of AtMYB21 and CsMYB219. The conserved R2 and R3 domains are highlighted in red and blue, respectively.

**Supporting materials and** **methods**

**Methods S1** **Transcriptome analysis**

**Materials and methods**

**1. Plant materials**

Seeds were harvested in the Minqin city, Gansu Province, China. Bleach-sterilized seeds were germinated on a sand / vermiculite (1:1, v/v) mixture in a glasshouse, where the conditions were controlled at 28/24°C (day/night), with an irradiance of 150μmol quanta m^–2^ s^–1^, 16h light and 8h dark cycles, and 65% relative humidity. Four-week-old plants were transplanted in individual pots. Pots were filled with sand/vermiculite (1:1, v/v) mixture, 0.45kg per pot. Each pot was irrigated with 100mL Hoagland nutrient solution every 3 days.

**2. Drought, salt, cold, heat stress and ABA treatment**

Before the drought treatment, the water content of the soil was controlled around 90 - 95%. When the plants grow for 6 weeks the 24 pots were randomly divided into four groups including control (NN), light drought stress (NL), severe drought stress (NS) and 48h recovery group (NR). The initial soil water content of the NN control group remained unchanged, while the soil water content of the LS and HS was decreased to 6-10%, 1-3%, respectively. The NR group plants were re-watered after the soil water content decreased to 1-3%. Shoot and root samples were then collected 48h after rewatering. Each treatment had three independent biological replicates.

Nine-week-old plants were divided into seven groups. These groups were treated with 50mM/L NaCl,100 mM /L NaCl, 200 mM /L NaCl, representing light, middle and severe level of salinity; under heat and cold (40°C and 4°C); and with 100μM ABA treatment. Untreated plants were used as the control group. Shoots and roots of each plant were collected 24h after treatment, and immediately immersed in liquid nitrogen, and stored at -80°C.

**3. Flower, leaf, root, seeds and shoot materials**

For tissue-specific transcriptomes, root, leaf and shoot samples were obtained from the 6-week-old plants. Seed samples (from CH and CL flowers) were mixed, including unmatured, imbibed and germinated in 2 days. Three biological samples were collected, immediately frozen in liquid nitrogen, and stored at -80°C. Flower samples were collected in Yuzhong campus of Lanzhou University (N35°57', E104°09'), Gansu Province, China, included: CL flowers at green stage(Wu et al., 2018) and CH flowers. smallRNAs samples were collected from field included CH spike, spikelet of CL_U nodes (upper nodes, from 7 to 9), CL_M nodes (middle nodes, from 4 to 6) and CL_B nodes (bottom nodes, from 1 and 3 nodes).

**4. RNA sequencing**

Total RNA of each sample was isolated using the TRIzol reagent (Invitrogen) and following the manufacturer's instructions. For salt, cold, heat, ABA stress treatment and tissue-specific samples, mRNAs were separated from the total RNA by Oligo (dT) and were cleaved into short fragments at random. For drought stress treatment, ribosomal RNA was removed by Epicentre Ribo-zero™ rRNA Removal Kit (Epicentre, USA), and rRNA free residues were cleaned up by ethanol precipitation. The sequencing libraries were prepared by NEBNext® Ultra™ Directional RNA Library Prep Kit for Illumina® (NEB, USA) following manufacturer’s recommendations. The cDNA libraries were constructed by PCR enrichment and sequenced in paired-end on HiSeq2500 with read length of 125 bp. For flower samples, the cDNA library construction and sequencing were based on the Illumina HiSeq 2000 platform (San Diego, CA, USA). SmallRNAs from flowers were sequenced on Illumina HiSeq 2000 platform(Wu et al., 2018).

**Methods S2 Identification of flower development related genes**

**Materials and methods**

**1. Co-expression network of flower related genes, TFs and miRNAs**

miRNAs were identified through Blast against the miRBase 19.0 (<http://www.mirbase.org/>). miR156, miR59, miR171, miR172, and miR396 were known to regulate floral organ identity genes expression, affecting lemmas, paleas, lodicules etc^(Smoczynska and Szweykowska-Kulinska, 2016)^. miR171, miR399, miR824, miR156, miR319, miR393, miR159, miR169 target genes were predicted using PsRobot (<http://omicslab.genetics.ac.cn/psRobot/)>.

**2. Real time quantitative RT-PCR**

*C. songorica* fresh tissue from lemma, palea, anther, pistil and CH and CL flower primordium were separated under dissecting microscope (SZ2-ILST, Olympus Corporation, Tokyo Japan), and stored in RNA-Be-Locker A reagent, which helps permeate tissues, stabilize and protect RNA expression and prevent RNA degrading. mRNA was extracted for qRT-PCR using RNAiso regent (TaKaRa, Dalian, China). Reverse transcription was performed according to the manufacturer’s instructions of PrimeScript® RT reagent Kit (TaKaRa). The expression of some AMGs were quantified on Applied biosystems 7500 Real-Time PCR System using 2xSG Fast qPCR Master Mix (Low Rox) kit using mRNA specific primers (designed using PerlPrimer software) (Table S**21**). Normalization was performed relatively to *CsGAPDH* and the data were collected with three technical replicates per sample.

**Results**

**1. Flowering genes**

We compared the genome-wide homoeologous flowering related genes in *C. songorica* with that from At and Os. The well-studied At flowering gene network comprises around 200 genes in different flowering pathways. Dependent on At flowering gene network(Haider, 2014; Teotia and Tang, 2015), we grouped *C. songorica* flowering genes into five major pathways: vernalization, photoperiod, autonomous, gibberellin acid (GA) and age pathway. Photoperiod pathway includes 10 members: *CONSTANS* (*CO*), *FLOWERING LOCUS T* (*FT*) , *CIRCADIAN CLOCK ASSOCIATED 1* (*CCA1*), *TIMING OF CAB EXPRESSION1* (*TOC1*) and *EARLY FLOWERING 3 (ELF3)*(S et al., 1999), *CYCLING DOF FACTOR* (*CDF*) and FLAVIN*-BINDING KELCH REPEAT, F-BOX 1* (*FKF1*)(Sawa et al., 2007; Takato et al., 2003), *CONSTITUTIVELY PHOTOMORPHOGENIC (COP1)*(Liu et al., 2008), *GIGANTIA* (*GI)*(S et al., 1999), *SUPPRESSOR OF PHYA1 (SPA1)*(Sascha et al., 2006). *PHYTOCHROME A (PHYA)* and *CRYPTOCHROME 2 (CRY2)*. Gibberellic acid (GA) pathway includes 3 members: *SUPRESSOR OF OVEREXPRESSION OF CONSTANS 1 (SOC1), LEAFY (LFY),* and *FT*. Vernalization pathway includes 2 members: *FRIGIDA* (*FRI*) and MADS box transcription factor *FLOWERING LOCUS C* (*FLC*)(Johanson et al., 2000; Michaels and Amasino, 1999), *SHORT VEGETATIVE PHASE (SVP)*(Jeong Hwan et al., 2007). Autonomous includes 7 members: *FCA, FPA,* and *FLOWERING LOCUS KH DOMAIN (FLK), FY*, *FVE* and *FLOWERING LOCUS D* (*FLD*)(Bäurle and Dean, 2006), *FLOWERING LOCUS M* (*FLM*)(Sureshkumar et al., 2006).We identified 83 gene families containing 302 genes related to flowering, and then built a network of these genes (Figure 4a).

We constructed phylogenetic trees of *C. songorica* flowering genes and those from Arabidopsis and rice. Based on the phylogenetic relationship *C. songorica* flowering genes were identified when they were clustered in the same clade with the annotated genes. In this way, we found *CsPRR* (6 genes), *CsLHY/CsCCA1* (2), *CsCHE* (5), *CsLUX* (2), *CsELF4* (1), *CsTOC1* (2), *CsCDF1* (4), *CsCDF2* (8), *CsFKF1* (2 )genes, *CsZTL* (4), *CsCRY1/2* (3), *CsCOP1* (2), *CsELF3* (4), *CsPHYA* (4), *CsPHYB* (2), *CsPHYE* (2), *CsPFT1* (1), *CsCO* (9), *CsSPA* (6), *CsHAP5* (10), *CsHAP3* (15), *CsCIB1* (4), *CsTEM* (4), *CsFT/CsTSF* (11), *CsSPL3* (2), *CsFD* (1), *CsCOL9* (7), *CsRFI2* (2), *CsFVE* (2), *CsFLK* (5), *CsPEP* (3), *CsLD* (2), *CsFPA* (6), *CsFY* (2), *CsFLD* (6), *CsPCA* (2), *CsFRL* (4), *CsFRI* (8), *CsSUF4* (2), *CsFES1* (5), *CsEFS* (4), *CsPIE1* (1), *CsSWC6* (2), *CsARP6* (6), *CsFLC* (2), *CsSVP* (5), *CsSOC1* (1), *CsFUL* (2), *CsAP1* (2), *CsELF9* (21), *CsAGL19* (2), *CsTFL1* (1), *CsATC* (1), *CsLFY* (2), *CsVRN2* (6), *CsVEL1* (2), *CsMSI1* (5), *CsCLF* (2), *CsSWN* (2), *CsFIE1* (2), *CsVRN1* (4), *CsVRN5* (5), *CsLOM* (4), *CsSPL9* (4), *CsGI* (2), *CsAP2* (9) (*SNZ* and *SMZ* belong to AP2), *CsPHO2* (2), *CsAGL* (2), *CsAFB* (9), *CsARG* (7), *CsARF34* (10), *CsTCP4* (2), *CsLHP1* (2) and *CsHUA2* (3)(Table S23).

Those expanded include FT/TSF, FKF1, ZTL, CRY1/2, ELF3, PHYA, PHYB and PHYE in the photoperiod pathway, CIB1 in meristem identity, FLK and FRI in the autonomous pathway, MSI1 and VRN5 in the vernalization pathway, AFBs and ARG in the flowering pathway integrators (2:1, with 2 copies in Cs and 1 copy in rice)(Table S24). In addition, most of these family’s members belong to 3:1 or 4:1 type of genes. (Data S2). Os is a short-day plant but *C. songorica* is a long-day plant. Gene families in photoperiod pathway expanded most. For example, CO/FT module promotes flowering in response to changes in day length in Arabidopsis(Turck et al., 2008).

**2. Flower development**

Flower development are effectively explained by ABCDE model(O'Maoileidigh et al., 2014). Sepals (lemmas in rice) are regulated by A and E class gene; petals (paleas and lodicules in rice) are regulated by A, B and E class genes; stamens development are regulated by B, C and E class genes; carpels are controlled by C and E class genes; Ovules are controlled by C, D and E class genes(Theißen, 2001). *C. songorica* has two kinds flower (CH and CL flower) and all the flower organ in CH flower are larger than that of CL flower expect of filament. The anther and lodicule shape and size are distinct between CH and CL flower(Wu et al., 2018). To explain why CH and CL flower coexist and the flower organs differ in *C. songorica*, we identified genome-wide ABCDE model genes (AMGs) of *C. songorica* based on Os and *Arabidopsis* ABCDE model. Among 63 identified AMGs, 36 are A-class genes including 32 AP2 members and four MADS members, 4 B-class members, 7 C-class members, 6 D-class members, and 9 E-class members (Figure S28). The phylogenetic tree revealed that the same class genes largely cluster together (Figure S28).

**3.** **Flowering related transcription factors**

We identified 12 flowering related TF families in *C. songorica*, including B3(Shao et al., 2012), BBR-BPC(Kooiker et al., 2005), CO-like(Cheng and Wang, 2005), DBB(Kumagai et al., 2008), Dof(Diego Lijavetzky, 2003; Xu and Dai, 2016), EIL(Yamasaki et al., 2005), ERF(Nakano et al., 2006), GRF(Kim et al., 2003), LFY, MADS(Nam, 2003), MYB, NAC(Yujie Fang, 2008), SBP gene family. (Table S23). We totally identified seven CsDof family members, 45 CsERF family members, three CsGRF family members, 48 CsMYB family members, six CsSBP family members, 30 CsMADS family members, 39 CsNAC family members, two CsBBR/BPC family members, 11 CsB3 family members, two CsCO-like family members, five CsDBB family members and two CsEIL family members (Table S23).

**References**

Bäurle, I. and Dean, C. (2006) The Timing of Developmental Transitions in Plants. *Cell* **125**, 655-664.

Bottani, S., Zabet, N.R., Wendel, J.F. and Veitia, R.A. (2018) Gene Expression Dominance in Allopolyploids: Hypotheses and Models. *Trends Plant Sci* **23**, 393-402.

Cheng, X.F. and Wang, Z.Y. (2005) Overexpression of COL9, a CONSTANS-LIKE gene, delays flowering by reducing expression of CO and FT in Arabidopsis thaliana. *Plant J* **43**, 758-768.

Diego Lijavetzky, P.C., Jesús Vicente-Carbajosa (2003) Genome-wide comparative phylogenetic analysis of the rice and Arabidopsis Dof gene families. *BMC Evolutionary Biology* **3**.

Haider, W. (2014) Exploring flowering gene networks in Soybean and Arabidopsis through transcriptome analysis.

Jarvis, D.E., Ho, Y.S., Lightfoot, D.J., Schmockel, S.M., Li, B., Borm, T.J., Ohyanagi, H., Mineta, K., Michell, C.T., Saber, N., Kharbatia, N.M., Rupper, R.R., Sharp, A.R., Dally, N., Boughton, B.A., Woo, Y.H., Gao, G., Schijlen, E.G., Guo, X., Momin, A.A., Negrao, S., Al-Babili, S., Gehring, C., Roessner, U., Jung, C., Murphy, K., Arold, S.T., Gojobori, T., Linden, C.G., van Loo, E.N., Jellen, E.N., Maughan, P.J. and Tester, M. (2017) The genome of Chenopodium quinoa. *Nature* **542**, 307-312.

Jeong Hwan, L., Seong Jeon, Y., Soo Hyun, P., Ildoo, H., Jong Seob, L. and Hoon, A.J. (2007) Role of SVP in the control of flowering time by ambient temperature in Arabidopsis. *Genes Dev* **21**, 397-402.

Johanson, U., ., West, J., ., Lister, C., ., Michaels, S., ., Amasino, R., . and Dean, C., . (2000) Molecular analysis of FRIGIDA, a major determinant of natural variation in Arabidopsis flowering time. *Science* **290**, 344.

Kajitani, R., Toshimoto, K., Noguchi, H., Toyoda, A., Ogura, Y., Okuno, M., Yabana, M., Harada, M., Nagayasu, E., Maruyama, H., Kohara, Y., Fujiyama, A., Hayashi, T. and Itoh, T. (2014) Efficient de novo assembly of highly heterozygous genomes from whole-genome shotgun short reads. *Genome Res* **24**, 1384-1395.

Kim, J.H., Choi, D. and Kende, H. (2003) The AtGRF family of putative transcription factors is involved in leaf and cotyledon growth inArabidopsis. *The Plant Journal* **36**, 94-104.

Kooiker, M., Airoldi, C.A., Losa, A., Manzotti, P.S., Finzi, L., Kater, M.M. and Colombo, L. (2005) BASIC PENTACYSTEINE1, a GA binding protein that induces conformational changes in the regulatory region of the homeotic Arabidopsis gene SEEDSTICK. *Plant Cell* **17**, 722-729.

Kumagai, T., Ito, S., Nakamichi, N., Niwa, Y., Murakami, M., Yamashino, T. and Mizuno, T. (2008) The common function of a novel subfamily of B-Box zinc finger proteins with reference to circadian-associated events in Arabidopsis thaliana. *Biosci Biotechnol Biochem* **72**, 1539-1549.

Liu, L.J., Zhang, Y.C., Li, Q.H., Yi, S., Jian, M., Lian, H.L., Long, W. and Yang, H.Q. (2008) COP1-Mediated Ubiquitination of CONSTANS Is Implicated in Cryptochrome Regulation of Flowering in Arabidopsis. *Plant Cell* **20**, 292-306.

Michaels, S.D. and Amasino, R.M. (1999) FLOWERING LOCUS C encodes a novel MADS domain protein that acts as a repressor of flowering. *Plant Cell* **11**, 949-956.

Nakano, T., Suzuki, K., Fujimura, T. and Shinshi, H. (2006) Genome-wide analysis of the ERF gene family in Arabidopsis and rice. *Plant Physiol* **140**, 411-432.

Nam, J. (2003) Antiquity and Evolution of the MADS-Box Gene Family Controlling Flower Development in Plants. *Molecular Biology and Evolution* **20**, 1435-1447.

O'Maoileidigh, D.S., Graciet, E. and Wellmer, F. (2014) Gene networks controlling Arabidopsis thaliana flower development. *New Phytol* **201**, 16-30.

S, F., K, L., H, O., A, S., K, R., B, M., G, C. and J, P. (1999) GIGANTEA: a circadian clock-controlled gene that regulates photoperiodic flowering in Arabidopsis and encodes a protein with several possible membrane-spanning domains. *The EMBO Journal* **18**, 4679-4688.

Sascha, L., Virginie, M., José, L.G., José, G., Stephan, W., Jessika, A., Seonghoe, J., Carmen, K., Helen, B. and George, C. (2006) Arabidopsis SPA proteins regulate photoperiodic flowering and interact with the floral inducer CONSTANS to regulate its stability. *Development* **133**, 3213-3222.

Sawa, M., Nusinow, D.A., Kay, S.A. and Imaizumi, T. (2007) FKF1 and GIGANTEA complex formation is required for day-length measurement in Arabidopsis. *Science* **318**, 261-265.

Schnable, J.C., Springer, N.M. and Freeling, M. (2011) Differentiation of the maize subgenomes by genome dominance and both ancient and ongoing gene loss. *Proc Natl Acad Sci U S A* **108**, 4069-4074.

Shao, J., Liu, X., Wang, R., Zhang, G. and Yu, F. (2012) The over-expression of an Arabidopsis B3 transcription factor, ABS2/NGAL1, leads to the loss of flower petals. *PLoS One* **7**, e49861.

Smoczynska, A. and Szweykowska-Kulinska, Z. (2016) MicroRNA-mediated regulation of flower development in grasses. *Acta Biochim Pol* **63**, 687-692.

Sureshkumar, B., Sridevi, S., Janne, L. and Detlef, W. (2006) Potent induction of Arabidopsis thaliana flowering by elevated growth temperature. *Plos Genetics* **2**, e106.

Takato, I., Tran, H.G., Swartz, T.E., Briggs, W.R. and Kay, S.A. (2003) FKF1 is essential for photoperiodic-specific light signalling in Arabidopsis. *Nature* **426**, 302-306.

Teotia, S. and Tang, G. (2015) To bloom or not to bloom: role of microRNAs in plant flowering. *Mol Plant* **8**, 359-377.

Theißen, G. (2001) Development of floral organ identity: stories from the MADS house. *Current Opinion in Plant Biology* **4**, 75-85.

Turck, F., Fornara, F. and Coupland, G. (2008) Regulation and identity of florigen: FLOWERING LOCUS T moves center stage. *Annu Rev Plant Biol* **59**, 573-594.

Wu, F., Zhang, D., Muvunyi, B.P., Yan, Q., Zhang, Y., Yan, Z., Cao, M., Wang, Y. and Zhang, J. (2018) Analysis of microRNA reveals cleistogamous and chasmogamous floret divergence in dimorphic plant. *Sci Rep* **8**, 6287.

Xu, J. and Dai, H. (2016) Brassica napus Cycling Dof Factor1 ( BnCDF1 ) is involved in flowering time and freezing tolerance. *Plant Growth Regulation* **80**, 315-322.

Yamasaki, K., Kigawa, T., Inoue, M., Yamasaki, T., Yabuki, T., Aoki, M., Seki, E., Matsuda, T., Tomo, Y., Terada, T., Shirouzu, M., Tanaka, A., Seki, M., Shinozaki, K. and Yokoyama, S. (2005) Solution structure of the major DNA-binding domain of Arabidopsis thaliana ethylene-insensitive3-like3. *J Mol Biol* **348**, 253-264.

Yasui, Y., Hirakawa, H., Oikawa, T., Toyoshima, M., Matsuzaki, C., Ueno, M., Mizuno, N., Nagatoshi, Y., Imamura, T., Miyago, M., Tanaka, K., Mise, K., Tanaka, T., Mizukoshi, H., Mori, M. and Fujita, Y. (2016) Draft genome sequence of an inbred line of Chenopodium quinoa, an allotetraploid crop with great environmental adaptability and outstanding nutritional properties. *DNA Res* **23**, 535-546.

Yujie Fang, J.Y., Kabin Xie, Weibo Xie, Lizhong Xiong (2008) Systematic sequence analysis and identification of tissue-specific or stress-responsive genes of NAC transcription factor family in rice. *Molecular Genetics and Genomics* **280**, 547-563.
