# Supplementary material for: The genome of Cleistogenes songorica provides a blueprint for functional dissection of dimorphic flower differentiation and drought adaptability
Source: Plant Biotechnol J. 2020 Oct 28;19(3):532–47. doi: 10.1111/pbi.13483 (PMC7955882; doi:10.1111/pbi.13483)
Supplement: Supplementary file 2 — Material S2 Identification of flower development‐related genes. Table S1 Results of 17‐mer statistics. Table S2 Statistics of PacBio long reads assembly. Table S3 Assembly statistics of the C. songorica genome. Table S4 Coverage rate and mapping rate of Illumina reads aligned to assembly genome. Table S5 BUSCO analysis of C. songorica genome assembly in embryophyta. Table S6a Flower gene sets coverage rate in the C. songorica genome. Table S6b Leaves under drought tolerance gene sets coverage rate. Table S7 Coverage of gene function annotations from different sources. Table S8 Statistics of the predicted genes. Table S9 Statistics of the annotated RNAs. Table S10 Statistics of the repetitive elements. Table S11 Transposon elements prediction and statistics. Table S12 Correspondences of chromosome ID of the genome assembly and assigned sub‐genomes. Table S15 Nonsynonymous substitutions (Ka) and synonymous substitutions (Ks) values of PEPC homologous genes. Table S27 Cleistogenes accessions used for molecular marker analysis. [file PBI-19-532-s017.pdf]

Supporting Tables

Table S1 Results of 17-mer statistics.

K	K-mer_num	Peak_depth	Genome Size	Duplicate	Hete Rate
17	21,690,035,273	40	541,947,861	1.03%	0.16%

Table S2 The statistics of PacBio long reads assembly.

Type	Contig length (bp)	Contig number
N50	21,275,793	10
N60	19,463,092	13
N70	16,757,345	16
N80	12,620,938	20
N90	7,213,690	25
Longest	34,415,931	1
Total	540,114,186	118
Length>=1kb	540,114,186	118
Length>=2kb	540,114,186	118
Length>=5kb	540,114,186	118

Table S3 Assembly statistics of the *C. songorica* genome.

Assembly	Size (bp)		
Contigs N50	21,275,793		
Superscaffold N50	21,275,793		
Total length	540,116,881		
Hi-C total sequences ordered and oriented (ratio %)	528,521,744 (97.85%)		
Annotation	number	Total length (bp)	Alignment rate (%)
GC content			45.02%
Protein-coding genes	54,383		
Annotated genes	48,660		
Gene mapped ratio	52,223		96.03%
Orphans genes	3,180		
Complete BUSCOs			98.25% (embryophyta)
Exons	276,708	123,750,763	
Introns	222,325	114,903,270	
miRNA	287	36,534	
LncRNA	3,397		
Repeat class		Total length (bp)	Genome percentage (%)
TEs		228,103,708	41.99
LTR		144,136,979	26.54
LINE		22,619,367	4.16
SINE		122,365	0.023
DNA		56,162,821	10.34

Table S4 Coverage rate and mapping rate of Illumina reads aligned to assembly genome.

Library	Depths	Covrage1x	Covrage5x	Covrage10x	Mapping rate
450bp	113×	98.58%	98.32%	98.13%	99.87%

Table S5 BUSCO analysis of *C. songorica* genome assembly in embryophyta.

Type	Number (Percentage)
Complete BUSCOs	1351 (98.3%)
Complete and single-copy BUSCOs	464 (33.7%)
Complete and duplicated BUSCOs	887 (64.5%)
Fragmented BUSCOs	0.2 (1%)
Missing BUSCOs	21 (1.5%)
Total BUSCO groups searched	1375

Table S6a Flower gene sets coverage rate in the *C. songorica* genome.

Dataset	Number	Total length(bp)	Bases covered by assembly (%)	Sequences covered by assembly (%)	with >90% sequence in one scaffold		with >50% sequence in one scaffold	
					Number	Percent (%)	Number	Percent (%)
>0bp	69331	52,761,155	97.19	98.59	63485	91.56	68092	98.21
>200bp	69331	52,761,155	97.19	98.59	63485	91.56	68092	98.21
>500bp	32693	41,729,224	97.21	99.16	29171	89.22	32304	98.81
>1000bp	17560	30,866,685	97.44	99.82	15581	88.73	17478	99.53

Table S6b Leaves under drought tolerance gene sets coverage rate.

Dataset	Number	Total length(bp)	Bases covered by assembly (%)	Sequences covered by assembly (%)	with >90% sequence in one scaffold		with >50% sequence in one scaffold	
					Number	Percent (%)	Number	Percent (%)
>0bp	1499	1,005,949	88.82	90.86	1317	87.85	1351	90.12
>200bp	1474	1,003,612	88.81	90.77	1304	88.46	1327	90.02
>500bp	1308	939,848	88.74	90.51	1153	88.14	1173	89.67
>1000bp	44	53,093	96.27	100	40	90.9	44	100

Table S7 Coverage of gene function annotations from different sources.

		Number	Percent (%)
	Total	54383	
Annotated	InterPro	38458	70.7169
	GO	29038	53.3953
	KEGG	27951	51.3965
	Nt	48305	88.8237
	Nr	47863	88.0109
	COG	17865	32.8503
	Swissprot	35248	64.8143

TrEMBL	47453	87.257
Total_Annotated	48660	89.4764
Unanotated	5723	10.5235

Table S8 Statistics of the predicted genes.

Gene set		Number	Average transcript length (bp)	Average CDS length (bp)	Average exon per gene	Average exon length (bp)	Average intron length (bp)
Homolog	*H.vulgare*	89,924	1600.978	850.8154	3.1838	267.2336	343.5143
	B.distachyon	72,216	1936.6775	919.2453	3.7564	244.7137	369.1148
	Z.mays	77,435	2054.0584	1022.9419	3.8024	269.0279	367.9453
	O. thomaeum	59,604	2305.771	901.801	4.0257	224.0089	464.0093
	O.sativa	75,545	2104.3294	1014.6224	3.8436	263.9778	383.2152
	S. bicolor	72,503	2021.0759	939.9134	3.8734	242.6603	376.2698
De novo	fgenesh	56,294	2877.7787	1124.1174	5.193	216.4669	418.2332
	Augustus	67,912	2974.3214	1360.456	6.1793	220.1633	311.5988
	Genescan	56,666	6534.8464	1357.8715	6.9789	194.5688	865.8776
Glean		55,115	3550.9338	1170.2213	5.1948	225.2689	567.5424
Final set		54,383	3450.1624	1174.6211	5.0881	230.855	516.8257

Table S9 Statistics of the annotated RNAs.

Type		Copy(w)	Average length(bp)	Total length(bp)	Percentage of genome
miRNA		287	127.2962	36,534	0.0067
tRNA		1,139	74.9122	85,325	0.0157
rRNA	rRNA	580	281.2345	163,116	0.03
	18S	99	1025.9495	101,569	0.0187
	28S	232	135.9957	31,551	0.0058
	5.8S	65	136.5538	8,876	0.0016
	5S	184	114.7826	21,120	0.0039
snRNA	snRNA	932	110.0966	102,610	0.0189
	CD-box	716	100.7514	72,138	0.0133
	HACA-box	74	127.8243	9,459	0.0017
	splicing	142	147.9789	21,013	0.0039

Table S10 Statistics of the repetitive elements.

Type	Repeat Size(bp)	% of genome
TRF	12,906,616	2.3761
RepeatMasker	87,559,262	16.1194
RepeatProteinMask	54,177,654	9.9739
De novo	203,718,893	37.504
Total	221,293,093	40.7393

Table S11 Transposons elements prediction and statistics.

Type	Repbase TEs		TE protiens		De novo		Combined TEs	
	Length (bp)	% of genome	Length (bp)	% of genome	Length (bp)	Percent (%)	Length (bp)	% of genome
DNA	25,129,355	4.63	10,721,166	1.97	45,669,180	8.41	56,162,821	10.34
LINE	13,955,376	2.57	11,657,003	2.15	17,135,069	3.15	22,619,367	4.16
SINE	23,837	0.0044	0	0	103,071	0.019	122,365	0.023
LTR	48,175,067	8.87	31,799,822	5.85	141,220,445	25.99	144,136,979	26.54
Other	2,238	0.0004	0	0	0	0	2,238	0.0004
Unknown	387,772	0.071	10,983	0.002	4,661,741	0.86	5,059,938	0.93
Total	87,673,645	16	54,188,974	10	208,789,506	38	228,103,708	42

Table S12 The correspondences of chromosome ID of the genome assembly and assigned sub-genomes.

	Chromosome number									
Genome	Cs01	Cs04	Cs05	Cs06	Cs11	Cs12	Cs14	Cs15	Cs16	Cs20
A sub-genome	A1	A2	A3	A4	A5	A6	A7	A8	A9	A10
Genome	Cs03	Cs07	Cs02	Cs08	Cs09	Cs18	Cs13	Cs10	Cs17	Cs19
B sub-genome	B1	B2	B3	B4	B5	B6	B7	B8	B9	B10

Table S15 Nonsynonymous substitutions (Ka) and synonymous substitutions (Ks) values of PEPC homologous genes.

Homologous ID		Type	Ka	Ks	Ka/Ks
CCG032185.1	CCG014840.1	C4	0.0169	0.0852	0.1989
CCG024684.1	CCG000881.1	C3	0.0284	0.1078	0.2637
CCG051083.1	CCG014028.1	C3	0.0108	0.1261	0.0853
CCG024684.1	CCG000881.1	C3	0.0284	0.1078	0.2637
CCG051083.1	CCG014028.1	C3	0.0108	0.1261	0.0853
CCG052768.1	CCG014840.1	C3	0.6053	2.0972	0.2886
CCG052768.1	CCG015562.3	C3	1.0735	1.6969	0.6326

Table S27 *Cleistogenes* accessions used for molecular marker analysis.

Population	Species	Origin	Cultivated/wild	Sampling site		
				Latitude	Longitude	Altitude(M)
Acce01	*C. caespitosa*	Wuquanshan, Gansu, China	Wild	E103 ˚50′ 23 "	N36 ˚0′ 45 "	2045
Acce02	*C. songorica*	Qingbaishi, Gansu, China	Wild	E103 ˚55′ 33 "	N36 ˚5′ 23 "	1589
Acce03	*C. songorica*	Yuzhong, Gansu, China	Wild	E104 ˚8′ 17 "	N35 ˚56′ 38 "	1940
Acce04	*C. songorica*	Alxa League Left Banner, Inner Mongolia, China	Wild	E105 ˚46′ 36 "	N38 ˚52′ 35 "	1786
Acce05	*C. songorica*	Alxa League Left Banner, Inner Mongolia, China	Wild	E105 ˚45′ 7 "	N38 ˚55′ 52 "	1569
Acce06	*C. songorica*	Alxa League Left Banner, Inner Mongolia, China	Wild	E105 ˚44′ 40 "	N38 ˚55′ 50 "	1551

Acce07	*C. songorica*	Alxa League Left Banner, Inner Mongolia, China	Wild	E105 °50′ 36 "	N38 °51′ 50 "	2007
Acce08	*C. songorica*	Alxa League Right Banner, Inner Mongolia, China	Wild	E101 °40′ 1 "	N39 °12′ 29 "	1891
Acce09	*C. songorica*	Alxa League Right Banner, Inner Mongolia, China	Wild	E101 °33′ 37 "	N38 °46′ 54.3 "	2016
Acce10	*C. songorica*	Minqin, Gansu, China	Cultivated	E104 °12 " 10 "	N39 °27 " 37 "	1400
Acce11	*C. songorica*	Xilingol League, Inner Mongolia, China	Wild	E105 °33′ 742 "	N39 °5′ 85 "	1370
Acce12	*C. songorica*	Xilingol League, Inner Mongolia, China	Wild	E105 °33′ 375 "	N39 °16′ 115 "	1360
Acce13	*C. songorica*	Alxa League Left Banner, Inner Mongolia, China	Wild	E105 °44′ 272 "	N38 °52′ 272 "	1730
Acce14	*C. songorica*	Alxa League Ejina Banner, Inner Mongolia, China	Wild	E105 °48′ 67 "	N38 °56′ 2 "	1700
Acce15	*C. songorica*	Alxa League Ejina Banner, Inner Mongolia, China	Wild	E105 °45′ 480 "	N38 °52′ 217 "	1810
Acce16	*C. songorica*	Alxa League Ejina Banner, Inner Mongolia, China	Wild	E105 °48′ 67 "	N38 °56′ 2 "	1700
Acce17	*C. songorica*	Alxa League Ejina Banner, Inner Mongolia, China	Wild	E105 °40′ 951 "	N39 °01′ 95 "	1410
Acce18	*C. songorica*	Alxa League Left Banner, Inner Mongolia, China	Wild	E105 °32′ 805 "	N39 °01′ 740 "	1370
Acce19	*C. songorica*	Alxa League Left Banner, Inner Mongolia, China	Wild	E105 °43′ 559 "	N38 °52′ 661 "	1680
Acce20	*C. songorica*	Xilingol League, Inner Mongolia, China	Wild	E105 °14′ 329 "	N39 °3′ 751 "	1370
Acce21	*C. hackelii*	Shandong, China	Wild		unknown	
Acce22	*C. hancei*	Shandong, China	Wild	E121 °15′ 45 "	N37 °29′ 49 "	5
Acce23	*C. squarrosa*	Mongolia	Wild		unknown	

Table S13 KEGG enrichment of homologues genes, with the ration of 4:1 or 3:1 (*C.songorica* : *O. sativa*) and (*C. songorica* : *O. thomaeum*).

Table S14 KEGG enrichment of the *C. songorica* unique genes.

Table S16 List of conserved drought-responsive genes shared across 4 Poaceae species and their expression patterns in *C. songorica*.

Table S17 Representatives of the predicted interaction networks among lncRNAs, Poaceae conserved genes and miRNAs.

Table S18 Gene list of conserved genes and expanded genes.

Table S19 Significantly expanded genes.

Table S20 Flowering related genes information.

Table S21 Homologous genes between *C. songorica* and *O. sativa*.

Table S22 ABCDE-MADS genes expression.

Table S23 Transcription factors of ABCDE-MADS and miRNA expression.

Table S24 TFs binding site in AMGs promoter region.

Table S25 The expression level of *CsSPL*s in different tissues.

Table S26 The expression of MYB family members. (a) MYB family members expression level in different tissues. LS: light stress, SS: severe stress, RW1: re-water. (b) Differential expressed genes under drought stress in shoot and flower with expression level >1.

Data S1 Single copy genes from *C. songorica* and other nine species.

Data S2 Gene families identified using OrthoMCL in ten species.

Data S3. Gene IDs of the copy number ratio between O. sativa vs C. songorica = 1:3, 1:4 and 1:5.
